# A double blind placebo controlled randomized trial of the effect of acute uric acid changes on inflammatory markers in humans: A pilot study

**DOI:** 10.1371/journal.pone.0181100

**Published:** 2017-08-07

**Authors:** Toshiko Tanaka, Yuri Milaneschi, Yongqing Zhang, Kevin G. Becker, Linda Zukley, Luigi Ferrucci

**Affiliations:** 1 Translational Gerontology Branch, National Institute on Aging, Baltimore, Maryland, United States of America; 2 Department of Psychiatry, Amsterdam Public Health, VU University Medical Center, Amsterdam, The Netherlands; 3 Laboratory of Genetics and Genomics, National Institute on Aging, National Institutes of Health, Baltimore, Maryland, United States of America; Kurume University School of Medicine, JAPAN

## Abstract

Uric acid has been linked with increased risk of chronic disease such as cardiovascular disease and this association has been attributed to a pro-inflammatory effect. Indeed, observational studies have shown that high uric acid is associated with high level of pro-inflammatory cytokines in the blood. However, whether high uric acid directly affects inflammation or rather represents a parallel defensive antioxidant mechanism in response to pathology that causes inflammation is unknown. To determine whether acute increase or decrease uric acid levels affects inflammation in healthy individuals, a randomized, placebo-controlled, double blind clinical study of uric acid or rasburicase with 20 healthy volunteers in each treatment-placebo group was conducted at the National Institute on Aging (NIA) Clinical Research Unit (CRU) at Harbor Hospital in Baltimore, MD. Change in inflammatory response was assessed by administering an oral lipid tolerance before and after the treatment of uric acid, rasburicase and placebo. Following uric acid administration, there was an accentuated increase in IL-6 during the oral lipid tolerance test (P<0.001). No significant differences were observed after lowering of uric acid with rasburicase. No side effects were reported throughout the trial. In health individuals, acute increase in uric acid results in an increased IL-6 response when challenged with lipid load. Such effect of amplification of inflammatory response may explain the higher risk of chronic diseases observed in subclinical hyperuricemia in observational studies.

Trial Registration: ClinicalTrials.gov NCT01323335

## Introduction

There is growing interest in understanding the role of uric acid (UA) in inflammation, and chronic diseases [1, 2]. Uric acid is a product of purine metabolism with strong antioxidant properties found in high levels in humans due to the silencing of the uricase gene in the course of evolution [3, 4]. It has been proposed that the high level of UA was selected in humans because UA has powerful antioxidant properties [5]. However, contrary to this view, there is ample evidence that even moderately elevated levels of uric acid have detrimental effects on health. In observational studies, hyperuricemia is an independent risk factor for diabetes [6], impaired fasting glucose [7], insulin resistance [8], and hypertension in the general population [7, 9]. In addition to a causative role in gout, high UA concentrations have been associated with recurrence of stroke, high risk of cardiovascular events, congestive heart failure, hypertension and chronic kidney disease [10, 11]. Moreover, UA is a strong negative prognostic factor in patients affected by chronic heart failure [12], and an independent predictor of cardiovascular [13], and all-causes mortality [14]. Interestingly, the mechanism of these associations and whether uric acid plays a causal role in the pathogenesis of those diseases remain unclear.

Inflammation is one of the causal factors that may be the link between UA with chronic diseases. Pre-clinical and clinical studies suggest that UA is a strong pro-inflammatory factor. In a large representative sample of community-dwelling elderly participants, abnormally high UA levels as well as UA concentrations in the upper portion of the physiologic range are associated with high levels of several inflammatory markers, such us White Blood Count (WBC), neutrophils, C-reactive protein (CRP), interleukin-6 (IL-6), interleukin-18 (IL-18), interleukin-1 receptor antagonist (IL-1ra), and tumor necrosis factor-alpha (TNF-a) [2]. Furthermore, baseline UA concentrations and UA changes significantly predicted IL-6 and CRP levels after three years [15]. These findings are consistent with the association between serum UA and CRP levels which has been described in previous epidemiological studies [16].

Contrary to the view that high uric acid may have negative effects on health a handful of observational and intervention studies have shown positive health effects of UA. Lower circulating UA has been reported in several neurological diseases including multiple sclerosis, Alzheimer’s disease [17–19], dementia [20], Parkinson’s disease [21–23] and amyotrophic lateral sclerosis [24]. It is unclear whether UA can prevent the development of these conditions, however studies have shown that higher uric acid is associated with a slower progression of these neurological diseases [25, 26]. In addition to neurological conditions, UA has been shown to slow down the decline of muscle strength experienced by the majority of aging individuals [26–28]. The mechanism for the neuro-muscular protective effect of uric acid is unclear, and it has been suggested that uric acid may serve as an antioxidant that protects the brain and muscle from oxidative stress. To this end, studies have shown that acute increase through administration of uric acid lead to increase in antioxidant capacity and reduces exercise-induced oxidative stress [29, 30]. Since reactive oxygen species (ROS) can trigger an inflammatory response via activation of the Nuclear Factor k-B (NF-kB) pathway, researches have hypothesized that blocking ROS through UA would have an anti-inflammatory effect.

The intra-articular pro-inflammatory activity of monosodium urate crystals, the saline form of UA involved in the pathogenesis of gout, has been extensively investigated. Whether increased levels of soluble UA can trigger a systemic inflammatory response remains unclear. At present, the evidence in favor of the pro-inflammatory role of soluble UA is limited to few studies performed in vitro and in experimental animals and no robust data is available from human models.

Understanding whether high level of UA determine a pro-inflammatory state has important clinical implications. Overtly elevated levels of UA, should be treated to prevent the formation of urate crystals in the synovial liquid. However, whether UA levels in the upper portion of the normal range should be treated is uncertain. If UA does not contribute to the onset/worsening of inflammation but rather limit the ROS damage in particular pro-inflammatory states, then treatment should be held. On the contrary, if soluble UA directly stimulates the inflammatory response and this potentially harmful mechanism overcomes the potential benefits related to antioxidant activity, then the treatment should be started early, when UA concentration are still in the upper portion of the normal range. Observational studies cannot fully address this question. In fact, we cannot exclude that the cross-sectional and longitudinal association between UA, inflammatory markers and negative health outcomes, may simply reflect the fact that UA is an inducible antioxidant that is produced in response to increasing oxidative stress.

To verify the hypothesis that UA activates inflammation, we conducted two complementary randomized controlled studies, each one including 10 treated and 10 placebo control subjects. In the first study, subjects with low UA were administered 500 mg of UA intravenously. In the second study, subjects with moderately elevated UA were administered a single acute dose of Rasburicase. Then inflammatory markers were measured at multiple points in time, for a total of 32 hours. We hypothesized that acute increase and reduction of UA levels would be followed, respectively, by increasing and decreasing levels of inflammatory markers. We also studied where changes in UA levels were associated with lower or higher inflammatory response during a lipid tolerance test used an acute pro-inflammatory stimulus that often occur in normal daily life.

## Material and methods

The study protocol was approved by the Institutional ethics committee at Medstar Research Institute and each participant gave written informed consent before enrollment. This study was a placebo-controlled double blind randomized trial carried out at the National Institute on Aging (NIA) Clinical Research Unit (CRU) at Harbor Hospital in Baltimore, MD.

### Human subjects

Twenty male and twenty female subjects were recruited in the study that consists of two inter-related however independent study, Study A: Uric acid versus Placebo (n = 20) and Study B: Rasburicase versus Placebo (n = 20), respectively. Participants were community-dwelling women and men with the ability to fully participate in an informed consent process. Eligibility criteria included age 50–75 years, BMI 23–34.9 kg/m^2^, glomerular filtration rate (GFR) > 60mL/min, and Blessed mental score [31] equal to or less than 3. For study A, subjects were eligible if they had a uric acid ≤ 10 mg/dL. For study B, the initial inclusion criteria required participants to have uric acid levels between 7-10mg/dL. After difficulties recruiting subjects meeting this criterion, the range was changed to 6-10mg/dL (May 2010), then changed again to ≤ 10mg/dL (August 2010). Participants randomly assigned to study B were excluded if they had glucose-6-phosphate dehydrogenase deficiency because they cannot break down the hydrogen peroxide produced during the conversion of UA into soluble allantoin, leading to hemolysis and especially methemoglobin formation [32]. The screening for both studies were conducted between October 2009-February 2011.

### Drug information

In this study, 500mg of uric acid (UA) was administered solubilized into 250mL of 0.1% lithium carbonate/4% dextrose as previously described [30]. Before the study began, UA solubility studies were conducted by Florida Biologix (Alchua, FL) to determine whether sodium bicarbonate could be used in the place of lithium carbonate as a solubility medium, therefore avoiding the interference of lithium carbonate. UA was not soluble in sodium bicarbonate solution ranging from 0.031–1% and lithium bicarbonate solution less than 0.1% within biologically safe pH levels. Therefore, the final UA assembly kit which consisted of powdered 500mg of UA (Sigma Aldrich), 250mL of 4% dextrose solution, 250mg dry lithium carbonate and a final assembly bag was prepared by Florida Biologix (Alchua, FL). UA was tested for purity at 99.9% in line with GMP. The dry chemicals were gamma irradiated (Gamma Irradiation Facility, Charlotte, NC) and solutions were tested for pH, sterility and endotoxin to ensure sterility of the final product. The UA and placebo were prepared by a pharmacist on the CRU at Harbor Hospital within 60 minutes of administration according to the manufacturers’ instructions. The final concentrations was 500mg of uric acid in 250ml of 0.1% lithium carbonate/ 4% dextrose solution, and the placebo was 250ml of the same medium without the uric acid. The use of uric acid as an investigational new drug (IND) was approved under section 505(i) of the Federal Food, Drug, and Cosmetic Act for Uric Acid (IND reference number 103022).

Rasburicase (RAS) is a recombinant urate oxidase cloned from a strain of *Aspergillius flavus* is produced by a genetically modified Saccharomyces cerevisiae strain. This drug sold under the name Elitek^®^ is manufactured and was donated by Sanofi-Synthelabo, Inc (Bridgewater, NJ) for use in this study. RAS is supplied in form of sterile, lyophilized powder intended for intravenous administration. The product was reconstituted and visually inspected for particulate matter and discoloration prior to administration. The solution was diluted in 250-mL of preservative-free 0.9% sodium chloride (NaCl) was administered within 24 hours of reconstitution. The NaCl solution without the RAS was used for placebo. The dose used for this study was 0.15mg/kg/day which is on the lower end of the recommended dosage of 0.15–0.2mg/kg/day in adult and children.

### Study protocol

This study protocol involved the participants to come in to NIA CRU for 4 days including one overnight stay (S1 Fig). Participants were initially invited to the CRU for a screening visit (V1) following an overnight fast to assess baseline characteristics and eligibility criteria. During this visit, trained nurse practitioners collected a detailed medical history, performed a physical exam, and measured height, weight (used to compute BMI) and waist circumference. Cognitive status was assessed using the Blessed Mental Test [31] and those who made more than 3 errors were excluded from the study. Participants who were eligible were scheduled for the second visit within a week of V1 and instructed to maintain the same dietary habits throughout the course of the study, in particular not to alter their consumption of high purine foods.

In the second visit (V2), the participants arrived at the CRU after an overnight fast for 8 hours for pre-intervention assessment of postprandial inflammatory response. The Oral Lipid Tolerance Test (OLTT) consists of eating a standard fast-food type meal (with approximately 900 kal and 50 grams of fat). The meal was prepared by a registered dietitian and was consumed in no longer than 20 minutes. Following the meal, the subjects did not consume anything except for water and ice for 8 hours. Levels of inflammatory markers (IL-6, IL-6sr, sgp 130, and high-sensitivity CRP) were assessed at baseline and at 2 hour intervals for total of 8 hours.

Participants were invited back for the third visit two days after V2. This visit was conducted over 2 consecutive days with an overnight stay at the CRU. The intravenous administration of UA or RAS and respective placebo were administered on the first day of visit 3 (V3d1). Participants were instructed to drink at least two glasses of water and eat a light breakfast (cold cereal, milk, coffee or tea and bread and butter with butter and/or jelly in the morning) before the intervention. For the both the UA study and RAS study, five women and men were randomly assigned either the intervention or placebo solution that was infused using an IV infusion pump over a 60 minute. For allocation of participants, a simple randomization (1:1) was conducted using a computer-generated list of random numbers. The randomization list was maintained by the protocol study monitor, two sealed copies of the randomization list was stored at the NIA: one with the PI and one secured in the NIA Research Pharmacy. Study investigators and participants were blinded to the treatment and placebo status. However, the NIA research pharmacist was not blinded since the medication and placebo were prepared by them. For both studies, treatment and placebo administration was done conducted under the continuous supervision of the study physician and the solution prepared and labelled by a research pharmacist. During the interventions, blood sampling was done using an IV access different from the one used for infusing the study drug. An intravenous cannula was inserted into a large vein using a standard aseptic technique and, if requested from participants, a local anesthesia. The IV site was kept open by a KVO rate for continuous saline solution infusion, and was used to draw blood samples. The cannula was removed at the end of the visit. Blood samples were drawn at baseline, after 1, 2, 4, 8, 12 and 24 hours and after 2 weeks to measure uric acid, inflammatory markers (hsCRP, sgp130, IL-6, IL-6sr, TNFα-RI, TNFαRII, IL-18, IL-1β, TNFα, and IL1ra), and oxidative stress markers (ratio of glutathione reduced: oxidized [GSSH: GSH], F2-isoprostane [iPF2a-III]) The 24 hour reading was the baseline for the second day of visit 3 (V3d2). On this day, a second OLTT was performed following the same protocol as V2.

The final visit (V4) took place 2 weeks following the third visit and was mostly a safety visit checking for possible side effects. In addition, fasting blood drawn was performed for the assessment of inflammatory markers hsCRP, sgp130, IL-6, IL-6sr, TNFα-RI, TNFαRII, IL-18, TNFα, and IL1ra. No harms were reported throughout the study protocol.

### Inflammatory and oxidative damage marker

Blood levels of inflammatory markers were measured using nephelometry (hs-CRP; Seimens BNII), ELISA (IL-6sr, IL-6, TNFα-RI, TNFαRII, sGP130, IL-18; R&D Systems), Luminex xMAP immunoassay technology (TNFα, IL1ra; Millipore). Interassay coefficient of variation for these inflammatory markers ranged from 2.3–5.2% (hs-CRP), 5.2–10.3% (IL-6sr), 3.3–14.2% (IL-6), 2.8–5.8% (sGP130), 1.7–4.1% (TNFα-RI), 5.1–8.6% (TNFαRII), 2–12% (IL-18), 3.6–8.5% (TNFα), and 3.6–11% (IL1ra). Oxidative stress markers F2-isoprostane (iPF2a-III) and reduced (GSH) and oxidized glutathione (GSSG) was measured using LC-MS-MS (Kronos Science Laboratory, Pheonix AZ).

### Gene expression

Whole Blood samples for microarray RNA expression were collected in PAXgene (QIAGEN/BD) tubes at baseline, and at 12, and 24 hours after the beginning of the infusion of intervention drugs on V3d. RNA was extracted using PAXgene blood mRNA kit (Qiagen, Crawley, UK) according to the manufacturers’ instructions. The extracted total RNA was then used to create biotin-labelled single stranded RNA (cRNA) with the Illumina TotalPrep RNA Amplification Kit. Briefly, 0.5ug of total RNA was converted to single stranded complementary DNA (cDNA) with reverse transcriptase using an oligo-dT primer with T7 RNA polymerase promoter site then copied to create double-stranded cDNA. Using the supplied column, these double-stranded cDNA is cleaned and concentrated overnight using to generate cRNA with biotin-16-UTP in a *in vitro* transcription reaction. A total of 0.75ug of biotin-labelled cRNA was hybridized at 58°C for 16h to Illumina’s Sentrix Human HT-12 v3 Expression BeadChips (Illumina, San Diego). Each microarray chip measures expression levels of 48,000 transcripts with approximately 15-fold redundancy. The arrays were washed, blocked, and the labelled cRNA was detected by staining with streptavidin-Cy3. Hybridized arrays were scanned using an Illumina BeadStation 500X Genetic Analysis Systems scanner and the image data extracted using the Illumina GenomeStudio software, version 1.1.1) [33]. Quality control of the data was conducted using R package limma [34]. Probes were excluded if they were not significantly expressed at a detection p-value of P<0.01 in at least 5% of the total sample (19472/47323). The expression signals of probes that passed QC were quantile normalized to the median distribution and subsequently log2-transformed. The probe and sample means were centered to zero.

### Statistical analysis

Sample size was calculated for linear mixed model with random slope using the package longpower in R [35–37]. The calculation was based on previous findings from Waring and our group where infusion of 1,000 mg and 500 mg of UA were associated with an increase of UA circulating levels of 3.2–4.9 mg/dL with this increase persisting for 10–12 hours [29, 30, 38]. Further, the report by Ruggiero et al found that IL6 increased by 0.08 pg/mL of IL-6 per mg/dL increase of uric acid [2]. Thus, we anticipated a slope in the UA group of approximately 0.039 pg/mL of IL-6 per hour (0.08*4.9/10) and a slope of 0 for the control group. For a study with baseline and 1-, 2-, 4-, 8-, and 12-hour follow-up measurements, and assuming a standard deviation of 0.7 and within-person correlation of 0.8, we calculated that a sample size of 10 participants per group was sufficient to detect a difference in slopes of 0.039 pg/mL of IL-6 per hour with 82% power using a two-sided test with type I error of 5%.

For all analysis, the main comparisons were between the drug and placebo group at each arm of the study (uric acid or rasburicase). Differences in baseline characteristic and inflammatory markers and at both baseline (V1 or V2 before OLTT) and final visit (V4) were assessed using Mann-Whitney U test. Changes in inflammatory markers during a 12 hour period after the infusion of test drug on V3d1 and postprandial changes over the 8 hours following the lipid load in OLTT on V2 and V3d2 were analyzed by repeated measure mixed models using the MIXED procedure in SAS (Windows version 9.3; Cary Institute) using unstructured correlation structure. The hypotheses tested included the non-linear effect of time (P_time_) tested by creating a dummy variable for each time point, uric acid or rasburicase treatment group (P_tx_) and the interaction of the two (P_int_) on inflammatory and oxidative stress markers. Differences between each treatment group at each time point was assessed using the ESTIMATE statement under the mixed procedure. In this analysis, a second analysis testing for the linear effect of time was also examined. For the OLTT, we further tested the differences in postprandial changes in inflammatory markers before and after the intervention within the treatment or control group using the CONTRAST statement. Covariates in the models included age, sex and body mass index. For all analyses, P<0.05 was considered statistically significant.

To test for changes in gene expression profile in each study, changes in expression level of each probe, a repeated measure analysis using the three time points was conducted using linear mixed model to test for the non-linear and linear effects of time, treatment group and the interaction of the two for each probe using the lmer function in the R lme4 package (http://cran.r-project.org/). Covariates in the model included age in years, sex, cell composition (percentage of eosophil, lymphocytes, monocytes, neutrophils), and array batch. Genome-wide significance was considered at P≤2.57x10^-6^. From these analyses, the ranked gene lists were used to test for enrichment of Gene Ontology (GO) terms using the online software tool GOrilla [39, 40]. The analysis was run with single ranked list of genes which does not require a p-value cut off and analyzes the full set of genes on the array.

## Results

### Study characteristics

**Fig 1:**
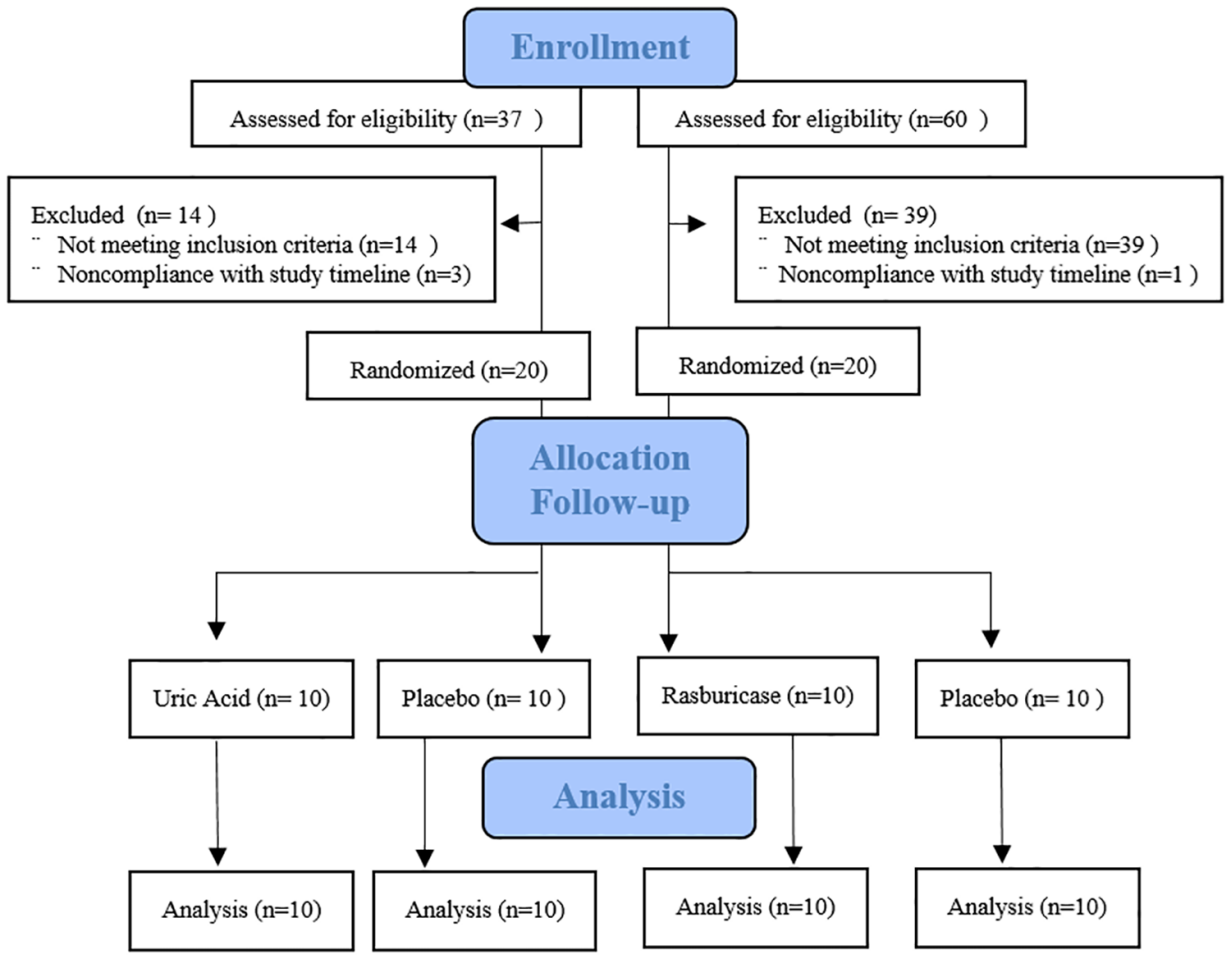
COSORT flowchart for the uric acid and rasburicase study.

For the Uric acid arm of the study, 37 participants were screened, 23 were eligible and randomized to placebo or uric acid and 20 were allocated the intervention. For the rasburicase arm of the study, 60 subjects were screened, 21 were eligible to either rasburicase or placebo and 20 were randomized the intervention (Fig 1). Four subjects were randomized but did not receive the intervention due to non-compliance in the study timeline (i.e. did not show up on the study protocol day). At baseline, participants in the uric acid study treatment group were slightly older (56.7±6.2 vs 63.4±7.3, p = 0.045) and had higher BMI (25.9±2.2 vs 27.8±1.2, p = 0.011; Table 1). In the Rasburicase study, those in the treatment group had higher systolic blood pressure compared (123.6±10.7 vs 134.9±9.2, p = 0.041), BMI (26.9±2.4 vs 27.3±2.1, p = 0.049) to the placebo group.

**Table 1:** Characteristics of participants in the uric acid and rasburicase study.

	Uric Acid					Rasburicase				
	Placebo (N = 10)		Treatment (N = 10)			Placebo (N = 10)		Treatment (N = 10)		
	Mean	std	Mean	std	P^1^	Mean	std	Mean	std	P^2^
Age (years)	56.7	(6.2)	63.4	(7.3)	0.045	56.8	(4.3)	56.0	(5.4)	0.143
LDL Cholesterol (mg/dL)	131.8	(13.6)	123.1	(20.4)	0.528	152.2	(17.9)	141.9	(33.8)	0.670
HDL Cholesterol (mg/dL)	60.0	(10.7)	71.0	(16.8)	0.050	56.5	(14.0)	52.9	(13.2)	0.280
Total Cholesterol (mg/dL)	212.9	(12.8)	212.7	(14.7)	0.812	231.5	(19.3)	222.3	(40.7)	0.839
Uric Acid (mg/dL)	4.5	(0.8)	4.3	(0.7)	0.642	5.6	(1.8)	6.2	(1.5)	0.541
WBC (n, K/μL)	6.0	(0.7)	6.4	(2.1)	0.517	6.1	(1.6)	5.4	(1.2)	0.517
Hemoglobin (g/L)	13.8	(0.8)	14.2	(1.5)	0.424	14.2	(1.2)	14.5	(1.3)	0.381
Systolic BP (mmHg)	131.2	(12.5)	135.1	(16.1)	0.469	123.6	(10.7)	134.9	(9.2)	0.540
Dyastolic BP (mmHg)	78.0	(8.5)	74.4	(9.5)	0.210	69.5	(5.8)	75.3	(9.5)	0.041
BMI (kg/m2)	25.9	(2.2)	27.8	(1.2)	0.011	26.9	(2.4)	27.3	(2.1)	0.049
CRP (ug/mL)	1.0	(0.9)	1.8	(0.9)	0.505	4.7	(6.7)	2.9	(1.5)	0.591
IL-6 (pg/mL)	2.5	(1.6)	2.4	(1.4)	1.000	2.7	(1.5)	2.3	(1.1)	0.796
IL-6sr (pg/mL)	38112	(9207)	40744	(12052)	0.408	33799	(8747)	32903	(8171)	0.796
sGP-130 (ng/mL)	242.5	(26.5)	257.3	(27.3)	0.173	271.1	(58.4)	252.2	(37.4)	0.529
TNFa (pg/mL)	2.8	(0.8)	3.1	(0.9)	0.853	1.9	(1.5)	1.8	(0.8)	0.780
TNFaRI (pg/mL)	1335	(201)	1481	(324)	0.393	1229	(191)	1328	(184)	0.280
TNFaRII (pg/mL)	2624	(302)	2721	(709)	1.000	2331	(541)	2387	(332)	0.529
IL-18 (pg/mL)	220.5	(82.2)	278.6	(111.8)	0.280	214.4	(41.3)	269.2	(89.6)	0.182
IL-1ra (pg/mL)	13.2	(17.9)	8.0	(6.6)	0.617	13.9	(17.0)	26.1	(36.4)	0.699
GSSG:GSH (ratio)	16.6	(6.4)	14.2	(4.6)	0.436	21.7	(10.6)	15.7	(4.8)	0.143
F2-Isoprostane (pg/mL)	43.8	(10.0)	51.7	(17.2)	0.353	45.3	(9.7)	43.3	(10.8)	0.853

^1^ Difference between placebo and treatment in uric acid study based on Mann-Whitney U test

^2^ Difference between placebo and treatment in rasburicase study based on Mann-Whitney U test

### Changes during uric acid and rasburicase treatment on visit 3 day 1 (V3d1)

**Fig 2:**
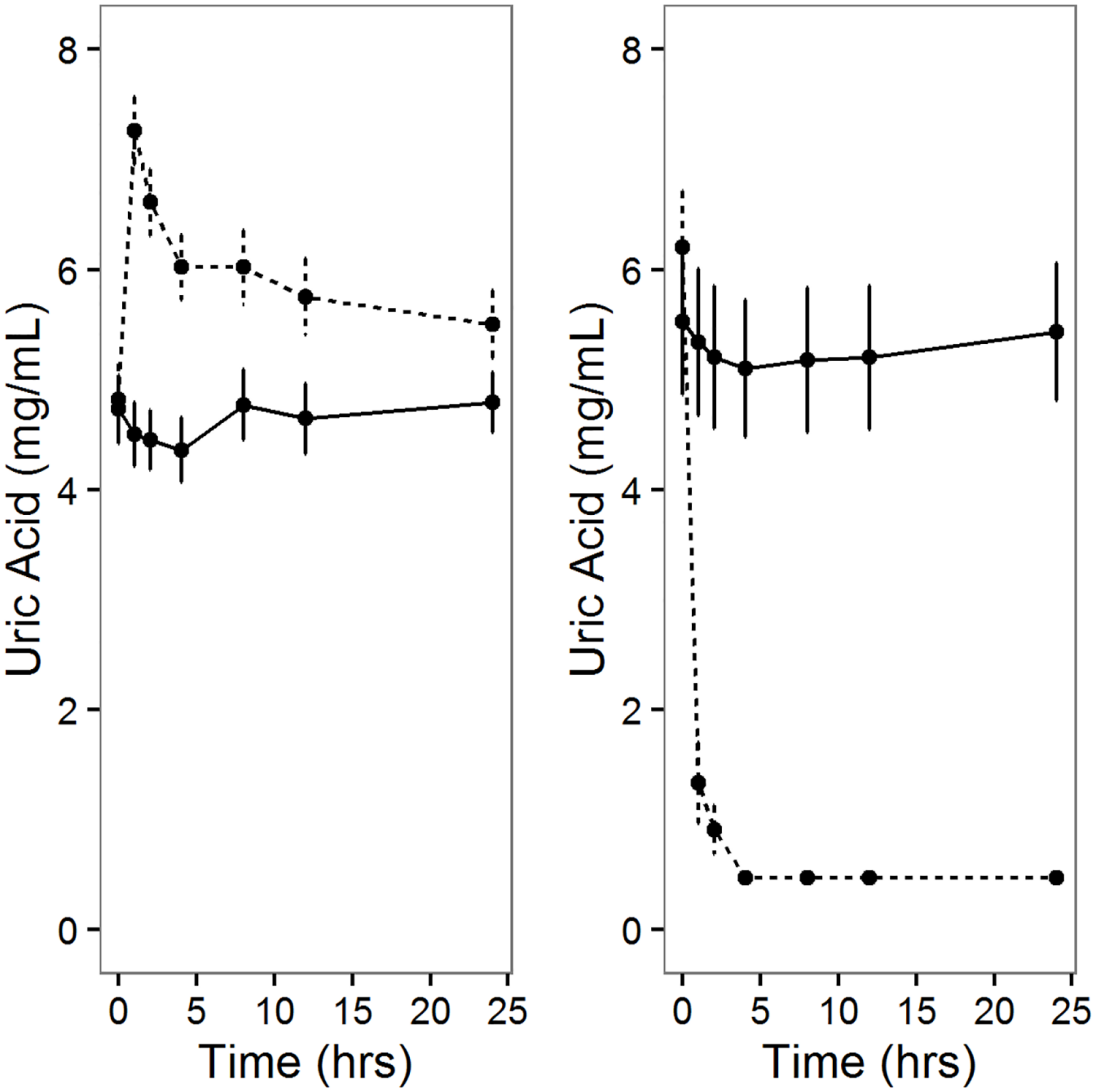
Changes in uric acid levels during uric acid or rasburicase administration. Concentrations of uric acid was measured at 0, 1, 2, 4, 8, 12, and 24 hours following the administration of 500mg uric acid (A) and 0.15 mg/kg rasburicase (B) is displayed. The treatment group is displayed as dotted line (uric acid or rasburicase) and the placebo group as the solid lines. Mean and standard errors are displayed.during uric acid infusion, there was a significant difference in the change in IL-18 (P_treatment*time_ = 0.0006; S2A Fig) and CRP (P_treatment*time_ = 0.045; S3B Fig) over time by treatment. For IL-18 the slope was more negative in the uric acid group compared to placebo group (β_treatment*time12_ = -36.2, P = 0.0002) at the 12^th^ hour. For CRP, at the 8^th^ and 12^th^ hour, there slope for uric acid group was lower than the placebo group (β_treatment*time8_ = -0.14, P = 0.04, β_treatment*time8_ = -0.18, P = 0.007). There were no significant differences in change of other markers by uric acid treatment group.

In the treatment group, we observed a quick rise in uric acid concentrations two hours following the administration of uric acid that persisted for 24 hours (Fig 2A). Conversely, following the administration of rasburicase we observed a rapid drop in uric acid concentration that remained under the threshold of assay detection for 24 hours (Fig 2B). At baseline, in both the uric acid and rasburicase study there were no significant differences in levels of inflammatory cytokines CRP, IL-6, IL-6sr, sGP-130, TNFα, TNFα-RI, TNFαRII, IL-18, and IL1ra as well as oxidative makers ratio of reduced and oxidized glutathione (GSH:GSSG) and F2-isoprostane between treatment and placebo groups (P>0.05).

During the rasburicase infusion, there was a significant difference in the change in sgp130 (P_treatment*time_ = 0.026; S4E Fig) over time by treatment group where the slope was greater at the 4^th^ hour (β_treatment*time4_ = 24.0, P = 0.021) and 12^th^ hour (β_treatment*time4_ = 33.9, P = 0.001). There were no significant differences in change of other markers by rasburicase treatment group.

### Changes in inflammatory marker during oral lipid tolerance tests (OLTT)

The OLTT was conducted before (V2) and after (V3d2) the uric acid or rasburicase administration or respective placebo.

**Fig 3:**
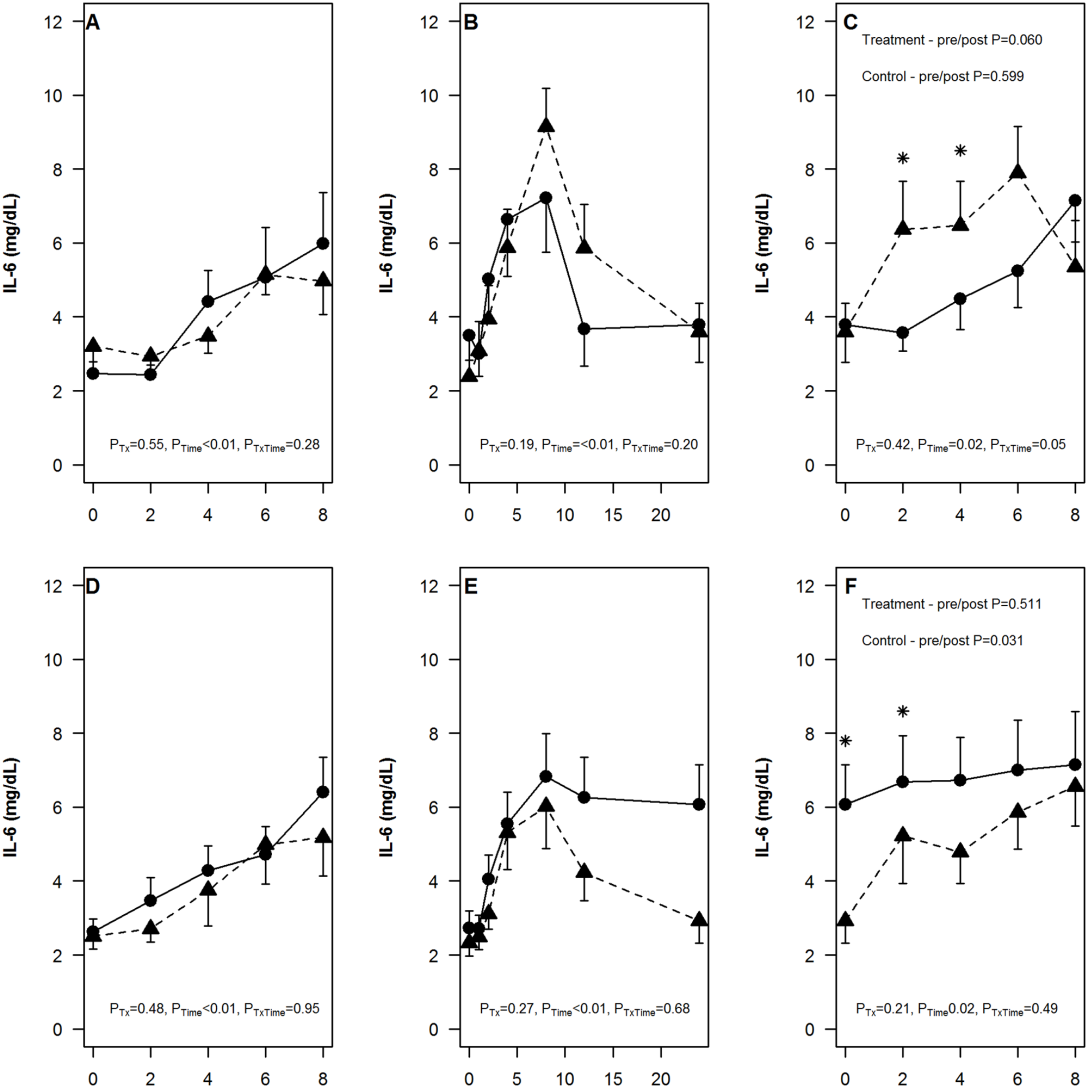
Changes in IL-6 levels during uric acid or rasburicase infusion and oral lipid tolerance test pre- and post- intervention. The level of IL-6 was measured at 0,2,4,6 and 8 hours during the oral lipid tolerance test a day before (A,D) and after (C,F) following the administration of uric acid (A-C) or rasburicase (D-F). During the intervention, IL-6 was measured at 0, 1, 2, 4, 8, 12, and 24* hours after the administration of 500mg of uric acid (B) or 0.15mg/kg of rasburicase (E). The effect of treatment (P_Tx_), time (P_Time_) and slope of change over time by treatment group (P_TxTime_) from the mixed effect model is presented at the bottom of each figure. Differences in the postprandial pattern of IL-6 before and after treatment is displayed (C,F). The treatment group is displayed as triangles and the placebo group as the circles. The mean and standard errors are displayed. *The 24-hour time point after intervention is the baseline, or time 0 of the oral lipid tolerance test conducted the following day.

Notably, consistent with previous studies, there was a consistent rise in postprandial IL-6 for all OLTT tests (P_time_<0.001; Fig 3). In the treatment group, the rise in IL-6 was higher in the OLTT tests performed after the administration of UA compare the OLTT performed at baseline with significant differences at hours 2 and 4 (P<0.05; Fig 3A and 3C). On the other hand, in the placebo group, the rise in IL-6 was similar in the two OLTT performed before and after the infusion date. Interestingly, changes in IL-6 levels were similar in the OLTT tests performed before and after the administration of rasburicase (V2 vs V3d2), while greater postprandial rise in IL-6 was observed after the administration of placebo arm of the rasburicase with significant differences observed at 0 and 2^nd^ hour (Fig 3D and 3F). There were 2 subjects in the rasburicase placebo group that had high levels of IL-6 from baseline and did not show any change throughout the whole OLTT period (S6 Fig). Since IL-6 levels were high from baseline, it possible that these subjects were not fasted before the test. After removing these subjects, the difference in postprandial IL-6 levels on day V2 and V3d2 was no longer significant (S7 Fig).

There was also significant difference in postprandial CRP in the treatment group at V2 and V3d2 for the rasburicase study (P = 0.002, S3A and S3C Fig). This difference was driven by decline in mean CRP levels on the 6^th^ hour on V3d2, however there were no significant differences between mean CRP at this time point between V2 and V3d2.

There were no differences in postprandial changes before and after uric acid or rasburicase treatment for sgp130 (S4 Fig) or IL6sr (S5 Fig).

### Gene expression changes

**Fig 4:**
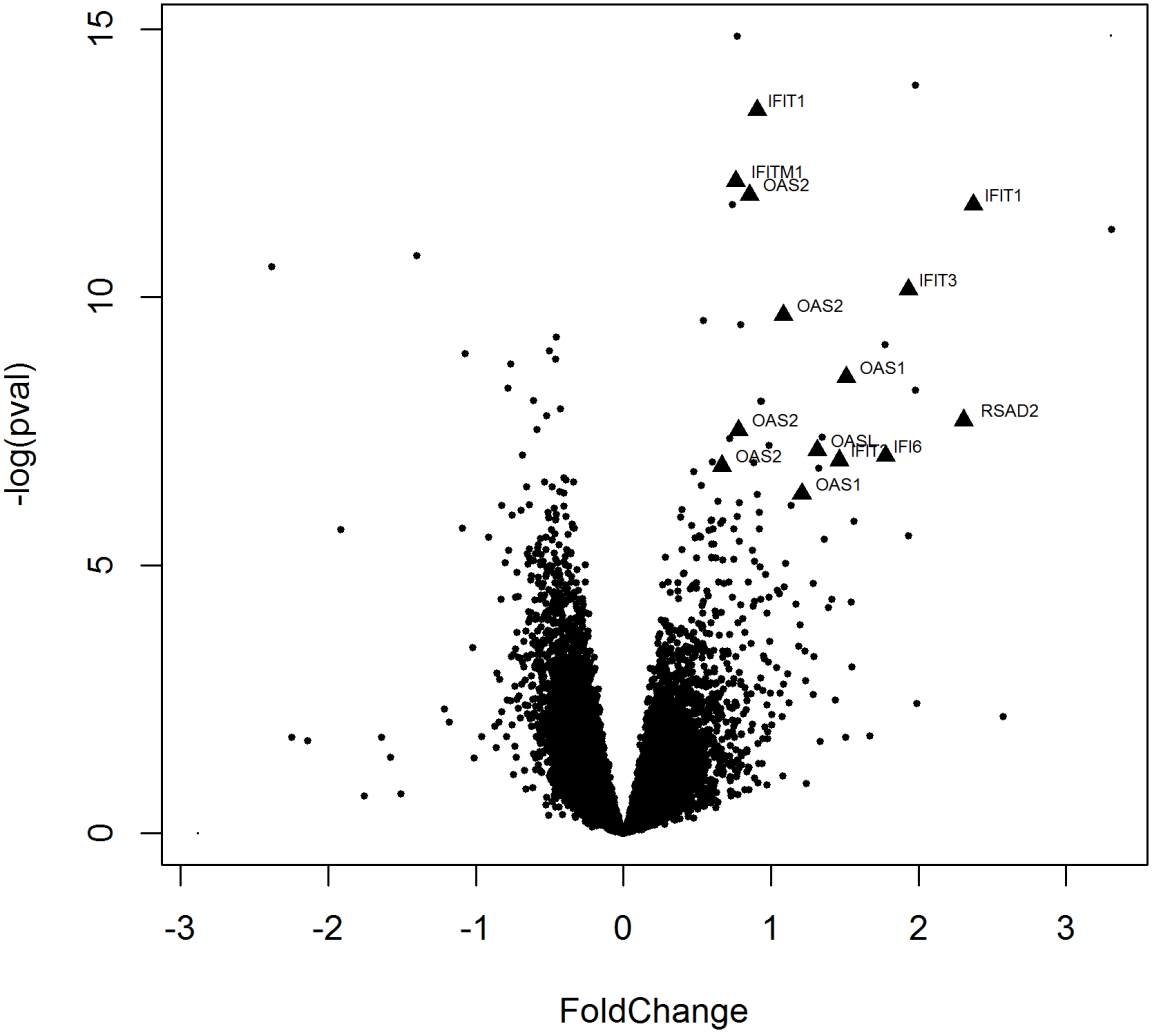
Volcano plot of gene expression changes by rasburicase treatment. The level of gene expression was assessed at baseline and after 12 and 24 hours after infusion of 0.15mg/kg of rasburicase. The difference in gene expression by treatment group is displayed. The genes in the interferon signaling pathway that are significantly differentially expressed are shown (filled triangles).

From the analysis of gene expression profiles over a 24-hr period there were several probes that reached genome-wide significant differences by treatment (S1 and S2 Tables). Most notably, there were three probes with significantly expression by rasburicase infusion that are of particular interest. From the gene enrichment ontology analysis, the interferon signaling pathway was the most significant GO term with 19 genes (IFITM1, RSAD, IFIT2, IFIT1, IFIT3, OAS3, OASL, IFI35, ISG20, GBP2, XAF1, IRF7, MX2, OAS1, OAS2, SP100, IFI6) that were differentially expressed by rasburicase treatment (Fig 4; S2 Table). For all of these genes, there was higher expression in the rasburicase treatment compared to placebo. Surprisingly, we did not observe any differences in IL-6 expression probes in the uric acid or rasburicase study.

## Discussion

In this study we examined the changes in serum inflammatory markers following an acute change in uric acid levels. In addition, we examined whether acute changes in uric acid level affect the inflammatory response induced by a lipid tolerance test, operationalized as rising levels of pro-inflammatory biomarkers. Out of all the inflammatory markers measured in this study, IL-6 was most informative in measuring the postprandial increase in inflammation as indicated in the study rise in serum IL-6 levels following consumption of a fatty meal. This change in IL-6 levels following a various fat load has been previously described [41]. In this study, we observed that there is an accentuated postprandial rise in IL-6 in subjects treated with uric acid while this elevation was not observed in the placebo group. For the rasburicase arm of the study, the rasburicase group did not differ in their postprandial responses despite the acute lowering of uric acid levels. However, contrary to expectations, the placebo group had an elevated postprandial IL-6 level the day after the rasburicase placebo.

During infusion of uric acid and rasburicase, we find no major perturbation in inflammation or oxidative stress as indicated by the biomarkers measured in our study. The gene expression analysis performed during the infusion detected changes in a limited number of genes, some of which are related to immune response pathways. In particular, for the rasburicase study, there were enrichment of genes in immune-related pathway of genes involved in interferon signaling. During the administration of uric acid or rasburicase, such change in gene expression was not reflected into a differential production of pro-inflammatory cytokines in the circulation. However in the uric acid arm of the study, we observed an accentuated postprandial elevation of IL-6 OLTT suggesting that higher uric acid boost the magnitude of the response to an inflammatory stimulus. Such differential response in IL-6 elevation was not paralleled by any meaningful changes in whole blood gene expression.

Our data indicate that uric acid promotes a pro-inflammatory diathesis by tuning up the mechanism that regulates the intensity of the inflammatory response, at least those that modulate the production of soluble inflammatory mediators. The molecular mechanisms that amplify the inflammatory response remain unknown, but some hypotheses can be proposed. There is evidence that high uric acid levels activate the transcription factor NF-kB in several tissues. In the kidney, uric acid induces the rapid activation of NF-kB which is accompanied by translocation to the nucleus [42]. In these cells, NF-kB activation leads to increased expression of chemokines and cytokines such as monocyte Chemoattractant Protein-1 (MCP-1) and TNFα which in turn induces renal infiltration of inflammatory cell. Similarly, in the vascular smooth muscle cells (VSMC), uric acid induces the production of MCP-1 through NF-kB activation [43]. In addition to the NF-kB activation, other important pathways for uric acid mediated inflammation include the extracellular signal-regulated kinase (ERK) mitogen-activated protein kinases (MAPK), cyclooxygenase-2 (COX-2) and platelet-derived growth factor (PDGF) [43]. Association between uric acid and inflammation has previously been reported in humans. In the one study of 957 older Italians free of renal disease or gout, having higher uric acid was associated with increased neutrophils, CRP, IL-1ra, IL-6, IL-18 and TNFα [2]. In this same population, uric acid was predictive of greater increase in CRP a 3-year follow up as well as higher baseline uric acid predicted greater probability of developing clinically relevant increased IL-6 (>2.5pg/ml) and CRP (>3mg/L) [15]. Taken together, these observations are consistent with the increase IL-6 response we observed in the uric acid infused participants and suggest that this increase may be due to activation of pathways including NF-kB. The fact that a pro-inflammatory effect mediated by NF-kB suggested by the literature is not confirmed by changes in gene expression observed in our study is puzzling. We observed differences in genes in the interferon signaling that is regulated by NF-kB [44]. However, this alternative hypothesis was not previously investigated and should be specifically tested in future research.

Three published studies suggest that the administration of uric acid in humans increases antioxidant the serum capacity. In the first study, the administration of 1000mg of uric acid increase in total antioxidant compared to placebo [30]. In the second study, the administration of 500mg of uric acid followed by 20 minutes of high intensity aerobic exercise lead to similar increases in antioxidant capacity and blunting of exercise induced oxidative stress as measures by circulating 8-isoprostaglandin F2α [29]. In the final study, administration of 500 mg of UA restored endothelial-dependent nitric oxide mediated vasodilator response in type I diabetic and smokers but not in healthy volunteers [38]. In smokers and type I diabetics, was restored in response to acetylcholine but not sodium nitroprusside. This suggests that uric acid restores. In our study of middle aged healthy volunteers, we did not measure total antioxidant capacity. However, oxidative stress was assessed through levels of F2-isoprostane levels and the ratio of reduced to oxidized glutathione (GSH:GSSH). There were no differences observed for both markers of oxidative stress in both the uric acid and rasburicase arm of the study suggesting that uric acid itself does not increase or acts as a buffer to oxidative stress.

There have been several studies that examined the effect of lowering uric acid on inflammation with varying results. Most of these studies were conducted on subjects with chronic conditions such as metabolic syndrome, CKD or hypertension therefore the results are difficult to compare with our study. Inflammatory marker CRP is the most consistently studies cytokine and some studies report reduced CRP [45, 46] after lowering uric acid for varying follow up periods while others showed no change [47, 48]. These differences may be due to differences in the diseases that the patients had, or differences in follow up periods. Of note, the studies that report no significant changes in CRP had follow up periods of 2 months or less. Other inflammatory markers that were shown to be reduced with uric acid lowering were sICAM-1 and TNFα [47, 49]. Different from previous reports, our study examined the effect of acute lowering of uric acid in healthy subjects. It is noteworthy that we observed no changes in inflammatory markers within the study period. This would suggest that acute lowering of uric acid does not have any effect on inflammation in healthy humans.

There are several limitations to our study. It is possible that our study was underpowered to detect significant changes in some of the inflammatory markers. We studied the acute effects of fast changes in the concentration of UA and whether more progressive or long term changes cause similar changes in the inflammatory diathesis remains unknown. Based on in vitro observation of rapid molecular changes observed such as with NF-kB, we had hypothesized those inflammatory responses to uric acid could be immediate. However, it is possible that after a few hours the system resets and the acute effects detected in this study wear off. For example, previous studies of allopurinol administration suggest that levels of UA needs to be lowered for some weeks or even months before any effect on inflammation is detectable. Another limitation includes differences in age and BMI between placebo and treatment group for the uric acid study. While the analyses were adjusted for these covariates, we cannot exclude the possibility that the effects observed in these studies are due to these confounders or due to other residual confounding effects.

This study has several important strengths. First, the study sample included healthy adults 50–75, thus the study results can be generalized to this source population. This is a randomized placebo controlled study which allows us to make conclusions about the causal relationship between acute changes in uric acid levels and inflammation. Based on results from large observational studies it was hypothesized that UA is pro-inflammatory. Our study strongly confirms this hypothesis and suggests that UA, rather than be directly pro-inflammatory, amplifies the inflammatory response to other stimuli. This configures a condition of inflammatory diathesis that, over time, may cause a prolonged exposure to inflammatory markers.

In summary, we report that acute increase of uric acid in healthy subjects with normal uric acid levels results in an increase in inflammatory IL-6 response when the system is challenged with an inflammatory load. This increase in inflammatory states may explain the increase risk of developing chronic conditions such as cardiovascular diseases that is observed with increased uric acid in population studies. Our study also suggests that treatment should be considered even for moderately elevated levels of UA. Whether such a treatment can prevent negative health outcomes related to a chronic pro-inflammatory state should be tested in appropriately designed clinical trials.

## Supporting information

S1 Fig Schematic of study protocol. Twenty subjects were recruited to participate in the uric acid or rasburicase intervention study. Eligibility was determined during the prescreen visit. One week following this visit, participants returned for visit two and the initial oral lipid tolerance test (OLTT) was conducted. Two days following this visit, the intervention was carried out over two days (visit 3). One the first day of visit 3, participants were infused with either drug (uric acid or rasburicase) or placebo. Another OLTT was conducted on the second day of visit 3. The final follow-up visit was conducted 2 weeks after visit 3.
(TIF)

S2 Fig Changes in blood cytokine and oxidative stress marker levels during uric acid or rasburicase infusion. The levels of IL-18 (A,B), TNFα (C,D), TNFαR1 (E,F), TNFαR2 (G,H), F2-isoprostane (I, J), IL-1RA (K,L), and the ratio of GSH to GSSH (GSH:GSSH; M,N) were measured at 0, 1, 2, 4, 8, 12, and 24 hours after the administration of 500mg of uric acid (A,C,E,G,I,K,M) or 0.15mg/kg of rasburicase (B,D,F,H,J,L,N). The effect of treatment (P_Tx_), time (P_Time_) and slope of change over time by treatment group (P_TxTime_) from the mixed effect model is presented at the bottom of each figure. The treatment group is displayed as triangles and the placebo group as the circles. The mean and standard errors are displayed.
(PDF)

S3 Fig Changes in CRP levels during uric acid or rasburicase infusion and oral lipid tolerance test pre- and post- intervention. The level of CRP was measured at 0,2,4,6 and 8 hours during the oral lipid tolerance test a day before (A,D) and after (C,F) following the administration of uric acid (A-C) or rasburicase (D-F). During the intervention, CRP was measured at 0, 1, 2, 4, 8, 12, and 24* hours after the administration of 500mg of uric acid (B) or 0.15mg/kg of rasburicase (E). The effect of treatment (P_Tx_), time (P_Time_) and slope of change over time by treatment group (P_TxTime_) from the mixed effect model is presented at the bottom of each figure. Differences in the postprandial pattern of CRP before and after treatment is displayed (C,F). The treatment group is displayed as triangles and the placebo group as the circles. The mean and standard errors are displayed. *The 24-hour time point after intervention is the baseline, or time 0 of the oral lipid tolerance test conducted the following day.
(PDF)

S4 Fig Changes in sGP-130 levels during uric acid or rasburicase infusion and oral lipid tolerance test pre- and post- intervention. The level of sGP-130 was measured at 0,2,4,6 and 8 hours during the oral lipid tolerance test a day before (A,D) and after (C,F) following the administration of uric acid (A-C) or rasburicase (D-F). During the intervention, sGP-130 was measured at 0, 1, 2, 4, 8, 12, and 24* hours after the administration of 500mg of uric acid (B) or 0.15mg/kg of rasburicase (E). The effect of treatment (P_Tx_), time (P_Time_) and slope of change over time by treatment group (P_TxTime_) from the mixed effect model is presented at the bottom of each figure. Differences in the postprandial pattern of sGP-130 before and after treatment is displayed (C,F). The treatment group is displayed as triangles and the placebo group as the circles. The mean and standard errors are displayed. *The 24-hour time point after intervention is the baseline, or time 0 of the oral lipid tolerance test conducted the following day.
(PDF)

S5 Fig Changes in IL-6sr levels during uric acid or rasburicase infusion and oral lipid tolerance test pre- and post- intervention. The level of IL-6sr was measured at 0,2,4,6 and 8 hours during the oral lipid tolerance test a day before (A,D) and after (C,F) following the administration of uric acid (A-C) or rasburicase (D-F). During the intervention, IL-6sr was measured at 0, 1, 2, 4, 8, 12, and 24* hours after the administration of 500mg of uric acid (B) or 0.15mg/kg of rasburicase (E). The effect of treatment (P_Tx_), time (P_Time_) and slope of change over time by treatment group (P_TxTime_) from the mixed effect model is presented at the bottom of each figure. Differences in the postprandial pattern of IL-6srbefore and after treatment is displayed (C,F). The treatment group is displayed as triangles and the placebo group as the circles. The mean and standard errors are displayed. *The 24-hour time point after intervention is the baseline, or time 0 of the oral lipid tolerance test conducted the following day.
(PDF)

S6 Fig Individuals trajectory of IL-6 concentrations during the oral lipid tolerance test on V3d2 in the rasburicase study. To determine potential outliers in IL-6 concentration during the oral lipid tolerance test following rasburicase administration, we examined individual IL-6 levels. Two subjects had consistently high IL-6 levels starting at baseline despite being fasted (left panel solid lines).
(PDF)

S7 Fig IL-6 levels during oral lipid tolerance on V3d2 after removal of outliers in the rasburicase study. In the rasburicase study, two outliers with consistently high IL-6 levels from baseline of the post intervention oral lipid tolerance test were identified. The figure represents the postprandial changes in IL-6 during the oral lipid tolerance test after removing these two outliers.
(PDF)

S1 Table Expression probes differentially expressed by treatment during uric acid administration. (DOCX)

S2 Table Expression probes differentially expressed by treatment during rasburicase administration. (DOCX)

S3 Table Mean levels of inflammatory markers (IL-6, IL-6sr, sgp-130, CRP) during oral lipid tolerance test before uric acid or rasburicase administration. (DOCX)

S4 Table Mean levels of inflammatory markers (IL-6, IL-6sr, sgp-130, CRP) during uric acid or rasburicase administration. (DOCX)

S5 Table Mean levels of inflammatory markers (IL-6, IL-6sr, sgp-130, CRP) during oral lipid tolerance test after uric acid or rasburicase administration. (DOCX)

S1 File CONSORT checklist. (DOCX)

S2 File Study protocol. (DOC)
