# Supplementary figures and images for: A double blind placebo controlled randomized trial of the effect of acute uric acid changes on inflammatory markers in humans: A pilot study
Source: PLoS One. 2017 Aug 7;12(8):e0181100. doi: 10.1371/journal.pone.0181100 (PMC5546625; doi:10.1371/journal.pone.0181100)

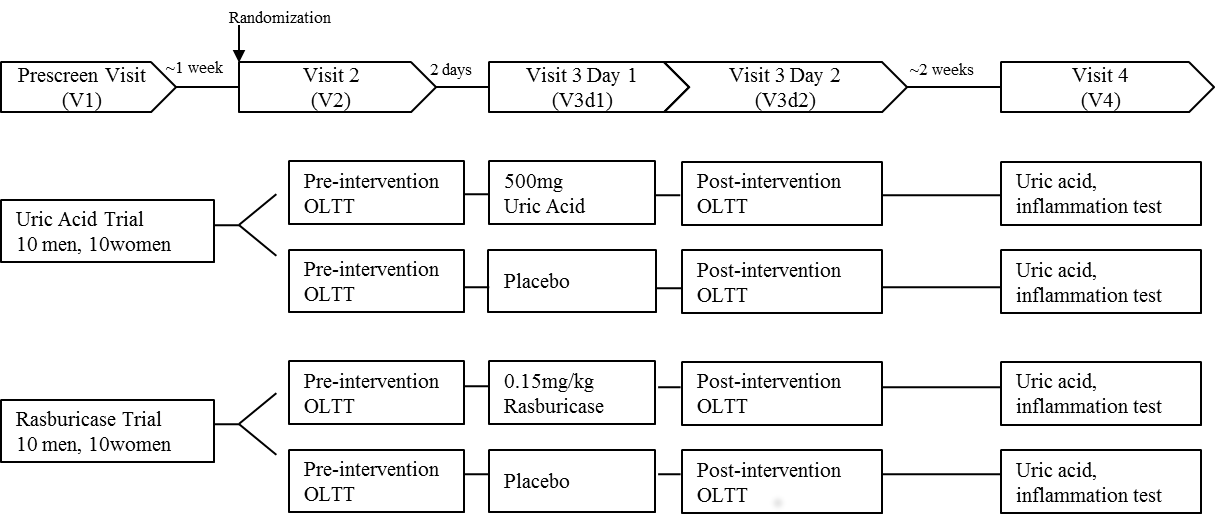

Supplement: S1 Fig — Twenty subjects were recruited to participate in the uric acid or rasburicase intervention study. Eligibility was determined during the prescreen visit. One week following this visit, participants returned for visit two and the initial oral lipid tolerance test (OLTT) was conducted. Two days following this visit, the intervention was carried out over two days (visit 3). One the first day of visit 3, participants were infused with either drug (uric acid or rasburicase) or placebo. Another OLTT was conducted on the second day of visit 3. The final follow-up visit was conducted 2 weeks after visit 3. (TIF) [file pone.0181100.s001.tif]

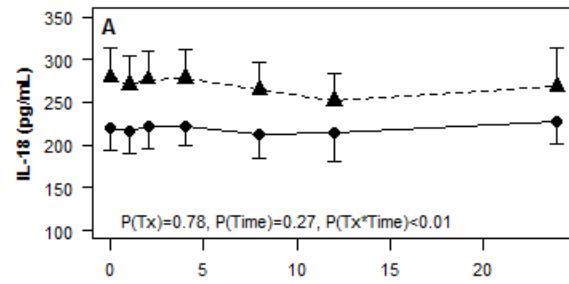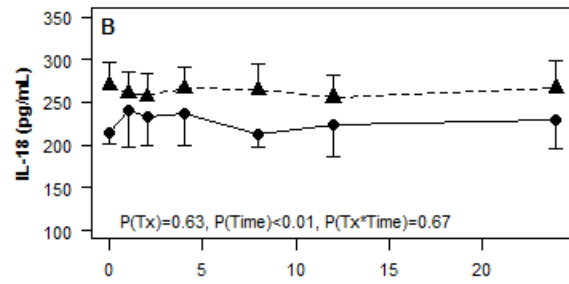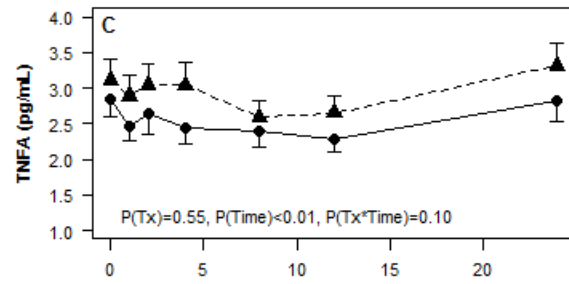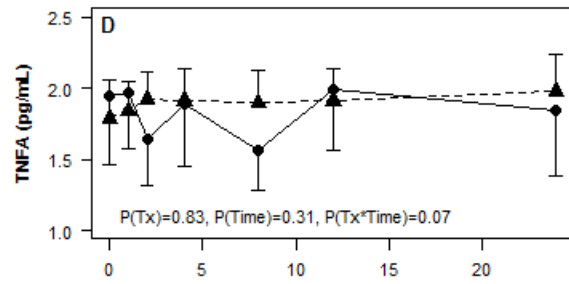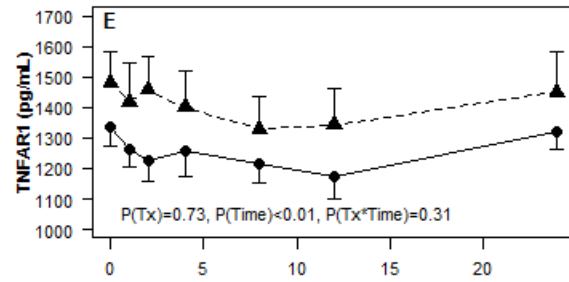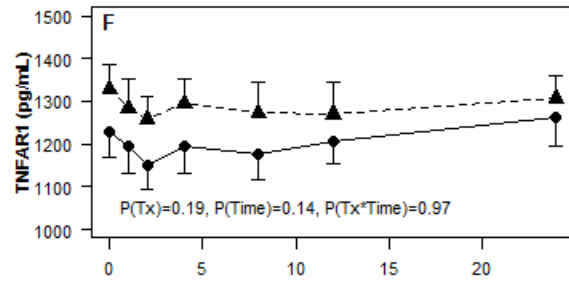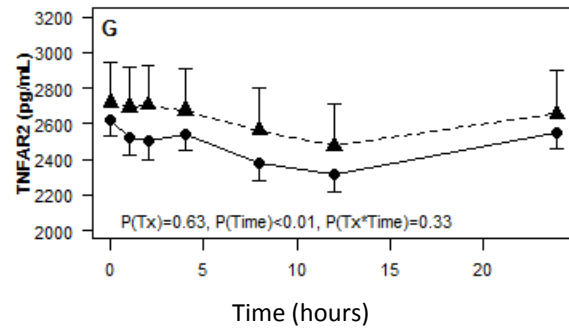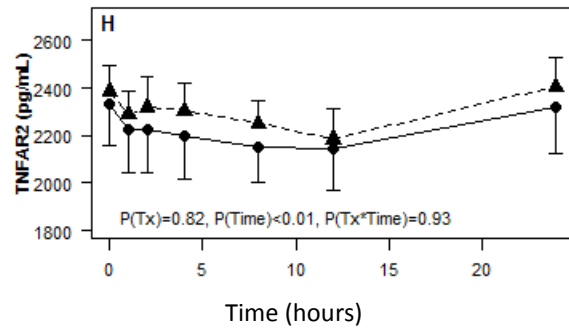

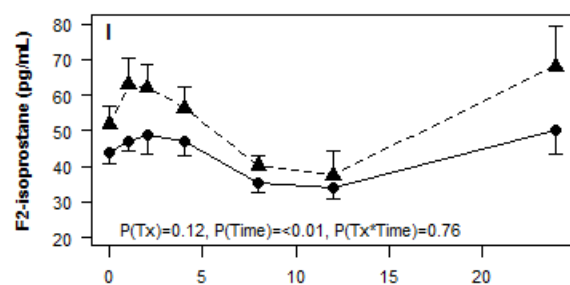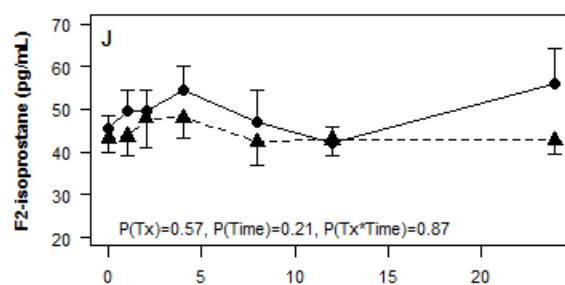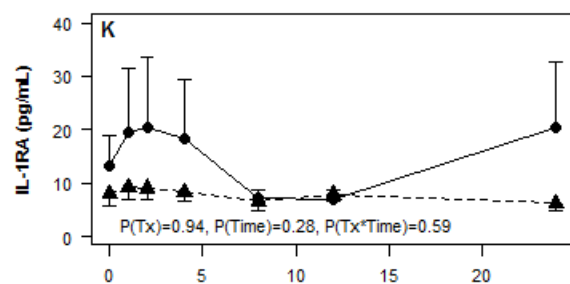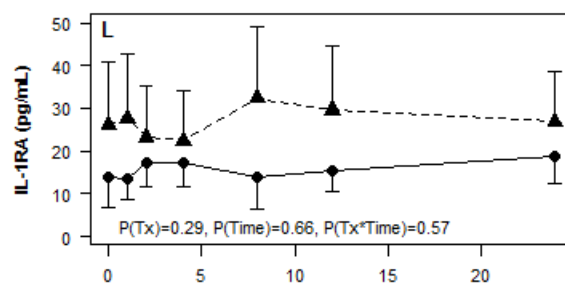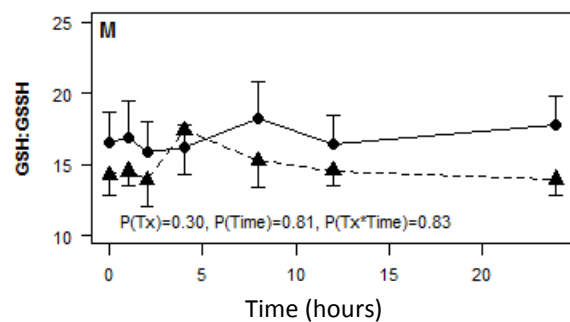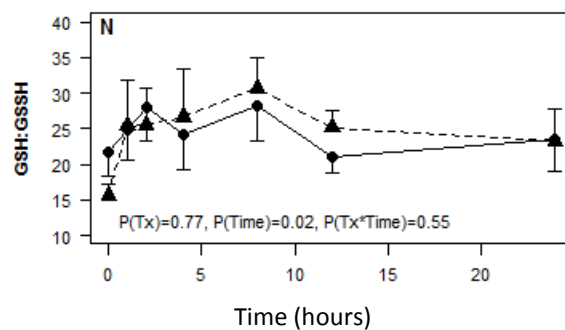

Supplement: S2 Fig — The levels of IL-18 (A,B), TNFα (C,D), TNFαR1 (E,F), TNFαR2 (G,H), F2-isoprostane (I, J), IL-1RA (K,L), and the ratio of GSH to GSSH (GSH:GSSH; M,N) were measured at 0, 1, 2, 4, 8, 12, and 24 hours after the administration of 500mg of uric acid (A,C,E,G,I,K,M) or 0.15mg/kg of rasburicase (B,D,F,H,J,L,N). The effect of treatment (PTx), time (PTime) and slope of change over time by treatment group (PTxTime) from the mixed effect model is presented at the bottom of each figure. The treatment group is displayed as triangles and the placebo group as the circles. The mean and standard errors are displayed. (PDF) [file pone.0181100.s002.pdf]

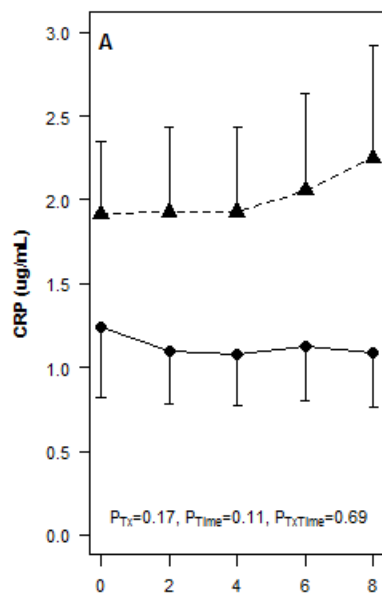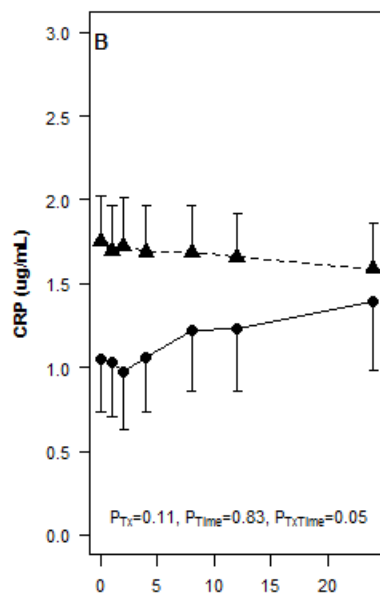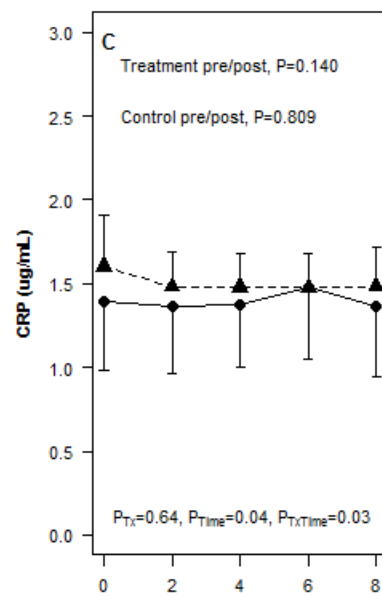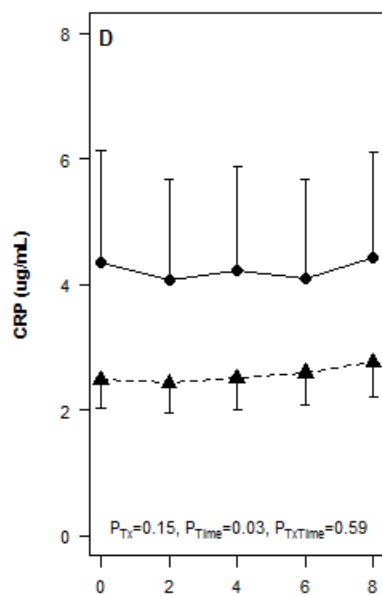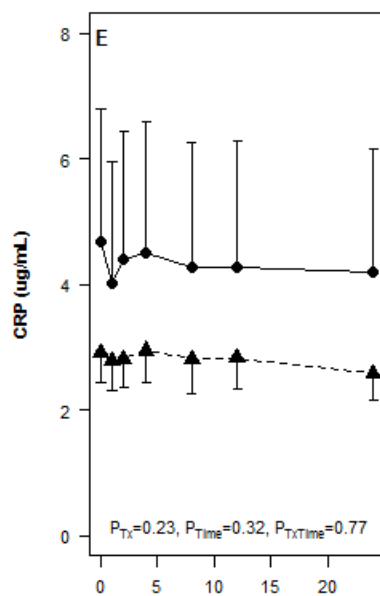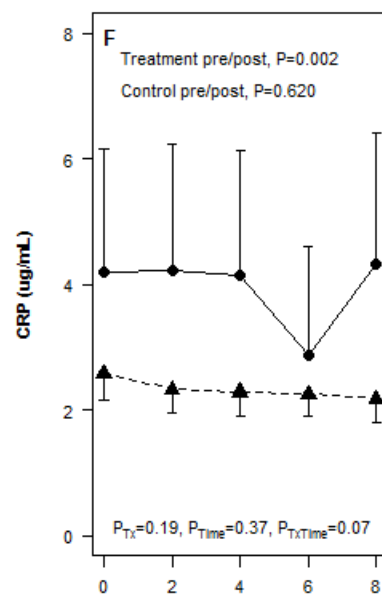

Time (hours)

Supplement: S3 Fig — The level of CRP was measured at 0,2,4,6 and 8 hours during the oral lipid tolerance test a day before (A,D) and after (C,F) following the administration of uric acid (A-C) or rasburicase (D-F). During the intervention, CRP was measured at 0, 1, 2, 4, 8, 12, and 24* hours after the administration of 500mg of uric acid (B) or 0.15mg/kg of rasburicase (E). The effect of treatment (PTx), time (PTime) and slope of change over time by treatment group (PTxTime) from the mixed effect model is presented at the bottom of each figure. Differences in the postprandial pattern of CRP before and after treatment is displayed (C,F). The treatment group is displayed as triangles and the placebo group as the circles. The mean and standard errors are displayed. *The 24-hour time point after intervention is the baseline, or time 0 of the oral lipid tolerance test conducted the following day. (PDF) [file pone.0181100.s003.pdf]

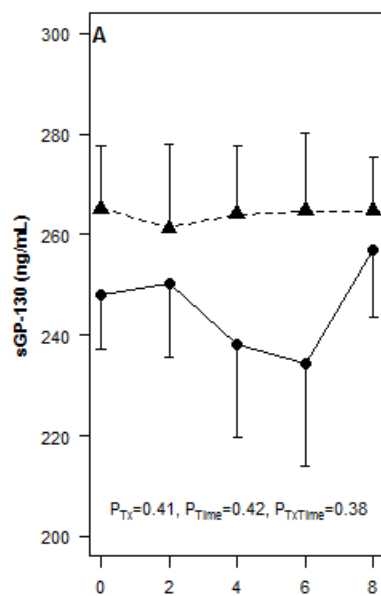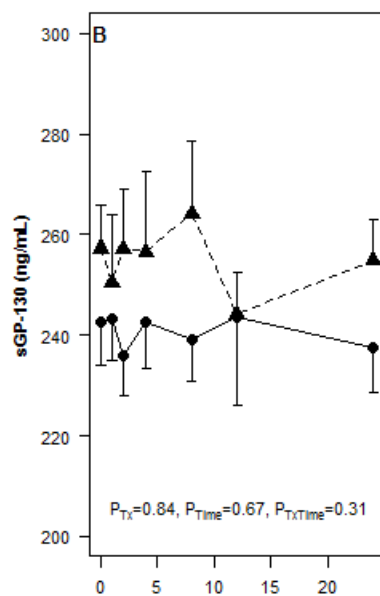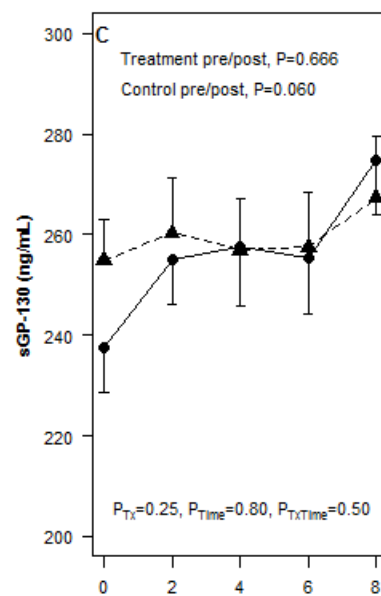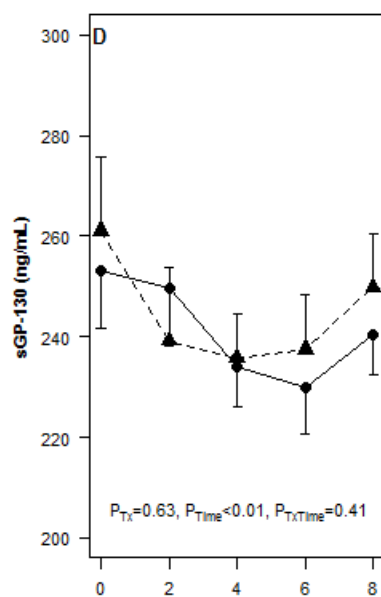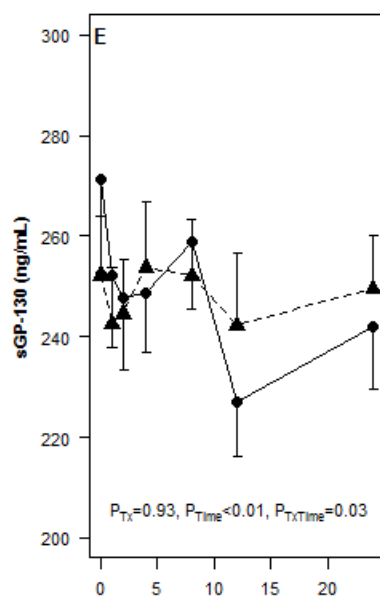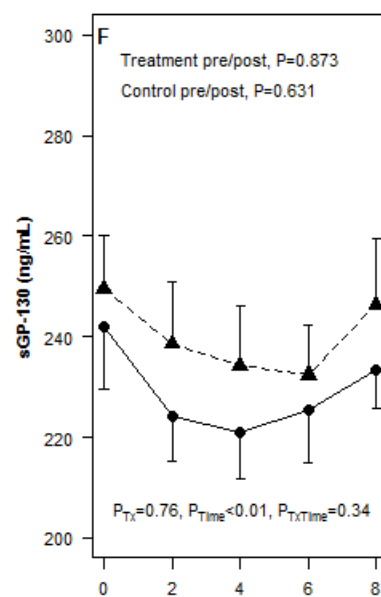

Time (hours)

Supplement: S4 Fig — The level of sGP-130 was measured at 0,2,4,6 and 8 hours during the oral lipid tolerance test a day before (A,D) and after (C,F) following the administration of uric acid (A-C) or rasburicase (D-F). During the intervention, sGP-130 was measured at 0, 1, 2, 4, 8, 12, and 24* hours after the administration of 500mg of uric acid (B) or 0.15mg/kg of rasburicase (E). The effect of treatment (PTx), time (PTime) and slope of change over time by treatment group (PTxTime) from the mixed effect model is presented at the bottom of each figure. Differences in the postprandial pattern of sGP-130 before and after treatment is displayed (C,F). The treatment group is displayed as triangles and the placebo group as the circles. The mean and standard errors are displayed. *The 24-hour time point after intervention is the baseline, or time 0 of the oral lipid tolerance test conducted the following day. (PDF) [file pone.0181100.s004.pdf]

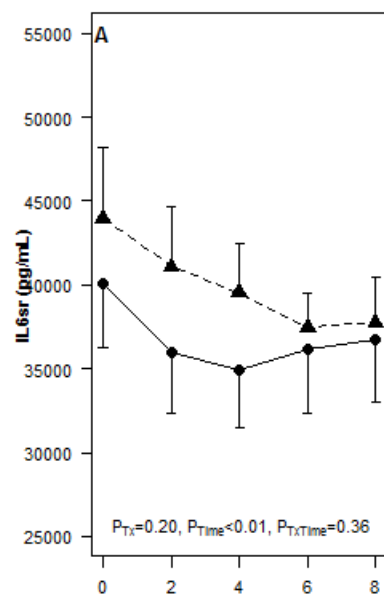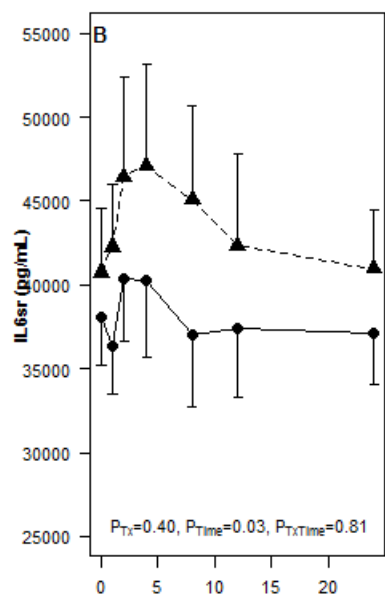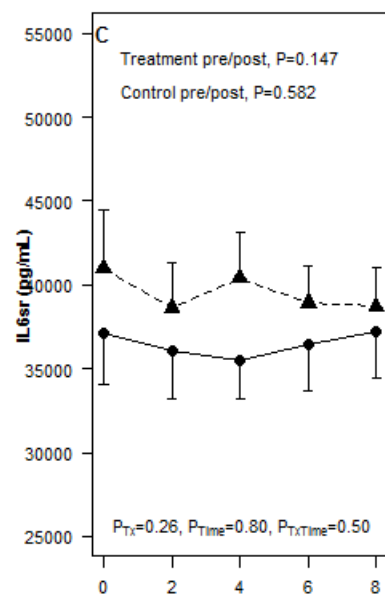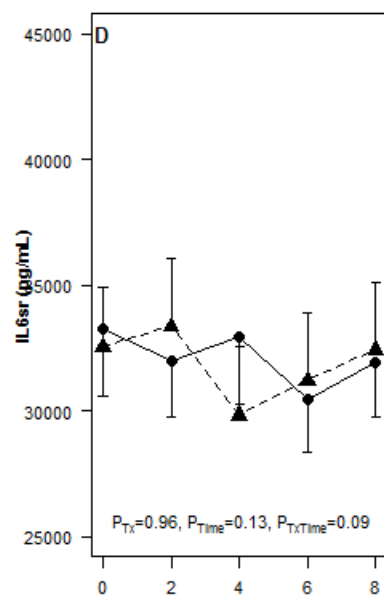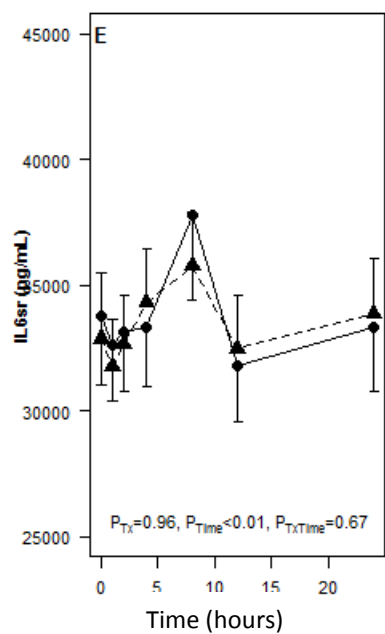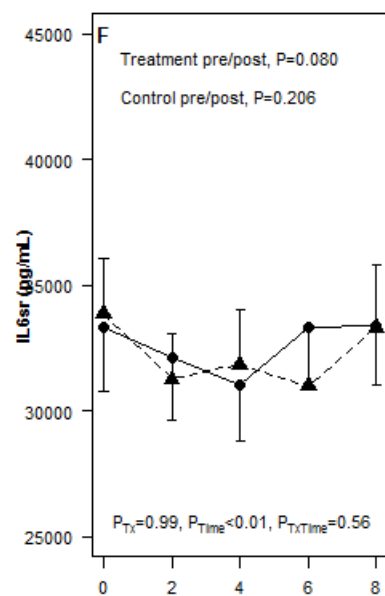

Supplement: S5 Fig — The level of IL-6sr was measured at 0,2,4,6 and 8 hours during the oral lipid tolerance test a day before (A,D) and after (C,F) following the administration of uric acid (A-C) or rasburicase (D-F). During the intervention, IL-6sr was measured at 0, 1, 2, 4, 8, 12, and 24* hours after the administration of 500mg of uric acid (B) or 0.15mg/kg of rasburicase (E). The effect of treatment (PTx), time (PTime) and slope of change over time by treatment group (PTxTime) from the mixed effect model is presented at the bottom of each figure. Differences in the postprandial pattern of IL-6srbefore and after treatment is displayed (C,F). The treatment group is displayed as triangles and the placebo group as the circles. The mean and standard errors are displayed. *The 24-hour time point after intervention is the baseline, or time 0 of the oral lipid tolerance test conducted the following day. (PDF) [file pone.0181100.s005.pdf]

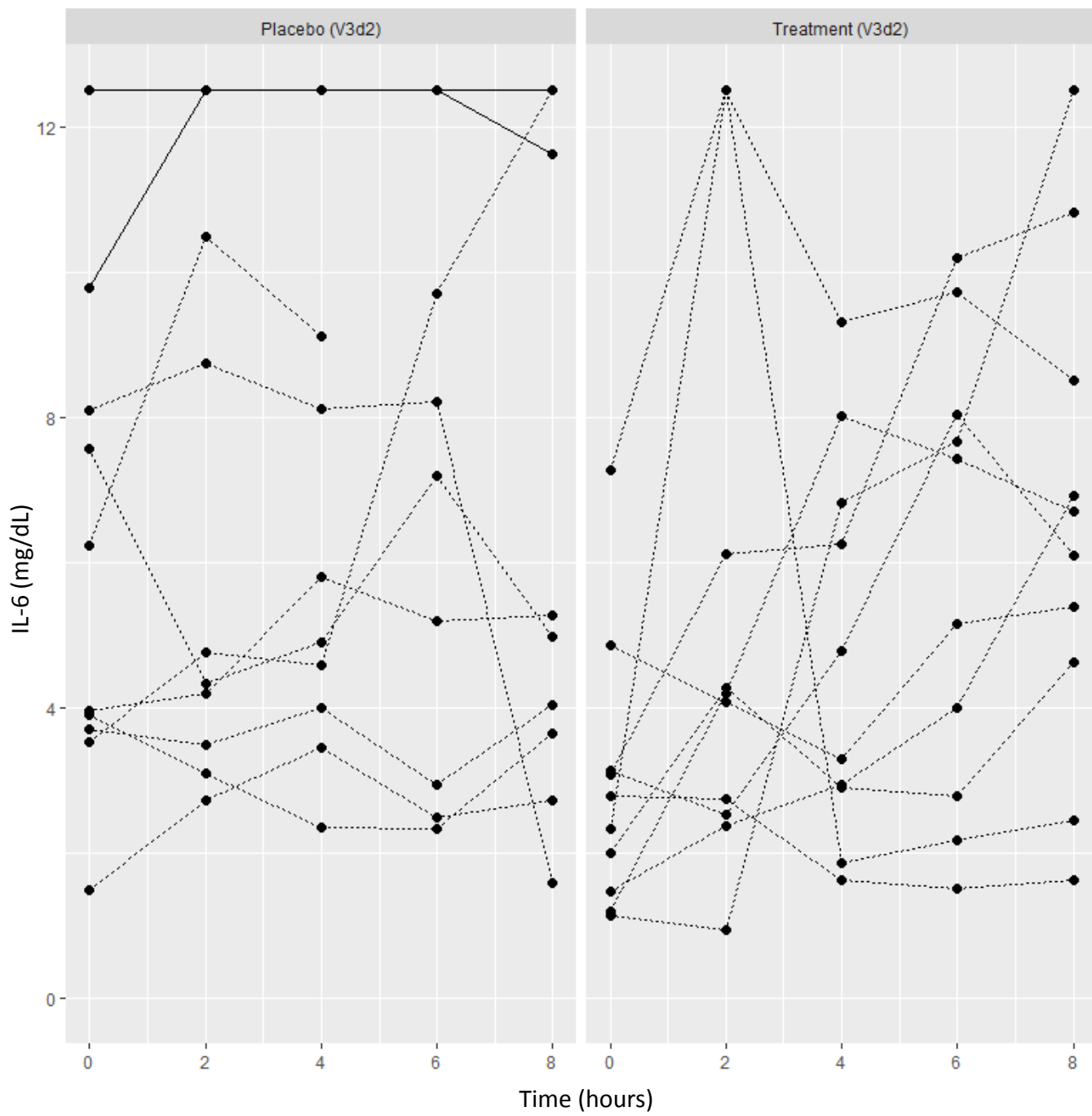

Supplement: S6 Fig — To determine potential outliers in IL-6 concentration during the oral lipid tolerance test following rasburicase administration, we examined individual IL-6 levels. Two subjects had consistently high IL-6 levels starting at baseline despite being fasted (left panel solid lines). (PDF) [file pone.0181100.s006.pdf]

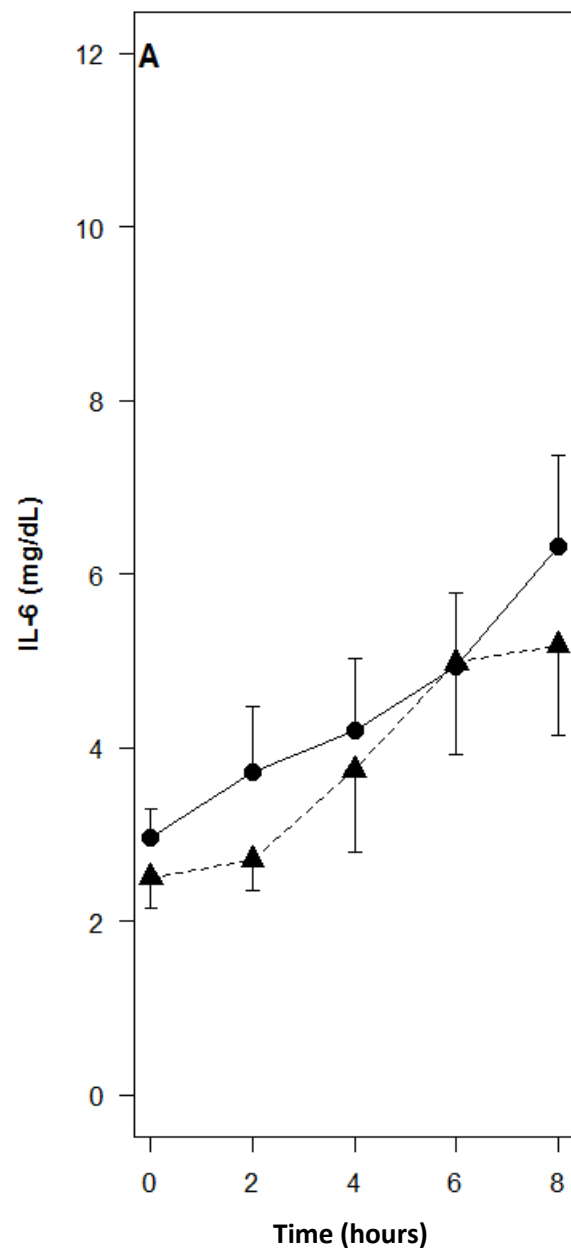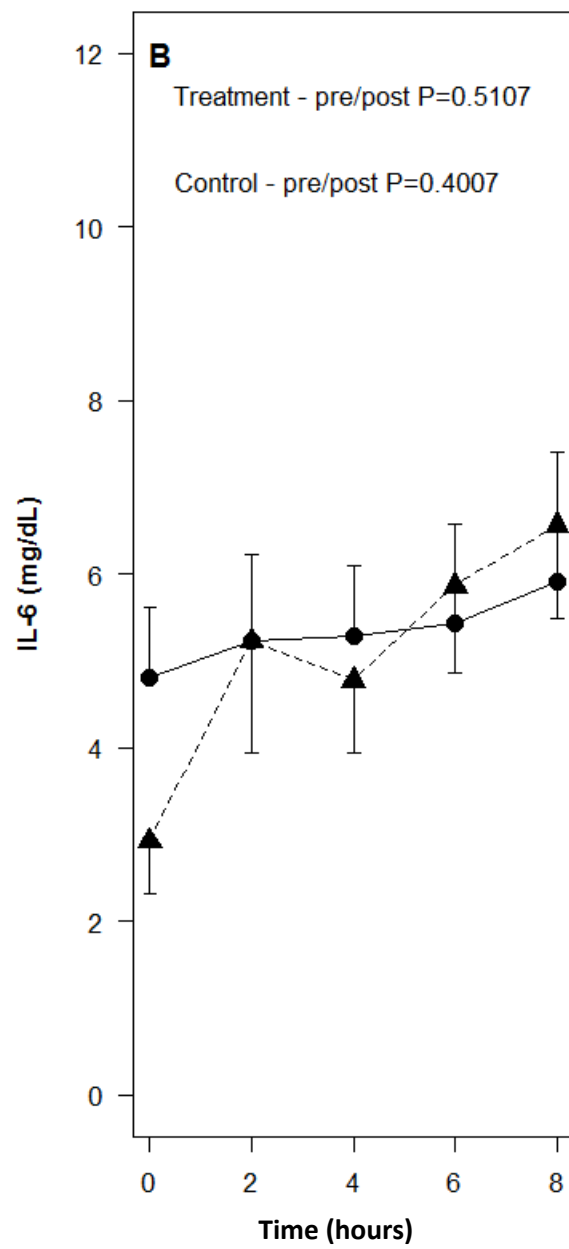

Supplement: S7 Fig — In the rasburicase study, two outliers with consistently high IL-6 levels from baseline of the post intervention oral lipid tolerance test were identified. The figure represents the postprandial changes in IL-6 during the oral lipid tolerance test after removing these two outliers. (PDF) [file pone.0181100.s007.pdf]
