# Supplementary material for: A double blind placebo controlled randomized trial of the effect of acute uric acid changes on inflammatory markers in humans: A pilot study
Source: PLoS One. 2017 Aug 7;12(8):e0181100. doi: 10.1371/journal.pone.0181100 (PMC5546625; doi:10.1371/journal.pone.0181100)
Supplement: S1 Table — (DOCX) [file pone.0181100.s008.docx]

| Markername | Beta | SE | Pvalue | Symbol |
| --- | --- | --- | --- | --- |
| 6520593 | 0.877183886 | 0.113044576 | 8.44E-15 | RNF182 |
| 5820634 | -0.409498676 | 0.069121088 | 3.14E-09 | LOC441150 |
| 1090072 | -0.254695388 | 0.045946237 | 2.97E-08 | KIAA0494 |
| 1230047 | 0.475258827 | 0.086058265 | 3.34E-08 | CBS |
| 7320246 | 0.770250997 | 0.139597928 | 3.44E-08 | HEMGN |
| 3780079 | -0.322052635 | 0.059733278 | 6.99E-08 | LOC100132901 |
| 110653 | 0.642020743 | 0.119763792 | 8.29E-08 | NPL |
| 1240152 | -0.981103127 | 0.183281465 | 8.65E-08 | CFD |
| 3610348 | 0.342397459 | 0.064291532 | 1.01E-07 | FAM10A4 |
| 7610014 | 0.198375501 | 0.038004647 | 1.79E-07 | FAM161A |
| 5360025 | -0.226695413 | 0.043446565 | 1.81E-07 | ST8SIA1 |
| 650168 | -0.3286381 | 0.063483986 | 2.26E-07 | C17orf58 |
| 7610202 | -0.258584185 | 0.050258914 | 2.67E-07 | C1orf66 |
| 6350239 | -0.41451491 | 0.081102532 | 3.20E-07 | RRAS2 |
| 4290088 | 0.254038487 | 0.049907808 | 3.58E-07 | MSRB3 |
| 4150048 | -0.422817696 | 0.083423519 | 4.01E-07 | FABP5 |
| 430356 | 0.903875278 | 0.178956791 | 4.40E-07 | OR2W3 |
| 2340059 | -0.312683926 | 0.062431637 | 5.49E-07 | C16orf53 |

**Table S1.** Top expression probes differentially expressed by treatment during uric acid infusion.
