# Supplementary material for: A double blind placebo controlled randomized trial of the effect of acute uric acid changes on inflammatory markers in humans: A pilot study
Source: PLoS One. 2017 Aug 7;12(8):e0181100. doi: 10.1371/journal.pone.0181100 (PMC5546625; doi:10.1371/journal.pone.0181100)
Supplement: S2 Table — (DOCX) [file pone.0181100.s009.docx]

**Table S1. Top expression probes differentially expressed by treatment during rasburicase infusion.**

| Markername | beta | SE | P | Symbol |
| --- | --- | --- | --- | --- |
| 270240 | 0.772665473 | 0.096619606 | 1.33E-15 | SLC26A8 |
| 5720482 | 1.977976491 | 0.256058132 | 1.11E-14 | HERC5 |
| 2690435 | 0.906080686 | 0.119417532 | 3.26E-14 | IFIT1 |
| 5360156 | 0.761804914 | 0.106083899 | 6.91E-13 | IFITM1 |
| 2230204 | 0.85483437 | 0.120367411 | 1.23E-12 | OAS2 |
| 4760703 | 0.739028992 | 0.104875503 | 1.83E-12 | DDX58 |
| 2000148 | 2.372172454 | 0.336815227 | 1.88E-12 | IFIT1 |
| 1510017 | 3.305592921 | 0.479352277 | 5.35E-12 | LOC388588 |
| 1470102 | -1.402820404 | 0.208344547 | 1.66E-11 | BTNL3 |
| 780403 | -2.382225801 | 0.357513851 | 2.68E-11 | HLA-DQA1 |
| 6510170 | 1.93107246 | 0.296415068 | 7.28E-11 | IFIT3 |
| 7320561 | 1.084377079 | 0.170800532 | 2.17E-10 | OAS2 |
| 5310471 | 0.541744559 | 0.085767586 | 2.68E-10 | UBE2C |
| 630470 | 0.793769718 | 0.12626006 | 3.24E-10 | C19orf33 |
| 2000132 | -0.453401995 | 0.073094703 | 5.54E-10 | LRRC47 |
| 2600747 | 1.77008328 | 0.287793901 | 7.72E-10 | IFIT2 |
| 3710253 | -0.502032934 | 0.082191121 | 1.01E-09 | CAPN12 |
| 5340246 | -1.074146344 | 0.176324797 | 1.12E-09 | CRIP2 |
| 6650452 | -0.460542117 | 0.076111577 | 1.44E-09 | DET1 |
| 6270615 | -0.764780039 | 0.127026527 | 1.74E-09 | GSDMB |
| 1090390 | 1.509503013 | 0.254778692 | 3.13E-09 | OAS1 |
| 50327 | -0.783034745 | 0.133873526 | 4.94E-09 | ZNF589 |
| 2570300 | 1.979319376 | 0.339208639 | 5.38E-09 | IFI44 |
| 5890470 | -0.611284192 | 0.106143128 | 8.46E-09 | CCR6 |
| 3990010 | 0.932091815 | 0.161968645 | 8.68E-09 | NA |
| 5700735 | 0.933073068 | 0.16219665 | 8.78E-09 | PARP9 |
| 2030243 | -0.425388322 | 0.074651461 | 1.21E-08 | FTSJ2 |
| 6110561 | -0.519224169 | 0.091938424 | 1.63E-08 | MRPS27 |
| 3360343 | 2.304882713 | 0.410618187 | 1.99E-08 | RSAD2 |
| 5390494 | -0.585429131 | 0.105581605 | 2.94E-08 | EIF4B |
| 240722 | 0.781141766 | 0.14097993 | 3.01E-08 | OAS2 |
| 2030309 | 1.348537655 | 0.245591478 | 4.00E-08 | SERPING1 |
| 6180056 | 0.718325957 | 0.13118492 | 4.36E-08 | TOR1B |
| 5960343 | 0.985170766 | 0.181556278 | 5.75E-08 | PRIC285 |
| 6280543 | 1.313282588 | 0.243786893 | 7.16E-08 | OASL |
| 2370064 | -0.686057953 | 0.128247172 | 8.82E-08 | ASGR1 |
| 5090215 | 1.776229708 | 0.332487 | 9.18E-08 | IFI6 |
| 520408 | 1.464620303 | 0.27589765 | 1.10E-07 | IFIT3 |
| 6860482 | 0.601874523 | 0.113584282 | 1.16E-07 | HERC6 |
| 5310411 | 0.882048092 | 0.166627161 | 1.20E-07 | H2AFJ |
| 1570156 | 0.669016344 | 0.127079563 | 1.41E-07 | OAS2 |
| 4890270 | 1.324604072 | 0.252270733 | 1.52E-07 | LY6E |
| 3850603 | 0.476974367 | 0.091337162 | 1.77E-07 | PTPRN |
| 4010338 | -0.406055464 | 0.078540103 | 2.34E-07 | RABIF |
| 1850059 | -0.389671252 | 0.075616645 | 2.56E-07 | FLJ42627 |
| 3370372 | -0.340160957 | 0.06623413 | 2.81E-07 | C3orf18 |
| 870437 | -0.535284272 | 0.104231357 | 2.81E-07 | TRPM4 |
| 1580681 | 0.526196489 | 0.102978475 | 3.23E-07 | MS4A3 |
| 5900286 | -0.656073962 | 0.128672513 | 3.42E-07 | ZFP90 |
| 6450154 | -0.481396272 | 0.094453552 | 3.46E-07 | LSS |
| 5810440 | -0.43322056 | 0.085607727 | 4.18E-07 | HNRNPAB |
| 2070687 | -0.405728639 | 0.080370077 | 4.46E-07 | SLC38A1 |
| 7040035 | 1.209017194 | 0.239887784 | 4.66E-07 | OAS1 |
| 2940017 | 0.906354816 | 0.179974378 | 4.75E-07 | SNN |
| 5860551 | 0.641707556 | 0.128941536 | 6.47E-07 | BTNL8 |
| 7610053 | 0.783421625 | 0.157752219 | 6.83E-07 | DDX60 |
| 3400301 | -0.637297641 | 0.128655754 | 7.29E-07 | NA |
| 5260070 | 1.137738075 | 0.230105253 | 7.64E-07 | HES4 |
| 6020523 | -0.824708567 | 0.166836126 | 7.68E-07 | PPP2R2B |
| 6250390 | -0.404369974 | 0.081868401 | 7.84E-07 | TSR2 |
| 6620392 | -0.468526759 | 0.095226542 | 8.65E-07 | LFNG |
| 380561 | 0.396960614 | 0.080877162 | 9.19E-07 | NEBL |
| 270026 | -0.693898718 | 0.141441147 | 9.30E-07 | RASSF1 |
