# Supplementary material for: A double blind placebo controlled randomized trial of the effect of acute uric acid changes on inflammatory markers in humans: A pilot study
Source: PLoS One. 2017 Aug 7;12(8):e0181100. doi: 10.1371/journal.pone.0181100 (PMC5546625; doi:10.1371/journal.pone.0181100)
Supplement: S3 Table — (DOCX) [file pone.0181100.s010.docx]

**S3 Table. Mean levels of inflammatory markers (IL-6, IL-6sr, sgp-130, CRP) during oral lipid tolerance test before uric acid or rasburicase administration.**

| Before Treatment Lipid Tolerance Test | | | | | | | | | | | |
| --- | --- | --- | --- | --- | --- | --- | --- | --- | --- | --- | --- |
|  |  | Placebo | | Uric Acid | |  | Placebo | | Rasburicase | |  |
| Marker | Time Point (hrs) | mean | sd | mean | sd |  | mean | sd | mean | sd |  |
| IL-6 | 0 | 2.5 | 0.9 | 3.2 | 2.5 |  | 2.6 | 1.1 | 2.5 | 1.1 |  |
|  | 2 | 2.4 | 0.8 | 2.9 | 1.7 |  | 3.5 | 2.0 | 2.7 | 1.1 |  |
|  | 4 | 4.4 | 2.4 | 3.5 | 1.5 |  | 4.3 | 2.1 | 3.8 | 3.0 |  |
|  | 6 | 5.1 | 3.8 | 5.1 | 1.7 |  | 4.7 | 2.2 | 5.0 | 3.2 |  |
|  | 8 | 6.0 | 3.7 | 5.0 | 2.7 |  | 6.4 | 2.8 | 5.2 | 3.1 |  |
| IL-6sr | 0 | 40097.6 | 10920.5 | 43962.5 | 13269.8 |  | 33261.8 | 8514.5 | 32557.1 | 7407.0 |  |
|  | 2 | 36020.0 | 11085.3 | 41105.0 | 11136.2 |  | 32001.3 | 7084.4 | 33391.2 | 8495.0 |  |
|  | 4 | 34899.9 | 10112.5 | 39532.4 | 9357.2 |  | 32949.1 | 8506.6 | 29849.3 | 8574.5 |  |
|  | 6 | 36117.5 | 11342.8 | 37493.4 | 6330.9 |  | 30461.2 | 6363.5 | 31205.3 | 8466.7 |  |
|  | 8 | 36704.4 | 11109.1 | 37753.6 | 8502.0 |  | 31918.6 | 6705.9 | 32457.8 | 8309.3 |  |
| CRP | 0 | 1.2 | 1.0 | 1.9 | 1.4 |  | 4.3 | 5.7 | 2.5 | 1.4 |  |
|  | 2 | 1.1 | 0.9 | 1.9 | 1.6 |  | 4.1 | 5.1 | 2.4 | 1.5 |  |
|  | 4 | 1.1 | 0.9 | 1.9 | 1.6 |  | 4.2 | 5.3 | 2.5 | 1.6 |  |
|  | 6 | 1.1 | 0.9 | 2.1 | 1.8 |  | 4.1 | 5.0 | 2.6 | 1.6 |  |
|  | 8 | 1.1 | 1.0 | 2.3 | 2.1 |  | 4.4 | 5.3 | 2.8 | 1.7 |  |
| sgp-130 | 0 | 248.1 | 31.3 | 265.1 | 39.5 |  | 253.2 | 36.0 | 261.1 | 46.0 |  |
|  | 2 | 250.3 | 43.7 | 261.3 | 53.1 |  | 249.5 | 35.8 | 239.3 | 46.1 |  |
|  | 4 | 238.2 | 55.6 | 264.0 | 42.6 |  | 234.1 | 25.2 | 235.8 | 28.1 |  |
|  | 6 | 234.5 | 61.7 | 264.7 | 49.1 |  | 229.8 | 28.5 | 237.5 | 34.0 |  |
|  | 8 | 257.0 | 40.6 | 264.7 | 34.3 |  | 240.4 | 25.2 | 249.9 | 33.6 |  |
