# Supplementary material for: A double blind placebo controlled randomized trial of the effect of acute uric acid changes on inflammatory markers in humans: A pilot study
Source: PLoS One. 2017 Aug 7;12(8):e0181100. doi: 10.1371/journal.pone.0181100 (PMC5546625; doi:10.1371/journal.pone.0181100)
Supplement: S4 Table — (DOCX) [file pone.0181100.s011.docx]

**S4 Table. Mean levels of inflammatory markers (IL-6, IL-6sr, sgp-130, CRP) during uric acid or rasburicase administration.**

| During Drug Administration | | | | | | | | | | |
| --- | --- | --- | --- | --- | --- | --- | --- | --- | --- | --- |
|  |  | Placebo | | Uric Acid | |  | Placebo | | Rasburicase | |
|  | Timept | mean | sd | mean | sd |  | mean | sd | mean | sd |
| IL-6 | 0 | 3.5 | 3.5 | 2.4 | 1.4 |  | 2.7 | 1.5 | 2.3 | 1.1 |
|  | 1 | 3.0 | 2.0 | 3.1 | 2.5 |  | 2.7 | 1.1 | 2.5 | 1.1 |
|  | 2 | 5.0 | 3.5 | 3.9 | 2.9 |  | 4.1 | 2.0 | 3.1 | 1.3 |
|  | 4 | 6.6 | 4.9 | 5.9 | 3.2 |  | 5.6 | 2.7 | 5.3 | 3.2 |
|  | 8 | 7.2 | 4.4 | 9.2 | 3.2 |  | 6.8 | 3.5 | 6.0 | 3.4 |
|  | 12 | 3.7 | 2.8 | 5.9 | 3.7 |  | 6.3 | 3.5 | 4.2 | 2.4 |
| IL-6sr | 0 | 38112.0 | 9207.5 | 40744.5 | 12051.6 |  | 33798.8 | 8746.9 | 32903.0 | 8171.1 |
|  | 1 | 36399.2 | 9205.0 | 42297.8 | 11683.8 |  | 32658.6 | 7216.7 | 31786.5 | 5834.9 |
|  | 2 | 40333.3 | 11183.9 | 46435.0 | 18794.4 |  | 33122.8 | 7481.6 | 32651.2 | 6262.2 |
|  | 4 | 40274.4 | 14624.7 | 47140.7 | 19140.2 |  | 33332.9 | 7486.2 | 34315.2 | 6716.5 |
|  | 8 | 37010.9 | 12854.0 | 45071.4 | 17723.9 |  | 37814.2 | 10244.4 | 35775.7 | 6440.6 |
|  | 12 | 37452.0 | 11625.2 | 42360.4 | 17316.3 |  | 31788.4 | 7005.8 | 32482.1 | 6749.7 |
| CRP | 0 | 1.0 | 0.9 | 1.8 | 0.9 |  | 4.7 | 6.7 | 2.9 | 1.5 |
|  | 1 | 1.0 | 1.0 | 1.7 | 0.9 |  | 4.0 | 6.1 | 2.8 | 1.5 |
|  | 2 | 1.0 | 1.0 | 1.7 | 0.9 |  | 4.4 | 6.5 | 2.8 | 1.5 |
|  | 4 | 1.1 | 1.0 | 1.7 | 0.9 |  | 4.5 | 6.7 | 3.0 | 1.6 |
|  | 8 | 1.2 | 1.0 | 1.7 | 0.9 |  | 4.3 | 6.3 | 2.8 | 1.6 |
|  | 12 | 1.2 | 1.1 | 1.7 | 0.8 |  | 4.3 | 6.4 | 2.8 | 1.6 |
| sgp-130 | 0 | 242.5 | 26.5 | 257.3 | 27.3 |  | 271.1 | 58.4 | 252.2 | 37.4 |
|  | 1 | 243.2 | 26.3 | 250.4 | 42.4 |  | 252.3 | 45.3 | 242.5 | 35.6 |
|  | 2 | 236.0 | 24.2 | 257.2 | 37.4 |  | 247.6 | 44.7 | 244.6 | 33.7 |
|  | 4 | 242.6 | 29.1 | 256.5 | 50.2 |  | 248.7 | 37.4 | 253.8 | 41.4 |
|  | 8 | 239.0 | 24.5 | 264.1 | 45.9 |  | 258.9 | 40.3 | 252.0 | 34.1 |
|  | 12 | 243.6 | 49.9 | 244.1 | 27.1 |  | 227.2 | 34.2 | 242.2 | 45.2 |
