# Supplementary material for: A double blind placebo controlled randomized trial of the effect of acute uric acid changes on inflammatory markers in humans: A pilot study
Source: PLoS One. 2017 Aug 7;12(8):e0181100. doi: 10.1371/journal.pone.0181100 (PMC5546625; doi:10.1371/journal.pone.0181100)
Supplement: S5 Table — (DOCX) [file pone.0181100.s012.docx]

**S5 Table. Mean levels of inflammatory markers (IL-6, IL-6sr, sgp-130, CRP) during oral lipid tolerance test post uric acid or rasburicase administration.**

| Post drug administration lipid tolerance test | | | | | | | | | | |
| --- | --- | --- | --- | --- | --- | --- | --- | --- | --- | --- |
|  |  | Placebo | | Uric Acid | |  | Placebo | | Rasburicase | |
| Cytokine | Time Point | mean | sd | mean | sd |  | mean | sd | mean | sd |
| IL-6 | 0 | 3.8 | 3.2 | 3.6 | 2.2 |  | 6.1 | 3.4 | 2.9 | 1.9 |
|  | 2 | 3.6 | 1.6 | 6.4 | 4.1 |  | 6.7 | 4.0 | 5.2 | 4.1 |
|  | 4 | 4.5 | 2.6 | 6.5 | 3.6 |  | 6.7 | 3.7 | 4.8 | 2.7 |
|  | 6 | 5.2 | 3.1 | 7.9 | 3.8 |  | 7.0 | 4.1 | 5.9 | 3.2 |
|  | 8 | 7.1 | 3.5 | 5.4 | 3.6 |  | 7.1 | 4.5 | 6.6 | 3.4 |
| IL-6sr | 0 | 37117.1 | 9767.4 | 40976.3 | 9763.9 |  | 33314.1 | 8067.3 | 33867.0 | 7044.1 |
|  | 2 | 36069.2 | 9017.4 | 38648.7 | 8412.5 |  | 32134.6 | 7866.8 | 31289.7 | 5739.8 |
|  | 4 | 35480.4 | 7334.6 | 40398.5 | 8668.9 |  | 31065.3 | 7205.8 | 31856.8 | 6815.8 |
|  | 6 | 36497.4 | 8915.2 | 38930.5 | 6998.0 |  | 33312.5 | 7209.0 | 30997.7 | 7059.7 |
|  | 8 | 37185.9 | 8615.2 | 38735.8 | 6950.9 |  | 33384.9 | 7467.4 | 33343.5 | 7791.1 |
| CRP | 0 | 1.4 | 1.2 | 1.6 | 0.9 |  | 4.2 | 6.2 | 2.6 | 1.3 |
|  | 2 | 1.4 | 1.1 | 1.5 | 0.7 |  | 4.2 | 6.4 | 2.3 | 1.2 |
|  | 4 | 1.4 | 1.1 | 1.5 | 0.6 |  | 4.1 | 6.2 | 2.3 | 1.2 |
|  | 6 | 1.5 | 1.2 | 1.5 | 0.6 |  | 2.9 | 5.1 | 2.3 | 1.1 |
|  | 8 | 1.4 | 1.3 | 1.5 | 0.7 |  | 4.3 | 6.6 | 2.2 | 1.1 |
| sgp-130 | 0 | 237.5 | 28.5 | 254.9 | 23.3 |  | 242.0 | 39.8 | 249.5 | 33.5 |
|  | 2 | 255.2 | 28.5 | 260.4 | 34.1 |  | 224.2 | 28.0 | 238.6 | 38.7 |
|  | 4 | 257.5 | 36.9 | 256.9 | 32.5 |  | 221.1 | 29.2 | 234.3 | 36.9 |
|  | 6 | 255.3 | 35.1 | 257.4 | 34.3 |  | 225.4 | 31.7 | 232.4 | 31.4 |
|  | 8 | 274.7 | 33.9 | 267.2 | 36.9 |  | 233.2 | 23.6 | 246.3 | 41.7 |
