# Supplementary material for: A double blind placebo controlled randomized trial of the effect of acute uric acid changes on inflammatory markers in humans: A pilot study
Source: PLoS One. 2017 Aug 7;12(8):e0181100. doi: 10.1371/journal.pone.0181100 (PMC5546625; doi:10.1371/journal.pone.0181100)
Supplement: S1 File — (DOCX) [file pone.0181100.s013.docx]

CONSORT Checklist for randomized clinical trials

| **Items** | **Explanation** | Comments |
| --- | --- | --- |
| ***Title and Abstract*** |  |  |
| 1a. Title | Identification as a randomized trial in the title | Randomized trial is included in the title |
| 1b. Abstract | Structured summary of trial design, methods, results, and conclusions | All components included in abstract |
| ***Introduction*** |  |  |
| 2a. Background | Scientific background and explanation of rationale | Included throughout background |
| 2b. Objectives | Specific objectives or hypotheses | Page 2, line 99 |
| ***Methods*** |  |  |
| 3a. Trial Design | Description of trial design (such as parallel, factorial) including allocation ratio | Lines 107-108  Lines 179 |
| 3b. Changes to trial design | Important changes to methods after trial commencement (such as eligibility criteria), with reasons | Lines 117-120 |
| 4a. Participants | Eligibility criteria for participants | Lines 112-123 |
| 4b. Study settings | Settings and locations where the data were collected | Lines 108-109 |
| 5. Intervention | The interventions for each group with sufficient details to allow replication, including how and when they were actually administered | Lines 126-199 |
| 6a. Outcomes | Completely defined pre-specified primary and secondary outcome measures, including how and when they were assessed | Lines: 169, 191-193, 198-199, 201-208 |
| 6b. Changes to outcomes | Any changes to trial outcomes after the trial commenced, with reasons | Not applicable |
| 7a. Sample Size | How sample size was determined | Lines: 112-114 |
| 7b. Interim analyses and stopping guidelines | When applicable, explanation of any interim analyses and stopping guidelines | Not applicable (the intervention itself was only a day) |
| 8a. Randomisation: sequence generation | Method used to generate the random allocation sequence | Lines: 178-182 |
| 8b. Randomization: type | Type of randomisation; details of any restriction (such as blocking and block size) | Lines: 178-182 |
| 9. Randomisation: allocation concealment mechanism | Mechanism used to implement the random allocation sequence (such as sequentially numbered containers), describing any steps taken to conceal the sequence until interventions were assigned | Lines: 178-182 |
| 10. Randomisation: implementation | Who generated the allocation sequence, who enrolled participants, and who assigned participants to interventions | Lines: 178-182 |
| 11a. Blinding | If done, who was blinded after assignment to interventions (for example, participants, care providers, those assessing outcomes) and how | Lines: 182-184 |
| 11b. Similarity of interventions | If relevant, description of the similarity of interventions | Line: 125-153 |
| 12a. Statistical methods | Statistical methods used to compare groups for primary and secondary outcomes | Line 230-255 |
| 12b. Additional analyses | Methods for additional analyses, such as subgroup analyses and adjusted analyses | Line 230-255 |
| ***Results*** |  |  |
| 13a. Participant Flow | For each group, the numbers of participants who were randomly assigned, received intended treatment, and were analysed for the primary outcome | In figure 1 |
| 13b. Losses and exclusions | For each group, losses and exclusions after randomisation, together with reasons | In figure 1 |
| 14a. Recruitment | Dates defining the periods of recruitment and follow-up | 123-124 |
| 14b. Reason for stopped trial | Why the trial ended or was stopped | Not applicable |
| 15. Baseline Data | A table showing baseline demographic and clinical characteristics for each group | Table 1 |
| 16. Numbers analysed | For each group, number of participants (denominator) included in each analysis and whether the analysis was by original assigned groups | Table 1 |
| 17a. Outcomes and estimation | For each primary and secondary outcome, results for each group, and the estimated effect size and its precision (such as 95% confidence interval) | Primary outcome results in figures |
| 17b. Binary outcomes | For binary outcomes, presentation of both absolute and relative effect sizes is recommended | Not applicable |
| 18. Ancillary analyses | Results of any other analyses performed, including subgroup analyses and adjusted analyses, distinguishing pre-specified from exploratory | Not applicable |
| 19. Harms | All important harms or unintended effects in each group | Line 199 |
| ***Discussion*** |  |  |
| 20. Limitation | Trial limitations, addressing sources of potential bias, imprecision, and, if relevant, multiplicity of analyses | Lines: 404-415 |
| 21. Generalisability | Generalisability (external validity, applicability) of the trial findings | Lines: 416-417 |
| 22. Interpretation | Interpretation consistent with results, balancing benefits and harms, and considering other relevant evidence | Addressed throughout the Discussion |
| ***Other information*** |  |  |
| 23. Registration | Registration number and name of trial registry | Lines 14-15 |
| 24. Protocol | Where the full trial protocol can be accessed, if available | Submitted with the manuscript |
| 25. Funding | Sources of funding and other support (such as supply of drugs), role of funders | Line 145-147, 434-436 |
