# Supplementary material for: A double blind placebo controlled randomized trial of the effect of acute uric acid changes on inflammatory markers in humans: A pilot study
Source: PLoS One. 2017 Aug 7;12(8):e0181100. doi: 10.1371/journal.pone.0181100 (PMC5546625; doi:10.1371/journal.pone.0181100)
Supplement: S2 File — (DOC) [file pone.0181100.s014.doc]

**The Relationship Between Uric Acid and Inflammatory Markers**

**Principal Investigator: Luigi Ferrucci, M.D., P.h.D. ^1^**

**Associate Investigators:**

Dan L. Longo M.D., LI, NIA, NIH

Mark P. Mattson, Ph.D., Lab of Neuroscience, NIA, NIH

Samer Najjar, M.D., Ph.D., Lab of Cardiovascular Science, NIA, NIH

Dan Orenzuk, B.S.P.,RPh, Pharmacist, Clinical Research Branch, NIA, NIH

Dennis D. Taub, Ph.D., Lab of Immunology, NIA, NIH

Richard D. Semba, M.D, Johns Hopkins Medical Institutions, Baltimore, MD

Stephen Waring, M.D.,Ph.D., University of Edinburg, UK

Linda Zukley, Ph.D., R.N, CCRC, Clinical Research Branch, NIA, NIH

Charles Hesdorffer, MBBCh, MMED, Clinical Research Branch, NIA, NIH

**^1^Senior Investigator, NIA**

**BLSA Director, Clinical Research Branch, NIA**

**NIA Clinical Research Unit located at Harbor Hospital**

**National Institute on Aging**

**3001 South Hanover Street**

**Baltimore, MD 21225**

**Phone (410) 350-7330**

**Fax (410) 350-7304**

**Sponsor:** National Institute on Aging / NIH

**CHECK** :

Research Type: X **Clinical Trial**  **Control**   **Natural History**  **Screening**

**TABLE OF CONTENTS**

Precis……………………………………………………………………………….….3

1 Background and Rationale…………………………………………………………….4

2 Objectives………………..…………………………………………………………….8

3 Study Design and Methods………………………………………………………..……..10

4 Patient Eligibility and Exclusion Criteria………………………………………….…11

5 Procedures and Therapeutic Plan……………………………………………………. 13

6 Risks/Discomforts………………………………………………………………...…..22

7 Safety Measures………………………………………………………………………26

8 Grading and Management of Toxicity………………………………………………..28

9 Study Endpoints and Monitoring…………………………………….……………….28

10 Criteria for Withdrawal of Subjects from the Study……………………………….…29

11 Statistical Considerations……………………………………………….…………….30

12 Data Management……………………………………………………………………..31

13 Collection and Storing of Human Sample Specimens………………………..……….32

Intended Use of the Samples/Data…………………………… .…………………....32

Labeling of Stored Samples…………………………………………………………..32

How Samples/Data will be Tracked…………………………………………………..33

Storage and Release of Samples……………………………………………………....33

Stored Samples and Future Studies………………………………………….………..33

What will Happen to the Samples/Data at the completion of the Protocol……………………………………………………………..………………….33

Circumstances that prompt the PI to Report to the IRB Loss or Destruction of Samples/Data……………………………………………………….…………………..34

14 Pharmaceutical Information……………………………………….…………………....35

15 Human Subject Protection…………………………………………………………….. .41

16 Recruitment Plan…………………………………………………………..…………. . 41

17 Benefits……………………………………………………………………………… ..42

18 Compensation……………………………………………………………………… …42

19 Conflict of Interest…………………………………………………………………… .43

20 Confidentiality…………………………………………………………………………..43

21 Consent Process…………………………………………………………………………43

References………………………………………………………………………………………44

Flyers

CV’s

Training certificates (current Human Subject and HIPAA for all investigators)

**Precis**

Uric acid (UA) is a terminal product of purine metabolism with strong antioxidant properties. UA has sizeable concentrations in biologic fluids only in humans and primates because they have a non-functional mutation of the enzyme uricase that, in other animals, transforms UA to allantoin. It has been hypothesized that this mutation has emerged evolutionarily because through this mechanism, humans can counteract the excessive oxidative stress that occurs in many critical pathologic conditions. Partially in contrast with this theory, high levels of UA have been associated with a number of negative health outcomes, including cardiovascular as well as all-cause mortality. Although it is possible that the UA elevation is merely a marker of critical health because it is an “inducible” antioxidant, researchers have questioned whether UA has beneficial or detrimental effect on health status. For example, we recently demonstrated that higher UA levels are cross-sectionally associated with higher levels of pro-inflammatory markers, and predict the increase in inflammatory markers over a 3-year follow-up. Understanding whether UA exerts a protective or detrimental effect on health is important in deciding whether mild hyperuricemia should or should not be aggressively treated.

Unfortunately, observational studies cannot fully address this question. In fact, we cannot exclude that the cross-sectional and longitudinal association between UA, inflammatory markers and negative health outcomes, may simply reflect the fact that UA is an inducible antioxidant that is produced in response to increasing oxidative stress. To verify the hypothesis that UA activates inflammation, we plan to conduct two complementary randomized controlled trials, each one including 10 treated and 10 control subjects. In the first trials, subjects with low UA will be administered 500 mg of UA intravenously. In the second trials, subjects with moderately elevated UA will be administered a single acute dose of Rasburicase. Then inflammatory markers will be measured at multiple points in time, for a total of 32 hours. We hypothesize that an acute increase in the circulating levels of UA will be followed by increasing levels of inflammatory markers. At the same time, an acute reduction of UA levels will be followed by a progressive reduction of inflammatory markers. This study should provide information that will help clinicians to decide whether to or not to treat mild hyperuricemia.

**1. Background and RationalE**

The relationship between UA (UA), inflammation, and chronic diseases is attracting attention in the scientific community. Epidemiological studies suggest that hyperuricemia is an independent risk factor for diabetes, impaired fasting glucose (1), insulin resistance (2), and hypertension in the general population (3-5). High UA concentrations have been associated with both recurrence of stroke (6) and high risk of cardiovascular events in subjects affected by hypertension or pre-existing cardiovascular disease (7). Moreover, UA is a strong negative prognostic factor in patients affected by chronic heart failure (8), and an independent predictor of cardiovascular (9), and all-causes mortality (10).

Since inflammation and oxidative processes are involved in the pathogenesis of all these conditions (11-15) and UA is chemically a strong antioxidant it has been suggested that UA may limit the damage caused by the increased oxidative stress. The antioxidant and, more in general, the biological effect of UA in humans “in vivo” has been little studied. In a series of healthy volunteers, intravenous systemic administration of 1000 mg UA was associated with higher increase of the serum total antioxidant capacity compared to placebo (16,17). In subjects who performed a bout of acute exercise for 20 min, pretreatment with intravenous solution of UA blunted the increase of circulating 8-isoprostaglandin F2α (an oxidation product) that occurs during strenuous exercise compared to controls (18). Recently, a solution with 500 mg of UA has been administered to a group of regular smokers, type 1 diabetics and adult controls who reported an improvement in the endothelial function. The infusion of 500 mg and 1,000 mg UA was well tolerated, with no side effects or change in clinical, hemodynamic and biochemical parameters (16-19).

However, a number of pre-clinical and clinical studies suggest that UA may be a pro-inflammatory factor *per se* (20) and the potential beneficial effects of its antioxidant activities can be partially or completely overcome by its proinflammatory properties.

Preliminary data from our group showed that in a representative sample of community-dwelling elderly participants, abnormally high UA levels as well as UA concentrations in the upper portion of the physiologic range are associated with high levels of several inflammatory markers, such us White Blood Count (WBC), neutrophils, C-reactive protein (CRP), interleukin-6 (IL-6), interleukin-18 (IL-18), interleukin-1 receptor antagonist (IL-1ra), and tumor necrosis factor-alpha (TNF-a) (21). Furthermore, baseline UA concentrations and UA changes significantly predicted IL-6 and CRP levels after three years (22). These findings are consistent with the association between serum UA and CRP levels which has been described in previous epidemiological studies (23)

Circulating UA levels increase over the lifespan as observed in a large observational longitudinal population-based study (24). Since aging has been consistently associated with higher levels of circulating inflammatory markers (25), a possible pro-inflammatory activity of UA may contribute to the mild inflammatory state of the elderly. Exploring the possible causes of chronic inflammation in the older population is important because inflammation is one of the fundamental pathophysiological mechanisms underlying the major chronic diseases and the development of disability in the elderly.

Although the intra-articular pro-inflammatory activity of monosodium urate (MSU) crystals, the saline form of UA involved in the pathogenesis of gout, has been extensively investigated, it is still unclear whether soluble UA can trigger a systemic inflammatory response. In gouty arthritis, MSU crystal deposition initiates, amplifies and sustains the local inflammatory processes. There is strong evidence that UA crystals stimulate the release of chemokines and pro-inflammatory cytokines including TNF-a, interleukin-1beta (IL-1b) and IL-6. At present, the evidence in favor of the pro-inflammatory role of soluble UA is limited to few studies performed in vitro and in experimental animals (26-28) and no data is available from human models.

The hypothesis of a pro-inflammatory role of UA contradicts current views on the physiology of UA in humans. As mentioned above, UA is thought to be a powerful antioxidant which is produced physiologically to limit the radical oxygen species (ROS)-mediated cell damage during severe oxidative stress conditions. It has been proven that ROS can trigger an inflammatory response via activation of the Nuclear Factor k-B (NF-kB) pathway. Thus, UA by blocking the effect of ROS should be theoretically considered also an anti-inflammatory factor, and the most important and powerful circulating antioxidant compound.

Understanding the relationship between UA and inflammation is essential to plan effective treatment strategies in clinical practice: if UA levels in the upper portion of the normal range do not contribute to the onset/worsening of inflammation but rather limit the ROS damage in particular pro-inflammatory states, then treatment should be limited to patients who have UA concentrations associated with risk of crystal precipitation. On the contrary, if soluble UA directly stimulates the inflammatory response and this potentially harmful mechanism overcomes the potential benefits related to antioxidant activity, then the treatment should be started early, when UA concentration are still in the upper portion of the normal range.

**UA, Inflammation, and Oxidative Stress: Possible Molecular Pathways**

Multiple and potentially self-maintaining biologic pathways linking UA with inflammation are theoretically possible (Figure 1). Only some pieces of this hypothetical molecular puzzle have been experimentally confirmed and mostly in animal models.

**Figure 1**: The theoretical pathways of the relationship between UA, inflammation and oxidative stress. ROS: Radical Oxygen Species; NFkB: Nuclear Factor-kappa B; XDH: Xanthine Dehydrogenase; XO: Xanthine Oxidase.

Conceptually, three non-mutually exclusive mechanisms can be hypothesized:

A) UA causes inflammation by direct or indirect stimulation of NF-kB (Figure 2).

B) Pro-inflammatory cytokines stimulate the UA production by up-regulating xantine-oxidase (XO), the enzyme that catalyzes the transformation of hypoxantine to UA, leading to increase in UA (Figure 3).

C) A “third factor” stimulates in parallel the synthesis of both pro-inflammatory cytokines and UA. The third factor may increase cell death (apoptosis), or increases ROS production (Figure 4).

**Hypothesis A: UA causes inflammation by directly or indirectly stimulating NF-kB.**

This hypothesis is supported by the results of experiments conducted on mouse smooth muscle cells. The addition of UA in the culture medium activates the intracellular cascade leading to the activation of the NF-kB and AP-1 pathway (via ERK1/2 and p38/MAPK). NF-kB is an essential intracellular factor which regulates, at nuclear level, the transcription of virtually all pro-inflammatory cytokines. The intracellular events triggered by UA determine the production of monocyte chemoattractant protein-1 (MCP-1) and platelet-derived growth factor (PDGF) (29, 30) which are molecules involved in the inflammatory response and in the atherogenetic processes.

Additionally, it has been shown that UA stimulated the production of pro-inflammatory cytokines in mouse mononuclear cell lines. The authors suggested that this effect is mediated by the activation of the NF-kB pathway. However, to the best of our knowledge, this study has only been presented in a scientific meeting and never published in a peer-reviewed journal.

**Hypothesis B: Pro-inflammatory cytokines stimulate the UA production.**

Pro-inflammatory cytokines up-regulate xantine oxidase (XO) activity, which catalyzes the transformation of hypoxantine to UA, leading to increasing UA production. This hypothesis is supported by previous studies showing that the XO gene expression is markedly stimulated by the pro-inflammatory cytokines TNF-α, IL-1β and IFN-γ in bovine renal epithelial cells (31), rat alveolar macrophages (32), and human mammary epithelial cells (33). In addition, a recent study

demonstrated enhanced XO activity in the lungs of rats insufflated with IL-1 and IFN-γ (34). Evidence that TNF-α and IL-1β upregulate the XO gene transcription have been also found by studying airway lining fluid in patients with COPD(35).

**Hypothesis C: A “third factor” stimulates in parallel the UA and cytokines synthesis**. The most likely factor that stimulates the production of both pro-inflammatory cytokines and UA may be an excess of oxidative stress caused by increased ROS production, which can activate the NF-kB pathway (36). The NF-kB activation may increase inflammation, and in turn upregulate xantino-oxidase (see hypothesis B). Moreover, ROS may cause direct and indirect DNA damage (37) increasing the release of damaged purines during the DNA repair. Since purines are the physiologic substrate for XO activity, ROS may up-regulate the XO activity and increase the UA levels, indirectly. Higher XO enzyme activity may in parallel cause higher ROS levels (through a mechanism that involves Vit A) and through this mechanism stimulate the NF-kB pathway bringing about cytokine production in a vicious cycle.

**2. Objectives**

Primary Objective

The primary outcome is to ascertain whether UA is a pro-inflammatory compound. This aim will be tested both by measuring the concentration of inflammatory markers after short term changes in UA, and by testing the hypothesis that different concentrations of UA modify the inflammatory response induced by a lipid tolerance test.

Secondary Objectives

To document the effect of increasing versus decreasing UA levels on circulating antioxidant biomarkers. To test the hypothesis that increasing levels of UA are associated with higher mitochondrial efficiency as indicated by higher MRI-spectroscopy estimated rate of phosphocreatinine resynthesis after exercise.

Both primary and secondary aims will be tested using two different trials whose results will be complementary. In the first trial (Trial A: UA versus Placebo), the primary and secondary outcomes will be estimated after raising the circulating UA levels by systemic UA infusion.

In the second trial (Trial B: Rasburicase versus Placebo), the primary and secondary outcomes will be estimated after lowering the circulating UA levels by systemic infusion of Rasburicase, a recombinant form of urate oxidase that is already approved for clinical use.

In both Trials, measures will be conducted before and after the intervention and the analysis will be focused on a comparison between active treatment and placebo groups.

**Rationale for the double approach**

Using treatments that result in both an increase and a decrease in the circulating UA levels will help to clarify whether UA is a causal, compensatory or co-incidental factor associated with modification of the inflammatory status.

**In Trial A:** UA versus Placebo, UA systemic infusion will rapidly increase the circulating UA levels. We expect that the sudden increase of circulating UA levels may directly affect circulating levels of cytokines and antioxidant biomarkers and affect (increase or reduce) the inflammatory response induced by a lipid tolerance test.

**In Trial B:** Rasburicase versus Placebo, Rasburicase systemic administration will rapidly lower the circulating UA levels. We expect that a sudden decrease of circulating UA levels may affect circulating cytokines and antioxidant biomarkers as well modify the inflammatory response induced by a lipid tolerance test with an opposite fashion compared to Trial A. Both short term approaches of Trial A: UA versus Placebo and Trial B: Rasburicase versus Placebo are likely to allow a better understanding of the possible mechanisms that link changes in circulating UA levels and increased cardiovascular risk. A similar approach has been used by previous studies exploring the effect of sudden changes of saturated fats or circulating homocysteine concentrations, on physiological parameters (39, 40).

**3. Study Design and Methods**

This is a prospective placebo-controlled study, which consists of two inter-related trials, Trial A: UA versus Placebo and Trial B: Rasburicase versus Placebo respectively. Participants will be community-dwelling women and men, aged 50-75 years with BMI 23-34.9 kg/m^2^. All participants must be capable of attending an outpatient setting at the required protocol time-points.

Participants will be randomized into either Trial A or Trial B: Trial A: UA versus Placebo; and Trial B: Rasburicase versus Placebo. All specific inclusion/exclusion criteria are explained in detail in the remainder of this document.

According to the sequence of enrollment, eligible participants will be randomly assigned to treatment or placebo until 5 pairs of participants (1 intervention and 1 control subject) have been enrolled in each cohort (see the follow Enrollment schema). The randomization list will be maintained by the protocol study monitor. Two sealed copies of the randomization list will be stored at the NIA; one with the PI and one secured in the NIA Research Pharmacy. Study investigators and participants will be blinded to the treatment/placebo assignments. However, NIA research pharmacist or a person designated by him/her will not be blinded, since the medications will be prepared and dispensed to subjects from the NIA Research Pharmacy. The P.I. or designated NIA physician will review the laboratory results with the identity of the subject kept anonymous.

**Trial A: UA versus Placebo**

Twenty participants will be enrolled in this trial, 10 men and 10 women respectively. In each sex group, 5 individuals will be randomly assigned to intervention and 5 to placebo treatment. All participants should have UA ≤10.0 mg/dL and satisfy the inclusion/exclusion criteria reported in section 4C.

**Trial B: Rasburicase versus Placebo**

Twenty participants will be enrolled in this cohort, 10 men and 10 women. In each sex group, 5 individuals will be randomly assigned to intervention and 5 to placebo treatment. All participants should have UA ≤10.0 mg/dL and satisfy the inclusion/exclusion criteria reported in section 4C.

Enrollment schema

**Screening:**

**Uric Acid ≤10.0 mg/dL**

**Trial A**

**Uric Acid**

**≤ 10.0 mg/dL**

**Trial B**

**Men (10)**

**Random Allocation**

**Women (10)**

**Random Allocation**

**Men (10)**

**Random Allocation**

**Women(10)**

**Random Allocation**

**Screening:**

**Age 50**

**-**

**75 yrs**

**BMI 23**

**-**

**34.9 Kg/m2**

**Eligibility: Inclusion/Exclusion criteria are satisfied**

**5 Cases**

**5 Controls**

**5 Cases**

**5 Controls**

**5 Cases**

**5 Controls**

**5 Cases**

**5 Controls**

**Enrollment**

**4. Patient Eligibility and Exclusion Criteria**

*a. General Inclusion criteria valid for both trials*

1. Age 50-75 years
2. BMI 23-34.9 kg/m^2^
3. Estimated GFR >60 mL/min
4. Blessed mental score equal to or less than 3
5. Ability to fully participate in an informed consent process

6. For Trial A (UA versus Placebo): all participants should have UA ≤10.0 mg/dL

7. For Trial B (Rasburicase versus Placebo): all participants should have UA ≤10.0 mg/dL

*b. General Exclusion criteria for potential participants of both trials*

1. Uric Acid level greater than 10.0 mg/dL.
2. History of hypersensibility or intolerance to Rasburicase and/or to Lithium
3. Chronic Kidney Disease.
4. History of kidney stones.
5. History of asthma, atopic allergies, hemolytic or methemoglobinemia reactions
6. History of unstable angina, cardiac arrhythmia, stroke or myocardial infarction within 3 months, open-heart surgery within 6 months of the study enrollment
7. History of drug or alcohol dependence, or positive urine toxicology
8. Past or present positive test for HBV, HCV or HIV (base on blood test).
9. Glucose-6-Phosphate Dehydrogenase deficiency (G6PD-deficiency)*
10. History of gout
11. Last menstruation occurred less than 1 year ago
12. Unintentional or intentional weight loss of 5 kg in the previous 6 months
13. Alcohol daily intake >30 grams (more than 2 beers daily, more than 2 glass of wine or cocktail daily)
14. Current smokers or former smokers (stopped smoking less than 1 year before)
15. Blood pressure > 150/90 mmHg
16. Diabetes mellitus on dietary or pharmacological treatment
17. Hb <11.5 g/dL in women or < 12.5 g/dL in men
18. Acute infection/inflammation (CRP>5 mcg/mL) for Trial A: UA versus Placebo, CRP >10mcg/L for Trial B: Rasburicase versus Placebo, or any clinical overt chronic infections including periodontal disease.
19. Active liver disease or liver function tests greater than 2 times upper normal limit
20. Self reported inflammatory joints or autoimmune diseases
21. Treatment over the last 3 months with Allopurinol, Rasburicase
22. Treatment over the last 3 months with Warfarin, Dicumarol, Theophylline, Statins
23. Necessity of daily treatment with NSAID
24. Current hormone replacement
25. Ongoing glucocorticoids, corticosteroids, including prednisone and/or cortisone injections or inhalations. (topical steroid cream use and occasional corticosteroid administration over the last 3 months is acceptable)
26. Current daily antioxidants or/and multivitamin supplementation or over the last month
27. Life-threatening diseases including malignancies
28. Recent Blood Donation (last 56 days)
29. Any other reason that based on the PI’s judgment requires exclusion from this study
30. Inability to obtain venous access.

*The Glucose-6-Phosphate Dehydrogenase activity will be assessed in each eligible participant randomly assigned to Trial B. The test for G6PD deficiency will require 3.5 ml of blood.

^#^Participants will be asked to refrain from taking any NSAIDs during the five days prior before the baseline evaluation and over the course of the study with the exception of Aspirin 100 mg/daily or less.

**5. Procedures and Therapeutic Plan**

In each of the two Trials, 5 women and 5 men will be assigned to receive treatment and 5 women and 5 men will be assigned to receive placebo. There will be no recognizable labels treatment/placebo on the drug bag, and the NIA research pharmacist will be the only person unblinded to the random assignment to treatment or placebo.

**Trial A: UA versus placebo**

Treatment: UA 500 mg in 250 ml of 0.1% lithium carbonate/ 4% dextrose solution infused over a 60 minute period on visit 3.

Placebo: 250 ml of 0.1% lithium carbonate/ 4% dextrose solution infused over a 60 minute period on visit 3.

**Trial B: Rasburicase versus Placebo**

Treatment: Rasburicase 0.15 mg/kg in 250 ml 0.9% sodium chloride solution infused over a 60 minute period on visit 3.

Placebo: 250 ml of 0.9% sodium chloride solution infused over a 60 minute period on visit 3.

Both treatment and placebo administration will be done only once and according to the same procedures. Treatment and placebo administration will be done in double-blind fashion and under continuous physician’s supervision. Only the NIA research Pharmacist or someone designated by him will be aware of the participant random assignment to treatment or placebo.

**Schedule of visits**

For each Trial, participants will be scheduled for a total of 4 visits including one overnight stay lasting 2 nights and will undergo the same clinical and laboratory procedures. The table below summarizes the tests and procedures to be done during each of the visit. After 2 weeks, participants will be considered off study.

**Clinical and laboratory procedures in both trials**

The clinical and laboratory procedures will be articulated into two main steps. The first part will be a screening of the potential participants, while the second part will be a comprehensive assessment of participants’ clinical characteristics and collection of blood samples. Blood drawn during the screening visit will be sent to Harbor Hospital. On subsequent visits, sample for assay of the inflammatory markers will be sent to the core lab, divided into aliquots banked at the NIA’s Core Lab, and at the end of the study sent to the different laboratories to perform the appropriate assays.

|  | **Visit 1**  **(T0)** | **Visit 2**  **(T1)** | | | | | | **Visit 3**  **Inpatient**  **(T2)** | | | | | | | | | | | | | **Visit 4**  **(T3)** |
| --- | --- | --- | --- | --- | --- | --- | --- | --- | --- | --- | --- | --- | --- | --- | --- | --- | --- | --- | --- | --- | --- |
|  | Outpatient | Outpatient  (up to 1 week after Visit 1 and 2 days before Visit 3) | | | | | | Day 1 | | | | | | | Day 2  (24 h from intervention is the baseline for the second Oral Lipid Tolerance Test) | | | | | | Outpatient |
| **Type of Visit** | Screening | Baseline | 0h | 2h | 4h | 6h | 8h | Baseline | Intervention | 1 h | 2 h | 4 h | 8 h | 12 h | 24 h | 0 h | 2 h | 4 h | 6 h | 8 h | 2 weeks |
| Medical History/  Physical Exam | X | X |  |  |  |  |  |  |  |  |  |  |  |  |  |  |  |  |  |  | X |
| EKG | X |  |  |  |  |  |  |  |  |  |  |  |  |  | X |  |  |  |  |  | X |
| HIV, HBV, HCV | X |  |  |  |  |  |  |  |  |  |  |  |  |  |  |  |  |  |  |  |  |
|  |  |  |  |  |  |  |  |  |  |  |  |  |  |  |  |  |  |  |  |  |  |
| Blood tests1 | X |  |  |  |  |  |  |  |  |  |  |  |  |  | X |  |  |  |  |  | X |
| Urine analysis | X |  |  |  |  |  |  |  |  |  |  |  |  |  |  |  |  |  |  |  | X |
| Urine Toxicology2 | X |  |  |  |  |  |  |  |  |  |  |  |  |  |  |  |  |  |  |  |  |
| Urine Pregnancy | X | X |  |  |  |  |  | X |  |  |  |  |  |  |  |  |  |  |  |  |  |
| Urine Specific Gravity |  |  |  |  |  |  |  | X |  |  | X |  |  |  | X |  |  |  |  |  |  |
| Urine PH |  |  |  |  |  |  |  | X |  |  | X |  |  |  | X |  |  |  |  |  |  |
| Urine for Uric Acid |  |  |  |  |  |  |  | X |  | X | | | | | X |  |  |  |  |  | X |
| Eligibility, Consent | X |  |  |  |  |  |  |  |  |  |  |  |  |  |  |  |  |  |  |  |  |
| Randomization |  |  |  |  |  |  |  | X |  |  |  |  |  |  |  |  |  |  |  |  |  |
| Lipid Tolerance Test3 |  | X |  | X | X | X | X |  |  |  |  |  |  |  | X |  | X | X | X | X |  |
| Basal Metabolic Rate |  |  |  |  |  |  |  | X |  |  |  |  |  |  | X |  |  |  |  |  |  |
| Vital signs | X | X |  |  |  |  | X | X |  | X | X | X | X | X | X |  |  |  |  | X | X |
| Intake and Output |  |  |  |  |  |  |  | X | X | X | X | X | X | X |  |  |  |  |  |  |  |
| UA + Hypoxanthine |  |  |  |  |  |  |  | X |  | X | X | X | X | X | X |  |  |  |  |  | X |
| PAXgene (RNA) |  |  |  |  |  |  |  | X |  |  |  |  |  | X | X |  |  |  |  |  | X |
| DNA Extraction |  | X |  |  |  |  |  | X |  |  |  |  |  |  |  |  |  |  |  |  |  |
| Inflammatory markers4 |  | X |  | X | X | X | X | X |  | X | X | X | X | X | X |  | X | X | X | X | X |
| Antioxidant markers5 |  |  |  |  |  |  |  | X |  | X | X | X | X | X | X |  |  |  |  |  | X |
| Ox damage markers6 |  |  |  |  |  |  |  | X |  | X | X | X | X | X | X |  |  |  |  |  | X |
| MRI Spectroscopy |  | X |  |  |  |  |  |  |  |  |  |  |  |  | X |  |  |  |  |  |  |
| Blood volume draw | **26.5 ml** | **45 ml** | | | | | | **256.5 ml** | | | | | | | | | | | | | 49 ml |

l

**Visit 1: Screening Tests**

Prior to attending the screening visit, each potential participant will be asked to fast overnight (> 8 hours). This screening visit will be conducted under the NIA Uric Acid protocol number 2008-322. After the reviewing the study schema in detail and obtaining protocol, HIPAA, and HIV consents, potential participants will be screened for study eligibility by assessing medical history, physical examination, blood draw (26.5 ml), routine urinalysis, vital signs, height and weight (used to compute BMI) and waist circumference. EKG will be performed at baseline. Cognitive status will be assessed using the Blessed Mental Test and potential participant with a score> 3 will be excluded. Complete CBC with differential count, glucose, BUN, creatinine, UA, LDH, CPK, electrolytes (Na+, K+, Cl-; Ca++, Mg+), AST, ALT, ALP, albumin, globulin, bilirubin (total and direct), haptoglobin, HDL-LDL and Total cholesterol, triglycerides, and CRP will be estimated from blood samples. We will also test for HCV, HBV and HIV. The Glucose-6-Phosphate Dehydrogenase activity will be assessed in each eligible participant randomly assigned to Trial B (individuals who are glucose-6-phosphate dehydrogenase deficient cannot break down hydrogen peroxide, a byproduct of rasburicase, leading to hemolysis). In addition, we will tests potential participants for drug use (urine toxicology test). Urine analysis will be also performed to rule out urinary infections. Finally, urine pregnancy tests will be performed on all women of childbearing age. Since the average age of menopause in the U.S. in 51±4.1 years, all women under age 60 will be considered of childbearing age, with the exception of those who have had their uterus surgically removed. All these tests are aimed at screening for conditions that are considered exclusion criteria for this study, and are performed according to standard clinical methods. A physician will review subject’s medical history, physical exams, EKG and biological tests in order to evaluate the participant’s eligibility criteria. Subjects who are found to be eligible for the study will be scheduled for Visit 2. Those assessed as ineligible will be notified within one week. When notified of eligibility, participants will be instructed on NPO criteria for Visit 2. The participants will receive the following instructions written on an hand-out: 1. To maintain the same dietary habits they had before being involved in the study; 2. In particular not to change their dietary habits by increasing high-purine sea foods, such as anchovies, sardines, herring, mackerel, and scallops, high-purine meats, such as game meats, liver, beef, kidney, brains, meat extracts (e.g. Oxo, Bovril), meat gravys, pork, poultry, and sweetbreads, and high-purine vegetables, such as asparagus, green peas, cauliflower, spinach and mushrooms, lentil, dried peas and beans, and to continue to consume vegetables, oatmeal, wheat bran and wheat germ in the same portions as before. Participants will be advised that changes in their habitual diet may result in a substantial change of their circulating UA levels and that, if so, their participation in the study will be discontinued.

**Visit 2: history, physical exam and preliminary test**

Participants will arrive at NIA Clinical Research Unit after overnight fasting for 8 hours. A brief physical examination will be performed, including a sample of blood that will be collected for DNA extraction and characterization of the genes that indirectly or directly affect the UA metabolism. A physician will review subject’s eligibility criteria and immediately after the participant will be administered an MRI-spectroscopy that estimates the rate of phosphocreatinine resynthesis after exercise). Next, an Oral Lipid Tolerance Test (OLTT) will be performed. The OLTT consists of eating a standard fast-food type meal (with approximately 900 cal and 50 grams of fat), and measuring changes in lipids and inflammatory markers at 2 hour intervals for total of 8 hours. The meal will be prepared by a registered dietitian and will be consumed in no longer than 20 minutes. The inflammatory markers that will be sampled consist of IL-6, IL-6sr, TNF-α, TNF-r1, TNF-r2, sgp 130, and hsCRP (+Tryglycerides). However, based on the information available at the end of the study, some of these inflammatory markers may not be measured and may be substituted with markers without changing the quantity of blood collected. OLTT will start in the morning after 8 hours of fasting. After the meal, subjects will not eat for the next 8 hours but will have free access to water and ice. After 8 hours a standard meal will be available. Blood samples will be drawn before the meal and every 2 hours after the first bite of the meal over an 8-hour period. The total amount of blood needed for the tests/assays included in the OLTT will be 40 mL including a 1mL waste at each time point. The blood will be sent via courier to NIA Lab at the 2 hour intervals. From there the blood will be sent to the respective labs for processing. After discharge, the patient will be given instruction for the following visit. In particular, participants will be advised to drink 8 8oz glasses of water on the day before the visit and at least 2 further glasses in the morning before the infusion.

**Visit 3: Baseline and short-term post-intervention**

Visit 3 will be scheduled at least 2 days after Visit 2. Participants will be asked to arrive the evening before Visit 3 for a two consecutive overnight stay at the NIA Clinical Research Unit. Below is the sequence of exams and blood tests scheduled for Visit 3. As mentioned above, participants will be requested to drink at least two glasses of water and eat a light breakfast (cold cereal, milk, coffee or tea and bread and butter with butter and/or jelly in the morning). If for some reason, the study participant does not consume an appropriate amount of water on their own 500 mL of normal saline 0.09% will be administered intravenously prior to the study intervention. Based on their characteristics (UA serum level) and sequence of enrollment, participant will be assigned a randomization envelope. After opening the envelope and compiling the included randomization form, the NIA research pharmacist assigns the participant to the treatment or placebo group and prepares the study drug. Information on randomization will be maintained by the NIA research pharmacy in a safe but known place, accessible in case of emergencies. At 4:00 pm following the treatment, participants will eat a dinner consisting of a sandwich and a bag of chips. They may have a soda, coffee, tea or milk. The participant will be not be able to eat again until morning for the lipid tolerance test but will be given water freely.

*1. Basal Metabolic Rate (BMR)*

Basal Metabolic Rate will be estimated in Visit 3 at baseline and on the next day after the intervention. The measurement will be performed in the morning after overnight fasting at the NIA Clinical Research Unit. RMR will be measured before any other evaluation using the COSMED K4 b2 portable indirect calorimeter.

*2. Vital sign measurements*

Blood pressure, pulse rate, respirations and core body temperature will be measured at baseline and at each evaluation time during the following 24 hours. Pulse rate and blood pressure will be estimated using an automated cuff. Core temperature will be estimated using a sublingual thermometer. An EKG will be obtained 24 hours after the beginning of the infusion.

*3. Infusion of the Study Drug*

In Trial A (Uric Acid vs Placebo), infusion of the drug will be started only if the specific gravity of the urine is less than 1.015. In both trials A and B, treatment or placebo will be infused using an IV infusion pump over a 60 minute time period. When the infusion is completed the line will be discontinued.

After the infusion of the study drug, participants will be encouraged to drink at least 8 8oz glasses of water over the next 12 hours. However, if they are unable to drink enough water, 1000 mL of saline solution will be administered intravenously. To obtain mildly alkaline urine, all subjects enrolled in Trial A will receive 7 oral doses of 650 mg sodium bicarbonate (total 4,550 mg = 54.6 mEq) according to the following timeline: 1 tablet in the evening before Day 1 of Visit 2; one tablet in the morning of visit 2; one tablet at the beginning of study drug administration: one tablet 4, 8, 12 and 24 hours after the study drug administration.

*4 Blood sampling*

Baseline fasting blood sampling will be done using an IV access different from the one used for infusing the study drug. An intravenous cannula will be inserted into a large vein using a standard aseptic technique and, if requested from participants, a local anesthesia. The IV site will be kept open by a KVO rate for continuous saline solution infusion, and will be used to draw blood samples. The cannula will be removed at the end of this visit. The total amount of blood drawn for this visit is 256.5 mL including 1 mL waste at each timepoint, and the breakdown is listed below. During the infusion the samples for the following tests will be collected: uric acid and hypoxanthine (monitoring), inflammatory markers, circulating antioxidants, markers of oxidative damage and RNA expression. Some of the blood collected will be used to create aliquots of serum and plasma that will be stored to measure other biomarkers relevant for this study based on the most current literature.

*5. Inflammatory markers*

Blood samples for measuring proinflammatory markers will be collected at baseline and after 1, 2, 4, 8, 12, and 24 hours after the beginning of the infusion. At each of the successive blood sample draws, IL-1ra, IL-6, IL-6r, IL-18, IL-1β, TNF-α, TNF-r1, TNF-r2, sgp130 and hsCRP will be assessed in duplicate using high sensitivity ELISA assays. Based on the information available at the end of the study, some of these inflammatory markers may not be measured and may be substituted with markers without changing the quantity of blood collected. The total amount of blood per each time we perform these assays will be 3.5 mL.

*6. Circulating Antioxidant markers*

Participants will be tested for Vitamin C, Carotenoids and Selenium at baseline and each of the successive blood sample draws. Serum levels of vitamin C will be assayed using HPLC with 1-methylUA used as an electrochemically active internal standard. Serum levels of carotenoids will be determined by high performance liquid chromatography (HPLC). Selenium will be measured by graphite furnace atomic absorption spectrometry using a Perkin Elmer Analyst 600 with Zeeman background correction. The total amount of blood per each time we perform these assays will be equal to 4.5 mL.

*7. Markers of Oxidative Damage*

Participants’ blood samples will be tested for markers of oxidative stress damage, including but not limited to F2-Isoprostane (marker of lipid peroxidation) and oxidized/reduced glutathione ratio at baseline and after 1, 2, 4, 8, 12, and 24 hours from the beginning of the infusion. Based on the information available at the end of the study, other emerging more reliable markers of oxidative stress may be also measured without changes in the quantity of blood collected.

*8. RNA microarray expression*

Whole Blood samples for microarray RNA expression will be collected on PAXgene (QIAGEN/BD) tubes at baseline, and at 12 and 24 hours after the beginning of the infusion.

*9. Uric Acid*

A uric acid and hypoxanthine level will be measured level will be measured at baseline, after 1, 2, 4, 8, 12 and 24 hours and after 2 weeks after the beginning of the infusion. Approximately 3.5mL of blood will be drawn for this at each time-point.

*10. Follow up Blood work*

Total and differential white and red blood counts, hemoglobin, hematocrit, platelets, glucose, BUN, creatinine, Uric Acid, LDH, CPK, electrolytes, AST, ALT, ALP, albumin, globulin, bilirubin (total and direct), haptoglobin, HDL and total cholesterol, triglycerides, and CRP will be drawn at the 24 hour timepoint to make sure that there has been no change post intervention. The total amount of blood needed for this will be 15 mL.

*11. Urine collection*

A urine sample will be collected at baseline (first morning void on Day 1), every void between 1 and 12 hours after infusion and 24 hours after infusion. The urine samples will be used to evaluate uric acid levels in the urine and potentially markers of oxidative stress damage.

*12. MRI muscle spectroscopy.*

Magnetic resonance spectroscopy (MRS) will be used to study energy metabolism in muscle during and after exercise. As already mentioned, MRS is performed once on Visit 2 and a second time 24 hours after the infusion of the study drug, immediately before performing the second Lipid Tolerance Test. Participants will be carefully screened for MRI contraindications before performing the tests. Also, they will be given a choice of receiving the MRI. Participants with contraindication to MRI or refusing MRI will not receive MRI but will be still enrolled in the other parts of the study, without any effects on their compensation.

*13. Lipid Tolerance Test*

24 hours after the injection, A second OLTT will be performed using exactly the same protocol used in Visit 2.

**Visit 4: 2 weeks post-intervention**

A follow-up visit will be scheduled two weeks after treatment. This visit is mostly for checking that all physiological parameters return to the baseline levels and there are no side effects. Blood samples will be collected at the NIA Clinical Research Unit in the morning after 8 hours overnight fasting and used to measure: complete CBC with differential count, glucose, BUN, creatinine, UA, LDH, CPK, electrolytes (Na+, K+, Cl-; Ca++, Mg+), AST, ALT, ALP, albumin, globulin, bilirubin (total and direct), haptoglobin, HDL-LDL and Total cholesterol, triglycerides, and CRP will be estimated from blood samples. An EKG will be obtained. In addition, samples will be obtained to measure inflammatory markers, circulating antioxidants, markers of oxidative damage and RNA expression (see point 5-8 above for full details).

The total amount of blood drawn on this visit is 49 mL. An additional visit may be scheduled at 4 weeks from intervention for participants whose UA levels are not back to the baseline levels.

**Total volume of blood draws**

A total of 372 mL of blood including discard will be collected from each participant over the course of the study. This amount of blood does not exceed the amount of blood taken by the American Red Cross or a single donation of 450 ml or one pint over any given 8-week period. A healthy person can safely donate this amount of blood every 8 weeks. All the tests performed in this protocol are summarized in the following table:

**Schema of Blood Collection/Analysis**

|  | **Visit 1**  **(T0)** | **Visit 2**  **(T1)** | | | | | | **Visit 3**  **Inpatient (T2)** | | | | | | | | | | | | | **Visit 4**  **(T3)** |
| --- | --- | --- | --- | --- | --- | --- | --- | --- | --- | --- | --- | --- | --- | --- | --- | --- | --- | --- | --- | --- | --- |
|  | Outpatient | Outpatient | | | | | | Day 1 | | | | | | | Day 2 | | | | | | Out |
| **Type of Visit** | Screening | Base | 0 | 2 | 4 | 6 | 8 | Base |  | 1 | 2 | 4 | 8 | 12 | 24 | 0 | 2 | 4 | 6 | 8 | 2 weeks |
| Blood tests^1^ | **X** |  |  |  |  |  |  |  |  |  |  |  |  |  | **X** |  |  |  |  |  | **X** |
| Lipid Tolerance Test^2^ |  | **X** |  | **X** | **X** | **X** | **X** |  |  |  |  |  |  |  | **X** |  | **X** | **X** | **X** | **X** |  |
| G6PD activity^3^ | **X** |  |  |  |  |  |  |  |  |  |  |  |  |  |  |  |  |  |  |  |  |
| UA + Hypoxanthine^4^ |  |  |  |  |  |  |  | **X** |  | **X** | **X** | **X** | **X** | **X** | **X** |  |  |  |  |  | **X** |
| PAXgene (RNA)^5^ |  |  |  |  |  |  |  | **X** |  |  |  |  |  | **X** | **X** |  |  |  |  |  | **X** |
| DNA Extraction^6^ |  | **X** |  |  |  |  |  | **X** |  |  |  |  |  |  |  |  |  |  |  |  |  |
| Inflammatory markers^7^ |  | **X** |  | **X** | **X** | **X** | **X** | **X** |  | **X** | **X** | **X** | **X** | **X** | **X** |  | **X** | **X** | **X** | **X** | **X** |
| Antioxidant markers^8^ |  |  |  |  |  |  |  | **X** |  | **X** | **X** | **X** | **X** | **X** | **X** |  |  |  |  |  | **X** |
| Ox damage markers^9^ |  |  |  |  |  |  |  | **X** |  | **X** | **X** | **X** | **X** | **X** | **X** |  |  |  |  |  | **X** |
| Blood volume draw | 26.5 ml | 45 ml | | | | | | 256.5 ml | | | | | | | | | | | | | 49 ml |

**1.** Complete CBC with differential count, Glucose, BUN, Creatinine, UA, LDK, CPK, Electrolytes (NA+, K+, CL-; CA++, MG+), AST, ALT, ALP, Albumin, Globulins, Bilirubin (total and direct), Haptoglobin, LDL-HDL and total cholesterol, Triglycerides, and CRP.

**2.** Measures obtained at each point: IL-6, IL-6sr, TNF-α, TNF-r1, TNF-r2, sgp 130, and hsCRP +Tryglycerides+1 mL of serum for banking purposes.

**3.** G6PD activity, once in the study.

**4.** UA+Hypoxanthine..

**5.** PAXgene, 5 mL per point. Total volume for 4 points 20 mL.

**6.** DNA extraction, once in the study: Total volume 5 mL.

**7.** Measures obtained at each point: IL-1ra, IL-6, IL-6r, IL-18, IL-1β, TNF-α, TNF-r1, TNF-r2, sgp130 and hsCRP + 1ml of serum for storage.

**8.** Measures obtained at each point: Vitamin C, Carotenoids and Selenium + 1ml of serum for storage.

**9.** Measures obtained at each point: F2-Isoprostane + oxidized/reduced glutathione ratio + 1ml of serum for storage.

Schema of Urine Collection/Analysis

|  | **Visit 1**  **(T0)** | **Visit 2**  **(T1)** | | | | | | **Visit 3**  **Inpatient (T2)** | | | | | | | | | | | | | **Visit 4**  **(T3)** |
| --- | --- | --- | --- | --- | --- | --- | --- | --- | --- | --- | --- | --- | --- | --- | --- | --- | --- | --- | --- | --- | --- |
|  | Outpatient | Outpatient | | | | | | Day 1 | | | | | | | Day 2 | | | | | | Out |
| **Type of Visit** | Screening | Base | 0 | 2 | 4 | 6 | 8 | Base |  | 1 | 2 | 4 | 8 | 12 | 24 | 0 | 2 | 4 | 6 | 8 | 2 wks |
| Urine Analysis^1^ | **X** |  |  |  |  |  |  |  |  |  |  |  |  |  |  |  |  |  |  |  | **X** |
| Urine Toxicology^2^ | **X** |  |  |  |  |  |  |  |  |  |  |  |  |  |  |  |  |  |  |  |  |
| Urine Pregnancy^3^ | **X** | **X** |  |  |  |  |  | **X** |  |  |  |  |  |  |  |  |  |  |  |  | **X** |
| Urine Sp. Gravity^4^ | **X** |  |  |  |  |  |  | **X** |  | **X** | **X** | **X** | **X** | **X** | **X** |  |  |  |  | **X** |  |
| Urine pH^5^ | **X** |  |  |  |  |  |  | **X** |  | **X** | **X** | **X** | **X** | **X** | **X** |  |  |  |  | **X** |  |
| Urinary UA^6^ |  |  |  |  |  |  |  | **X** |  | **X** | | | | | **X** |  |  |  |  |  | **X** |

**1.** Complete urine analysis and sediment on morning spot urine.

**2.** General toxicology, including alcohol

**3.** Rapid pregnancy test

**4 and 5**. Specific gravity and pH can me measured in the clinic, recovering most of the urine used for the measure.

**6.** The only note here is for the 12 hours urine collection. The participant is asked to urinate before the administration of the study drug. All urines are collected. Last urination at 12 hours from the beginning of the infusion.

**6. RISKS/DISCOMFORTS**

Risk associated with the administration of UA solution: UA is a physiological constituent of cells and blood in humans. Based on published studies, I.V. administration of UA 1,000 mg and 500 mg in 250 ml of 0.1% lithium carbonate/ 4% dextrose solution over a 60 minute period was not associated with any adverse event, change in blood pH, clinical, hemodynamic and biochemical parameters. In a total of 44 persons, the administration of UA 500 mg was associated with an average increase of circulating levels between 3 and 4 mg/dL which creates a transitory increase of the UA concentration. The transitory increase of the UA concentration did not cause any risk, while it was associated with increased plasma antioxidant activity, reduced oxidative stress marker and better endothelial function. There is a very low risk that UA may precipitate in renal tubules and cause kidney damage. This risk is minimized by administering a dose of UA that should not increase the UA concentration above the solubility threshold, checking the urine specific gravity is below 1015 before the administration, ensuring abundant intake of water, inducing a mild urine alkalinitation (by administering orally sodium bicarbonate) and monitoring the urine pH and specific gravity.

Risk associated with the administration of Rasburicase: Potential side effects are listed in the table below. These side effects have been observed in 703 patients affected from leukemia, lymphoma, and solid tumor malignancies who are receiving anti-cancer therapy. The most severe side effects of Rasburicase administration are hypersensitivity reactions including anaphylaxis (<1%), and methemoglobinemia (<1%).

| **Serious Adverse Reaction** | Percentage (%) of Subjects (total n=703) |
| --- | --- |
| Allergic reactions including anaphylaxis | <1 |
| Rash | <1 |
| Hemolysis | <1 |
| Methemoglobinemia | <1 |
| Fever | 5 |
| Neutropenia with fever | 4 |
| Respiratory distress | 3 |
| Sepsis | 3 |
| Neutropenia | 2 |
| Mucositis | 2 |
| **Adverse Reaction** | Percentage (%) of Subjects (total n=436) |
| Vomiting | 50 |
| Fever | 46 |
| Nausea | 27 |
| Headache | 26 |
| Diarrhea | 20 |
| Mucositis | 15 |
| Rash | 13 |

Note: an antibody response to a single dose of rasburicase (immunogenicity) has also been observed. To the date of this protocol, the immunogenicity issue is not applicable to our study since the drug is not approved for the treatment of adults.

The risk of blood drawing: There is a slight risk of pain, bruising around the site where blood is drawn and rarely bleeding or infection. To minimize this risk, the routine blood–drawing protocol will be followed and pressure will be applied to the site. Some people experience feelings of lightheadedness or dizziness after having blood drawn. To reduce the risk of falling, we will monitor the subject closely and ask about these symptoms before we allow them to stand. The samples collected will be used to monitor the subject’s medical condition and for research purposes.

Risk of Basal Metabolic Rate: A little discomfort may be due to mask. However, this discomfort is only momentary, lasting only few minutes.

Risk of Oral Lipid Tolerance Test: Rarely eating a fatty meal may lead to a temporary sense of abdominal fullness and discomfort, nausea, vomiting, or diarrhea.

Risk of resting EKG: There may be slight skin irritation from the electrodes. There are no other risks associated with this test.

Risks of MRI, Functional MRI, and MRS. The effects of magnetic fields in an MR scanner have been extensively studied, and the risks with an MR exam are low, particularly if the subject qualify for the testing based upon the screening questions. Participants, however, may be bothered by feelings of confinement (claustrophobia), and by the noise made by the magnet during the procedure. To minimize discomfort, participants will be asked to wear earplugs or earphones while in the magnet. Participants cannot receive an MRI if they have a pacemaker, an implanted defibrillator or certain other implanted electronic or metallic devices. Every participant who qualify for MRI testing will be asked to report any history of brain surgery for a cerebral aneurysm, implanted medical or metallic devices, shrapnel, or other metal, such as metal in the eye. If the MRI compatibility of any metal implant cannot be established, it will be considered a contraindication.

Risk of Genetic Testing.

The risk of genetic testing is quite low because only genes that are related directly or indirectly with UA metabolism will be characterized in this protocol. Although there can be no absolute guarantees, every reasonable effort will be made to keep personally identifiable information secret so there will be no misuse. During the consenting process, we advise participants that if they are asked if ever been tested for a genetic disorder, they should respond “no” since the results of these tests are done for pure research purpose and are not systematically complemented by the collection of other medical information that make them clinically relevant. The privacy of the research information generated from this study are protected and considered highly confidential.

Risk/Benefit Assessment: There is a high societal Benefit/Risk Ratio in this study. We may be able to ascertain if UA increases pro-inflammatory biological substances or contrarily leads to a protective increase of antioxidant substances.

Risk Associated with Stored Specimens: The greatest risk associated with stored specimens is the unplanned release of information from the subject’s medical records. The chance that this information will be given to an unauthorized person without the subject’s permission is very small. Possible problems with the unplanned release of information include discrimination when applying for insurance or employment. Similar problems may occur if the subject discloses information himself or agrees to have his medical records released.

Risk Associated with HIV Testing:

This study requires that your blood be tested for blood-transmitted diseases such as hepatitis virus B, hepatitis virus C, and human immunodeficiency virus (HIV) during the screening visit. You will be informed of the results of these blood tests regardless of your participation in this study.

Human immunodeficiency virus (HIV) test: A separate consent for HIV testing will be explained to you and HIV test will be done during the screening visit (visit #1) only.

If your HIV test is positive:

- A copy of the Department of Health and Mental Hygiene’s publication “Information for HIV Infected Persons” will be provided;
- The local health department or your doctor will offer advice about services which are available;
- Information will be provided on how to keep from transmitting HIV infection;
- Your Unique Identifying Number (UI) will be given to the health department;
- Your name will be reported to the local health department when your doctor finds that you have symptoms of HIV disease or AIDS;
- The local health department or your doctor will offer assistance in notifying and referring your partners for services. If you refuse to notify your partners, your doctor may notify them or have the local health department do so. If local health department staff notifies your partners, your name will not be used. Maryland law requires that when the local health department knows of your partners, it must refer them for care, support, and treatment.

**7. SAFETY MEASURES**

Safety Measurement in Case of Adverse Events: Due to the double blind design of the study, the NIA/MRI Research Pharmacy will prepare and maintain the randomization schedule for the protocol. This randomization schema will be available at the NIA Clinical Research Unit Pharmacy. The Pharmacist will notify the PI of the specific participant’s randomization in case of any adverse events/emergency situations.

The definitions of adverse events (AEs) and serious adverse events (SAEs) are given below. All staff involved in this study will be familiar with the content of this section. The principal investigator is responsible for making sure that all personnel are adequately trained in the screening and recognition of these adverse events.

Adverse Event: An adverse event is the development of an undesirable medical condition or the deterioration of a pre-existing medical condition following or during exposure to a pharmaceutical product or medical intervention, whether or not considered causally related to the product. An undesirable medical condition can be symptoms (e.g., nausea, chest pain), signs (e.g., tachycardia, enlarged liver would this be noticed in this short period of time only PE is during visits 1 and 2, or the abnormal results of an investigation (e.g., laboratory findings, electrocardiogram). In the context of this study, the definition of an adverse event includes an undesirable medical condition occurring at any time, including run-in or washout periods, even if no study treatment has been administered.

Management of adverse event

In case of an emergent medical event the subject will be evaluated by the study NIA Clinical Research Unit medical team R.N., CRC, NP/PA, and MD. If needed the subject will be transferred to the Harbor Hospital emergency room and will receive appropriate evaluation and treatment.

Reporting of Adverse Events

All adverse clinical experiences, whether observed by the investigator or reported by the patient, must be recorded, with details about the duration and intensity of each episode, the action taken with respect to the test drug, and the patient’s outcome. The investigator must evaluate each adverse experience for its relationship to the test drug and for its seriousness. The investigator must appraise all abnormal laboratory results for their clinical significance. If any abnormal laboratory result is considered clinically significant, the investigator must provide details about the action taken with respect to the test drug and about the patient’s outcome.

Serious Adverse Event: A serious adverse event is an AE occurring during any study phase (e.g., run-in, treatment, washout, follow-up) and at any dose of the investigational product, comparator, placebo or medical intervention, that fulfills one or more of the following criteria:

- results in death
- is immediately life-threatening
- requires in-patient hospitalization or prolongation of existing hospitalization
- results in persistent or significant disability or incapacity
- results in a congenital abnormality or birth defect
- Any other important medical event that may jeopardize the subject or may require medical intervention to prevent one of the outcomes listed above.

The causality of SAEs (their relationship to study treatment) will be assessed by the investigator(s).

Reporting of Serious Adverse Events: Investigators and other site personnel will inform the FDA, via a MedWatch form, of any unexpected and possibly study drug related SAE that occurs according to the FDA reporting requirement timelines. A copy of the MedWatch report will be emailed to the MRI IRB through the NIA/IRP Protocol Office at the time the event is reported to the FDA. We are aware that it is the responsibility of the investigator to compile all necessary information and ensure that the FDA receives a report according to the FDA reporting requirement timelines and to ensure that these reports are also submitted to MRI IRB at the same time. A complete written SAE report will be sent with a cover page indicating the following:

- Rasburicase® or UA Investigator Sponsored Study (RISS)
- The investigator IND number assigned by the FDA
- The investigator’s name and address
- The trial name and the reference number

Sent to the NIA Clinical Research Protocol Office, Attention Rasburicase® or UA ISS Safety Representative. If a non-serious AE becomes serious, this and other relevant follow-up information must also be provided to the FDA.

**8. GRADING AND MANAGEMENT OF TOXICITY**

Management of Unwanted/Emergent Medical Events: In case of an emergent medical event the subject will be evaluated by the study/NIA Clinical Research Unit medical team R.N., CRC, NP/PA, and MD. If needed the subject will be transferred to the Harbor Hospital emergency room and will receive appropriate evaluation and treatment.

Reporting Serious and Unexpected Side Effects: Serious and unexpected side effects will be immediately reported to the principal investigator. He will inform the NIA Protocol Office and they will notify the MedStar IRB in the time frame mandated by their guidelines.

Serious and Unexpected Side Effects Data Collection and Evaluation: Data of serious and unexpected side effects will be collected separately. The principal investigator will monitor the data any time clinical/laboratory data is added, and at the end of a certain dose phase, will decide according to his clinical judgment whether there is need for stop the study.

**9. STUDY END POINTS AND MONITORING**

Primary end point

The primary outcome is to ascertain whether UA is a pro-inflammatory compound. This aim will be tested both by measuring the concentration of inflammatory markers after short term changes in UA, and by testing the hypothesis that different concentrations of UA modify the inflammatory response induced by a lipid tolerance test.

Secondary Objectives

To document the effect of increasing versus decreasing UA levels on circulating antioxidant biomarkers. To test the hypothesis that increasing levels of UA are associated with higher mitochondrial efficiency as indicated by higher MRI-spectroscopy estimated rate of phosphocreatinine resynthesis after exercise.

An accrual ceiling of 200 men and women aged 50-75 years is expected in order to assure we have 40 subjects completing the study. The subjects participating in the study will be monitored closely during the study as noted above and will be followed-up until UA plasma levels return to baseline.

**10. Criteria for Withdrawal of Subjects from the Study**

Participation in both study trials may be stopped by the P.I. or the National Institute on Aging without subject consent at any time if:

1. The P.I. believes that stopping participation is necessary for the subject’s health and safety

2. The subject fails to follow the study instructions

3. The National Institute on Aging decides to terminate the study

4. Administrative reasons require the subject to withdraw

5. The subject decides to withdraw from the study. Any data or blood collected until that point in time would remain part of the study.

A subject may stop participating at any time. However, we would encourage them to first talk to the researcher and their own doctor before the final decision.

**11. Statistical Considerations**

Sample size procedure specifications: as this is a pilot trial aimed at understanding the physiological parameters rather than clinical safety or efficacy endpoints, we estimated the sample size (number of subjects per arm; total number of subjects) as follows:

A. We determined the inter-assay coefficient of variation (CV) of the main laboratory assays of IL-6 which is the reference inflammatory marker identified in this study;

B. We considered that any value demonstrating a difference greater than 3 x CV in each laboratory parameter/endpoint between baseline and post-intervention (in both Trials A and B) would be of biological significance. The obtained CV was indirectly included in the sample size definition by affecting the variability of expected changes in UA we estimated to observe after treatment.

C. We estimated the sample size by Monte Carlo (41) study applied to a mixed effect model (42) using R software (43). The form of the mixed effects model was

**y(ij)=a+a0(i) + b1*factor(time(j))+b2*treatment(i)+ e(ij).**  In this model, ‘i’ refers to subject, j to time, and a0(i) to the random intercept for subject i. We aimed at estimating a big enough sample per arm to have a high likelihood of detecting a true difference between two groups.

These sample size procedure specification is based on previous findings from Waring and our group. Waring et al. showed that infusions of 1,000 mg and 500 mg of UA were associated with an increase of UA circulating levels of 3.2-4.9 mg/dL with this increase persisting for 10-12 hours. For the purpose of the power analysis, we assumed a very conservative increase in UA of 2.5-3 mg/dL persisting from 2 to 12 hours, and a progressive decline in UA levels after 2 hours returning to baseline by 48 hours. The control group was assumed to show no systematic change in UA (16,18, 19). Further, the report by Ruggiero et al found that IL6 increased by 0.08 per mg/dL increase of uric acid. We examined an associated increase of 0.04, 0.06 and 0.08 pg/ml for each mg/dL change in UA. Using these relationships, IL-6 was sampled at each point in time from a normal distribution with a standard deviation as found in Ruggiero et al. of 0.02 for a sample size of 976 subjects (21). The resulting IL-6 was entered into a mixed effects model being regressed on time and group with subject as a random effect. This model was compared to a model including group using a log-likelihood test, and the null was accepted at p> 0.05. The simulation was repeated 100 times to determine the number of times the null hypothesis was rejected. For 10 subjects per group, with a rise in UA of 2.7 mg/dL, a coefficient of 0.04 gave a power of 0.94, a coefficient of 0.06 gave a power of 0.98, and a coefficient of 0.08 gave a power of 1.00. For rasburicase, we postulated that UA would go to zero. With the current model, UA increase of 2.0 with progressive decrease over time gives power of 0.92, 0.93, 0.93 for the coefficients of 0.04, 0.06 and 0.08. UA increase of 1.5 had a power in the 0.6-0.7 range for the 3 coefficients.

With this assumption, 10 subjects per group resulted in a power of 0.97 for a coefficient of 0.04, 1.00 for a coefficient of 0.06, and 1.00 for a coefficient of 0.08.

Enrollment: a total of 40 participants will be enrolled. According to inclusion/exclusion criteria 20 participants, 10 men and 10 women, will be recruited in Trial A: Uric Acid versus placebo trial and 20 participants, 10 men and 10 women, in Trial B: Rasburicase versus placebo trial. To maintain the number of participants as above, we will screen 6 more subjects, (3 men and 3 women for each cohort), that will be enrolled only if some drop-outs occur.

Randomization to treatment or placebo: according to the order of enrollment and sex, participants will be randomly assigned to receive treatment or placebo in each cohort.

Data analysis: differences in circulating inflammatory markers and antioxidants, as well as differences in other measurements will be estimated before and after treatment in both cases and controls. In each trial, the time-dependent modification of UA levels will be evaluated and the outcomes will be estimated and compared between cases and controls after intervention.

**12. DATA MANAGEMENT**

All protocols at the NIA follow the NIA Data and Safety Monitoring Plan. This includes using the Level of Risk Assessment Monitoring Guidelines that has been established for the NIA following NIH rules and regulations to ensure good clinical practices in the conduct of good clinical research. This process may include establishing a Data Safety and Monitoring Board (DSMB) which is a group of experts not connected to the study to review the data from this research throughout the study. Participants will be informed about new information for this or other studies that may affect their health, welfare, or willingness to stay in this study.

Records will be kept using Clinical Management Software products called Study Manager and Oracle Clinical Remote Data Capture application (ver 4.5) which loads directly into Oracle Clinical database system or batch loaded into the database system from other electronic sources (i.e. laboratory results, EKG, BMR, questionnaires). This software is HIPAA (Health Insurance Portability and Accountability Act) compliant software developed by Advanced Clinical Software and by Oracle Corporation respectively. These databases are password protected and maintained on a secure NIA/NIH Internet with access limited to authorized NIA staff members. All NIA members who have access to these databases have the proper training on patient confidentiality as well as the required Human Subject Protection Training. The database servers are maintained by the Clinical Information and Data Management Section of the NIA and are located on their unit at Harbor Hospital.

We may also use the Cardiff Teleform Information Capture System for data collection and automated data entry. The Cardiff Teleform system produces machine-readable data collection forms that will be read by a dedicated scanner and entered into a secure, limited access database, maintained by the NIA.

Although participants may leave the study at any time, any data or blood collected until that point in time will remain part of the study database. All data and blood collected will be available only to authorized staff working on this protocol.

**13. COLLECTION AND STORING OF HUMAN SAMPLE SPECIMENS**

Intended Use of the Sample/Data: Samples and data collected under this protocol may be used to study baseline health of the participant and laboratory values will be used to investigate inflammatory and antioxidant markers before, during and after treatment as above specified. Genetic testing will be preformed. Any other research or experimental treatments will be done under other protocols for which separate IRB review and approval will be obtained.

Labeling of Stored Samples: The subject’s stored samples will be labeled at the time of collection with the participants study code, visit number and visit date so that only the study team can link them to the subject. Any identifying information about the subject will be kept confidential to the extent required by law.

How Samples/Data will be tracked: Samples/data will be tracked using the NIA Biological Sample Inventory system following NIH guidelines. Any other research that may use the banked samples other than the research described in this protocol will be done under other protocols for which a separate IRB review and approval will be obtained.

Storage and Release of Samples: Samples of the subject’s blood will be kept in a research laboratory at the National Institutes of Aging, NIH, or one of our contract facilities. The subject’s samples may be tested immediately, or they may be frozen and analyzed later. Samples may be kept until no residual specimens remain, or until the investigators decide to destroy them. If the subject gives us permission, some samples may be released to other doctors and scientists who are not associated with this institute. The Clinical Director and the Principal Investigator on this protocol will decide which researchers may receive samples. The subject’s samples may be used in their research only if the research has been approved by an Institutional Review Board (IRB) and is related to the original research questions associated with this protocol.

We will code the blood and urine samples so that we will keep the subject’s identity confidential. We will retain a code list that enables us to link the clinical information and results on each sample to the subject. The biological assays performed on these samples are described in the protocol.

Stored Samples and Future Studies: Samples and data collected under this protocol may be used to study the biological effects of acutely increasing or decreasing UA levels. Part of the plasma and serum obtained from blood samples will be stored at -80 C for future use, in order to repeat any needed tests or for the performance of any new test that can help in understanding the biological effect of acutely increasing or decreasing UA blood levels.

What will happen to the Samples/Data at the Completion of the Protocol?: In the future, other investigators (both at the NIH and outside) may wish to study these samples/data to address scientific questions different from the ones described in this protocol. In this case, IRB approval will be sought prior to any sharing of samples. Any clinical information shared about the sample with or without patient identification would similarly require prior IRB approval. The research use of stored, unlinked or unidentified samples may be exempt from the need for prospective IRB review and approval. Exemption request will be submitted in writing to the MedStar IRB, which is authorized to determine whether a NIA research activity is exempt.

At the completion of the protocol, when all analyses and assays relevant to this project are completed, samples and data will either be destroyed, or after IRB approval, transferred to another existing protocol or repository.

The stored material will be used only for research and will not be sold. The research done with the subject’s materials may be used to develop new products in the future, but the subject will not receive payment for such products.

Circumstances that Prompt PI to Report to IRB the Loss or Destruction of Samples/Data: The NIH Intramural Protocol Violation definition related to loss of samples (for example, due to freezer malfunction) will be followed in reporting to the IRB. The violation compromises the scientific integrity of the data collected for the study. Any loss or unanticipated destruction of sample (for example, due to freezer malfunction) or data (for example, misplacing a printout of data with identifiers) will be reported to the IRB. Additionally, subjects may decide at any point not to have their samples stored. In this case, the PI will destroy all known remaining samples and report what was done to both the subject and the IRB. This decision may not affect the subject’s participation in this protocol or any other protocols at NIH.

If the subject prefers to discontinue participation in this study, he/she may request that any samples with his/her name on them to be destroyed. The subject will not be asked for further information or samples. However, the resulting data from the study will not be destroyed.

**14. PHARMACEUTICAL INFORMATION**

**Uric Acid**

Description: UA I.V. solution is manufactured by Florida Biologix, Center of Excellence for Regenerative Health Biotechnology, University of Florida, USA (see ***Appendix A***). The description of UA chemistry and physiology in humans is reported in ***Appendix B***.

Dose: UA 500 mg in 250 ml of 0.1% lithium carbonate/4% dextrose solution will be administered intravenously over a 60 minute period. Before administration and on the same day, UA solution will be filtered for both UA microcrystals using a 0.22 mm Millex filter (Millipore, Molsheim, France), and for virus using the Millipore Viresolve NFR  (OptiScale-25 Small Volume Disposable Capsule Filter). The Millipore in-line filter will be done generating the required 20-50 psi of pressure for fluid passage that will be created using a peristaltic pump. The vehicle was selected because it allows stable dissolution of UA and theoretically should not affect serum antioxidant capacity.

Route of Administration: intravenous administration of reconstituted formula.

Clinical pharmacology: UA was administered intravenously in two previous clinical studies conducted at the Clinical Pharmacology Unit and research Center, The University of Edinburgh, UK. Infusion of 1,000 mg of UA in a 500 ml 4% dextrose/0.1% lithium carbonate vehicle led to a time-dependent UA increment of about 3.2-4.9 mg/dL. Serum UA concentration exhibited a two-component decay with a mean elimination half-life of 10.8 h. The elimination and distribution phase after infusion of 1,000 mg of UA has been previously described (16).

Drug Interactions: no data are available.

Toxicities: UA is a physiological constituent of the cell cytosol and a byproduct of nucleotide metabolism in humans. Biochemical evidence suggests that UA is a Reactive Oxygen Species scavenger (44-46). Phase 1 studies of UA infusion have been performed in 52 subjects. In the first study, 8 healthy non-smokers aged 24±2years were administered with a UA 1,000 mg in 250 ml of 0.1% lithium carbonate/ 4% dextrose I.V. solution over a 60 minute period. In the second study, 20 community healthy persons aged 23±3years were treated with a UA 500 mg in 250 ml of 0.1% lithium carbonate/ 4% dextrose I.V. solution over a 60 minute period. In the third study, 8 adult smokers (30 yrs), 8 adults affected from Type 1 diabetes (30 yrs) and 8 adult controls (30 yrs) were infused with UA 1,000 mg in 500 ml of 0.1% lithium carbonate/ 4% dextrose over a 60 minute period. No severe adverse events, adverse events nor change in clinic, hemodynamic and biochemical parameters have been reported in these studies (16,17,18,19).

Storage: UA solution will be securely stored in the NIA Research Pharmacy, and under the appropriate conditions. Stability tests will be performed by Florida Biologix at 5 times points over an 18 month period (3, 6, 9, 12 and 18 months).

Mechanism of Drug Accountability: All dispensing of UA and any destruction of the chemical doses will be documented on a protocol specific Investigational Agent Accountability Record, maintained in the NIA Research Pharmacy.

**Rasburicase (Elitek®)**

Description: rasburicase is a recombinant urate oxidase produced by a genetically modified Saccharomyces cerevisiae strain. The cDNA coding for rasburicase was cloned from a strain of *Aspergillus flavus*. Rasburicase catalyzes the enzymatic oxidation of UA into allantoin which is a soluble and inactive metabolite. This drug is manufactured and donated for the purpose of this study by Sanofi-Synthelabo, Inc. Drug product is a sterile, white to off-white, lyophilized powder intended for intravenous administration following reconstitution. Rasburicase is supplied in 3-mL colorless, glass vials containing 1.5 mg rasburicase, 10.6 mg mannitol, 15.9 mg L-alanine, and between 12.6 and 14.3 mg of dibasic sodium phosphate. The diluent solution for reconstitution, supplied in a 2 mL clear, glass ampule, is composed of 1.0 mL sterile Water for Injection, USP, and 1.0 mg Poloxamer 188.

Clinical pharmacology: pharmacokinetics of rasburicase were evaluated in two studies that enrolled patients with lymphoid leukemia (B and T cell), non-Hodgkin’s lymphoma (including Burkitt’s lymphoma) or acute myelogenous leukemia.

Data from 3 studies (Study 1 including 52 pediatric patients with acute leukemia or Non-Hodgkin’s Lymphoma, Study 2 including 89 pediatric and 18 adult patients with heamatologic malignancies, and Study 3 including 130 pediatric patients and 1 adult patient with leukemia or lymphoma) were pooled and analyzed to estimate the effect of Rasburicase over time on UA (46-48).

The pre-treatment plasma UA concentration was >8 mg/dL in 61 patients and was <8 mg/dL in 200 patients. Among patients with pre-treatment UA >8.0 mg/dL [baseline median 10.6 mg/dL (range 8.1 – 36.4)], the median per-patient decline in plasma UA concentration by 4 hours after treatment was 9.1 mg/dL (0.3 – 19.3 mg/dL). Among the patients with a pre-treatment, plasma UA level <8 mg/dL [baseline median 4.6 mg/dL (range 0.2 – 7.9 mg/dL)], the median per-patient decline in plasma UA concentration by 4 hours after treatment was 4.1 mg/dL (0.1 – 7.6 mg/dL).

Rasburicase exposure, as measured by AUC0-24 hr and Cmax, tended to increase linearly with doses over a limited dose range (0.15 to 0.20 mg/kg). The mean volume of distribution was 110 to 127 mL/kg in pediatric patients. Overall elimination half-life was 18 hours. No accumulation of rasburicase was observed between days 1 and 5 of dosing. According to the mechanism of action of Rasburicase, an overdose will lead to low or undetectable plasma UA concentration, which has no known clinical consequences (47-49).

Based on these studies, there are insufficient data to characterize pharmacokinetics in adult and older persons. Five of the 19 adults, among 265 patients enrolled, were age 65 or greater. However, the safety and efficacy of rasburicase I.V. administration in adults has been already proven. In the GRAAL1 study, 100 adult patients with aggressive Non-Hodgkin Lymphoma (NHL) received Rasburicase 0.20 mg/Kg/day for 3 to 7 days beginning before or the day of chemotherapy regimens with AEVB (43%), CHOP (44%), ACE (6%), COP-COPADEM (4%) and ESHAP (3%). About 95 patients treated at least 3 days responded to treatment; response was defined as normalization of UA levels throughout therapy and associated normalization of creatinine and other metabolites. The overall tolerance was excellent: 4 patients discontinued rasburicase earlier than 3 days: 3 of the 4 patients had an increase in the liver enzymes, and the other patient experienced NHL-related gastrointestinal hemorrhage. In three patients rasburicase treatment was stopped earlier than 3 days because of an increase in liver enzymes. This National Cancer Institute grade 3 toxicity was detected during the first 24 to 48 hours of treatment and was rapidly reversible within a few days in all three patients, without sequelae. This toxicity was attributed by the investigators either to rasburicase or to chemotherapy, given that a rapid and transient increase of liver enzymes may be observed and has been reported as part of the biologic changes after anthracycline-containing chemotherapy in patients with diffuse large-cell lymphoma. Rasburicase treatment was discontinued in one patient after 2 days of treatment because of NHL-related complications (gastrointestinal haemorrhage) (50). In the North American Compassionate Use Trial, 682 children and 387 adults received rasburicase 0.20 mg/Kg/day and 658 children and 338 adults were evaluable for efficacy. The median ages of the pediatric and adult populations were 7 and 54 years, respectively. The median number of doses and days of treatment were three. About 40.0% of children and 20.4% of adults received treatment for 5 or more days. The incidence of toxicities among pediatric and adult patients was 1.6% and 0.5% (51).

Rasburicase has been used in France and Italy for almost 3 decades. An international report from Bosly et al. showed that in 112 adult patients (28%) (31 were older than age 65 years) affected from malignancies, such as acute myeloid leukemia (38%) and non-Hodgkin lymphoma (29%), the reduction of plasma UA to normal levels within 4 hours was quite similar for adults and children in both the prophylaxis and treatment (52). Rasburicase was well tolerated, with mild headache being the most common side effect. Similar results are from the U.S. compassionate access program (53) and from a completed Phase II study in adult patients with lymphoma (50) constituting a substantial body of evidence regarding the efficacy and safety of rasburicase in adults.

Indication for Use: Rasburicase is administered as a 30-minute infusion once daily at a dose of 0.15 or 0.20 mg/kg/day (maximum total daily dose 0.20-0.40 mg/kg/day) in the initial management of higher plasma UA levels in pediatric patients with leukemia, lymphoma, and solid tumor malignancies who are receiving anti-cancer therapy.

Dose : All participants in this study will be administered a single dose of Rasburicase equal to **0.15 mg/kg/day** intravenously over 60 minutes. The product reconstituted with diluent is a clear, colorless solution. The reconstituted solution will not be shaken and will be inspected visually for particulate matter and discoloration prior to administration. The solution will be diluted in 250-mL of preservative-free NaCl 0.9% and is infused over 60 minutes without filtration. The reconstituted rasburicase must be administered within 24 hours of reconstitution.

Note that the dose of **0.15 mg/kg/day** is substantially lower that the dose already approved from FDA in pediatric and adult patients. This cautious approach was selected because we do not have sufficient data to determine whether geriatric subjects respond differently from pediatric subjects to rasburicase administration.

Route of Administration: intravenous administration of reconstituted formula.

Drug Interactions: No studies of interactions with other drugs have been conducted in humans. Rasburicase does not induce allopurinol, cytarabine, methylprednisolone, methotrexate, 6-mercaptopurine, thioguanine, etoposide, daunorubicin, cyclophosphamide or vincristine metabolism *in vitro*. In preclinical *in vivo* studies, rasburicase did not affect the activity of isoenzymes CYP1A, CYP2A, CYP2B, CYP2C, CYP2E, and CYP3A, suggesting no induction nor inhibition potential. Clinically relevant P450-mediated drug-drug interactions are therefore not anticipated in patients treated with the recommended rasburicase dose and dosing schedule.

Toxicities: The safety and efficacy of Rasburicase has been established well for a single course of treatment, which is how rasburicase will be used in this study. Potential side effects of Rasburicase reported here reflect data collected from 703 patients enrolled in multiple studies [63% male, 37% female; median age 10 years (range 10 days to 88 years); 73% Caucasian, 9% African, 4% Asian, 14% other/unknown]. Participants were affected by leukemia, lymphoma, and solid tumor malignancies and were receiving anti-cancer therapy in parallel with Rasburicase. Therefore, the adverse reaction rates observed in these clinical trials may not reflect the rates observed in practice in healthy subjects. However, the adverse reactions observed in these trials provides a basis for identifying the adverse events that may be related to drug use and to approximate rates.

Rasburicase adverse reactions, regardless of severity, were collected in 347 patients (265 pediatric and 82 adults) enrolled in one active-controlled trial, two uncontrolled trials and one uncontrolled safety trial (n=82). In a successive experience with 356 patients, Rasburicase adverse reactions were limited to serious adverse reactions.

Among the 703 patients the most serious adverse reactions were allergic reactions including anaphylaxis (<1%), rash (1%), hemolysis (<1%), and methemoglobinemia (<1%). Signs and symptoms of these reactions include bronchospasm, chest pain and tightness, dyspnea, hypoxia, hypotension, shock, and/or urticaria. Rasburicase administration should be immediately and permanently discontinued in any patient identified as having developed hypersensitivity reactions, hemolysis[[1]](#footnote-2) or methemoglobinemia[[2]](#footnote-3). Additional adverse reactions were fever (5%), neutropenia with fever (4%), respiratory distress (3%), sepsis (3%), neutropenia (2%), and mucositis (2%). The following adverse reactions were observed in <1% of patients regardless of causality: acute renal failure, arrhythmia, cardiac failure, cardiac arrest, cellulitis, cerebrovascular disorder, chest pain, convulsions, cyanosis, diarrhea, dehydration, hot flashes, ileus, infection, intestinal obstruction, hemorrhage, myocardial infarction, paresthesia, pancytopenia, pneumonia, pulmonary edema, pulmonary hypertension, retinal hemorrhage, rigors, thrombosis, and thrombophlebitis.

Immunogenicity: Rasburicase is immunogenic in healthy volunteers, and can elicit antibodies that inhibit the activity of rasburicase *in vitro.* In a study of 28 healthy volunteers, the incidence of antibody responses to either a single dose or to 5 daily doses was assessed. Binding antibodies to rasburicase were detected by ELISA in 17/28 (61%) volunteers and neutralizing antibodies were detected in 18/28 (64%) volunteers. Time to detection of antibodies ranged from 1 to 6 weeks after ELITEK exposure. In two subjects with extended follow-up, antibodies persisted for 333 and 494 days.

Storage: Rasburicase will be securely stored in the NIA Research Pharmacy under the appropriate conditions.

Mechanism of Drug Accountability: All dispensing of Rasburicase will be documented on a Protocol Specific Investigational Agent Accountability Record maintained in the NIA Research Pharmacy.

**15. HUMAN SUBJECT PROTECTION**

It is National Institute on Aging (NIA) policy that the regulations of the Department of Health and Human Services (HHS), set forth in 45 CFR Part 46, are applicable to all research involving human subjects, as defined by these regulations, for which the NIA is responsible, regardless of the source of funding or whether the research is funded. In the case of conflict between regulations of the funding or regulatory agency and HHS, the more restrictive regulations shall prevail. The NIA is also obligated by law to adhere to the regulations of the Food and Drug Administration (21 CFR Parts 50 and 56) governing projects involving investigational new drugs [within the meaning of 21 U.S.C. sections 355(i) or 357(d)], or investigational new devices [within the meaning of 21 U.S.C. section 360(g)].

**16. RECRUITMENT PLAN**

Subjects will be recruited for this study from clinics and health care facilities in the Baltimore-Washington area. Brochures, flyers and other forms of public advertisements will be used. Recruitment letters may be mailed to health care providers and to those participants who have requested information. We may place advertisements and/or public service announcements (PSA) on websites, in radio broadcasts, television, as well as newspapers, newsletters, and magazines. Direct mailing through established marketing and advertising organizations may also be used. We may also use public talks in the community. All information distributed for recruitment purposes will be the equivalent of information contained in ClinTrials.gov or from IRB-approved flyers or brochures. We will not be recruiting participants affiliated with the NIA or from the NIA Clinical Research unit. We feel that we will effectively recruit and retain subjects during the study without difficulty due to the comprehensive testing and follow-up provided.

Inclusion of Women and Minorities: Study Population - Gender/Ethnic Inclusion - Exclusions are based solely on medical grounds. An equal number of men and women will be recruited for this study. The racial and ethnic characteristics of the proposed participant population reflect the demographics of Baltimore and the surrounding area. No potential participant will be excluded on the basis of race.

**17. BENEFITS**

This is not a treatment study, and the subjects are not expected to receive any direct medical benefits from their participation in the study. However, there is an expectation that the information from this study may lead to a better understanding about the relationship between UA and chronic inflammation. The comprehensive testing done through the study will provide valuable information to the subjects involved. Only results of tests that are medically relevant, mostly those collected in the eligibility phase of the study, will be communicated to the participants.

**18. COMPENSATION**

The amount of payment to research volunteers is guided by the National Institutes of Health policies.  In general, patients are not paid for taking part in research studies at the National Institutes of Health. Reimbursement of travel and subsistence will be offered consistent with NIH guidelines. Participants will receive monetary compensations up to $1,000.00, if they complete the entire study and it is distributed as follows: $150.00 for visit 2 and 4, $200.00 for visit 3 and $500 at the completion of the study. A meal ticket may be offered on visits that last longer than 6 hours or if an Oral Lipid Tolerance Test (OLTT) tests is being done.

**19. CONFLICT OF INTEREST**

The Principal Investigator, Associate Investigators and all research staff associated with this

protocol are in compliance with federal regulation Title 45 CFR Part 50, Subpart F to promote

objectivity in research by maintaining standards to ensure there is no reasonable expectation that

the design, conduct, or reporting of research funded under NIA/NIH will be biased by any

conflicting financial interest of an Investigator.

**20. CONFIDENTIALITY**

When results of an NIA/NIH research study are reported in medical journals or at scientific meetings, the people who take part are not named or identified. In most cases, the NIA/NIH will not release any information about the participant’s research involvement without their written permission. However, if they sign a release form, the NIA/NIH will release information from their medical record. This information might effect (either favorably or unfavorably) the outcome of the event that triggered the request for release of information.

In research collaborations, all subjects will be identified by a code rather than by their name. The results are research material that is strictly confidential. Information will not be released without the subject’s consent, but may be reviewed by other researchers for scientific purposes.

**21. CONSENT PROCESS**

A document for informed consent is attached, written in language understandable to someone who has not completed high school (8^th^ grade level). The consent process will be conducted by the study coordinator, Principal investigator, associated investigators or any other NIA designated consenters. Each participant will receive a verbal explanation of what is involved in participating in the protocol for which they are being screened, including risks and discomforts. After the study procedures have been explained in detail, the prospective participants will have an opportunity to ask questions regarding their responsibilities and all concerns about participation will be addressed. When the participant fully understands his/her responsibilities and possible risks and discomforts and agrees to participate in the current study they will be provided with the consent to sign, as well as the original will be placed in their medical records.

The trial will be conducted in compliance with the protocol, HHS, FDA, ICH and all applicable institutional, state and local requirements.

**References**

1. Nakanishi N, Okamoto M, Yoshida H, Matsuo Y, Suzuki K, Tatara K. Serum UA and risk for development of hypertension and impaired fasting glucose or Type II diabetes in Japanese male office workers. Eur J Epidemiol. 2003; 18:523-30.
2. Carnethon MR, Fortmann SP, Palaniappan L, Duncan BB, Schmidt MI, Chambless LE. Risk factors for progression to incident hyperinsulinemia: the Atherosclerosis Risk in Communities Study, 1987-1998. Am J Epidemiol. 2003; 158:1058-67.
3. Kahn HA, Medalie JH, Neufeld HN, Riss E, Goldbourt U. The incidence of hypertension and associated factors: the Israeli ischemic heart disease study. Am Heart J. 1972; 84: 171–182.
4. Selby JV, Friedman GD, Quesenberry CP. Precursors of essential hypertension: pulmonary function, heart rate, UA, serum cholesterol, and other serum chemistries. Am J Epidemiol. 1990; 131: 1017–1027.
5. Jossa F, Farinaro E, Panico S, Krogh V, Celentano E, Galasso R, Mancini M, Trevisan M. Serum UA and hypertension: the Olivetti heart study. J Hum Hypertens. 1994; 8: 677–681.
6. Weir CJ, Muir SW, Walters MR, Lees KR. Serum urate as an independent predictor of poor outcome and future vascular events after acute stroke. Stroke. 2003; 34:1951-6.
7. Alderman MH, Cohen H, Madhavan S, Kivlighn S. Serum UA and cardiovascular events in successfully treated hypertensive patients. Hypertension. 1999; 34:144-50.
8. Anker SD, Doehner W, Rauchhaus M, Sharma R, Francis D, Knosalla C, Davos CH, Cicoira M, Shamim W, Kemp M, Segal R, Osterziel KJ, Leyva F, Hetzer R, Ponikowski P, Coats AJ. UA and survival in chronic heart failure: validation and application in metabolic, functional, and hemodynamic staging. Circulation. 2003;107:1991-7.
9. Doehner W, Anker SD. Xanthine oxidase inhibition for chronic heart failure: is allopurinol the next therapeutic advance in heart failure? Heart. 2005; 91:707-9.
10. Niskanen LK, Laaksonen DE, Nyyssonen K, Alfthan G, Lakka HM, Lakka TA, Salonen JT. UA level as a risk factor for cardiovascular and all-cause mortality in middle-aged men: a prospective cohort study. Arch Intern Med. 2004; 164:1546-51.
11. Hansson GK. Inflammation, atherosclerosis, and coronary artery disease. N Engl J Med. 2005;352:1685-95.
12. Festa A, Haffner SM. Inflammation and cardiovascular disease in patients with diabetes: lessons from the Diabetes Control and Complications Trial. Circulation. 2005; 111:2414-5.
13. Skoog T, Dichtl W, Boquist S, Skoglund-Andersson C, Karpe F, Tang R, Bond MG, de Faire U, Nilsson J, Eriksson P, Hamsten A. Plasma tumour necrosis factor-alpha and early carotid atherosclerosis in healthy middle-aged men. Eur Heart J. 2002; 23:376-83.
14. Levine B, Kalman J, Mayer L, Fillit HM, Packer M. Elevated circulating levels of tumor necrosis factor in severe chronic heart failure. N Engl J Med. 1990; 323: 236–241
15. Frohlich M, Imhof A, Berg G, Hutchinson WL, Pepys MB, Boeing H, Muche R,Brenner H, Koenig W. Association between C-reactive protein and features of the metabolic syndrome:a population-based study. Diabetes Care. 2000; 23:1835-9.
16. Waring WS, Webb DJ, Maxwell SR. Systemic UA administration increases serum antioxidant capacity in healthy volunteers. J Cardiovasc Pharmacol. 2001; 38:365-71.
17. Waring WS, Webb DJ, Maxwell SR. Lithium carbonate as a potential pharmacological vehicle: intravenous kinetics of single-dose administration in healthy subjects. Eur J Clin Pharmacol. 2002; 58:431-439
18. Waring WS, Convery A, Mishra V, Shenkin A, Webb DJ, Maxwell SR. UA reduces exercise-induced oxidative stress in healthy adults. Clin Sci (Lond). 2003;105:425-30.
19. Waring WS, McKnight JA, Webb DJ, Maxwell SR. Uric acid restores endothelial function in patients with type 1 diabetes and regular smokers. Diabetes. 2006 Nov;55(11):3127-32.
20. Shi Y, Evans JE, Rock KL. Molecular identification of a danger signal that alerts the immune system to dying cells. Nature. 2003; 425: 516-21.
21. Ruggiero C, Cherubini A, Ble A, Bos AJ, Maggio M, Dixit VD, Lauretani F, Bandinelli S, Senin U, Ferrucci L. Uric acid and inflammatory markers. Eur Heart J. 2006 M.
22. Ruggiero C, Cherubini A, Miller E 3rd, Maggio M, Najjar SS, Lauretani F, Bandinelli S, Senin U, Ferrucci L. Usefulness of uric acid to predict changes in C-reactive protein and interleukin-6 in 3-year period in Italians aged 21 to 98 years. Am J Cardiol. 2007 Jul 1;100(1):115-21.
23. Kanellis J, Kang DH. UA as a mediator of endothelial dysfunction, inflammation, and vascular disease. Semin Nephrol. 2005; 25:39-42.May;27(10):1174-81.
24. Kuzuya M, Ando F, Iguchi A, Shimokata H. Effect of aging on serum UA levels: longitudinal changes in a large Japanese population group. J Gerontol A Biol Sci Med Sci. 2002; 57:660-4.
25. Ferrucci L, Corsi A, Lauretani F, Bandinelli S, Bartali B, Taub DD, Guralnik JM, Longo DL. The origins of age-related proinflammatory state. Blood. 2005; 105: 2294-9.
26. Kang DH, Nakagawa T, Feng L, Watanabe S, Han L, Mazzali M, et al. A role for UA in the progression of renal disease. J Am Soc Nephrol. 2002; 13:2888-97.
27. Kanellis J, Johnson RJ. Editorial comment--Elevated UA and ischemic stroke: accumulating evidence that it is injurious and not neuroprotective. Stroke. 2003;34:1956-7.
28. Johnson RJ, Kang DH, Feig D, Kivlighn S, Kanellis J, Watanabe S, Tuttle KR, Rodriguez-Iturbe B, Herrera-Acosta J, Mazzali M. Is there a pathogenetic role for UA in hypertension and cardiovascular and renal disease? Hypertension. 2003;41:1183-90.
29. Kanellis J, Watanabe S, Li JH, Kang DH, Li P, Nakagawa T, Wamsley A, Sheikh-Hamad D, Lan HY, Feng L, Johnson RJ. Uric acid stimulates monocyte chemoattractant protein-1 production in vascular smooth muscle cells via mitogen-activated protein kinase and cyclooxygenase-2. Hypertension. 2003 Jun;41(6):1287-93.
30. Watanabe S, Kang DH, Feng L, Nakagawa T, Kanellis J, Lan H, et al. UA, hominoid evolution, and the pathogenesis of salt-sensitivity. Hypertension. 2002; 40:355-60.
31. Pfeffer KD, Huecksteadt TP, Hoidal JR. Xanthine dehydrogenase and xanthine oxidase activity and gene expression in renal epithelial cells. Cytokine and steroid regulation. J Immunol. 1994 Aug 15; 153(4):1789-97.
32. Rinaldo JE, Clark M, Parinello J, Shepherd VL. Nitric oxide inactivates xanthine dehydrogenase and xanthine oxidase in interferon-gamma-stimulated macrophages. Am J Respir Cell Mol Biol. 1994; 11:625-30.
33. Page S, Powell D, Benboubetra M, Stevens CR, Blake DR, Selase F, Wolstenholme AJ, Harrison R. Xanthine oxidoreductase in human mammary epithelial cells: activation in response to inflammatory cytokines. *Biochim Biophys Acta.* 1998; 1381:191-202.
34. Wright RM, Ginger LA, Kosila N, Elkins ND, Essary B, McManaman JL, Repine JE. Mononuclear phagocyte xanthine oxidoreductase contributes to cytokine-induced acute lung injury. *Am J Respir Cell Mol Biol.* 2004; 30:479-90.
35. Komaki Y, Sugiura H, Koarai A, Tomaki M, Ogawa H, Akita T, Hattori T,Ichinose M. Cytokine-mediated xanthine oxidase upregulation in chronic obstructive pulmonary disease's airways. *Pulm Pharmacol Ther.* 2005; 18:297-302.
36. Martindale JL, Holbrook NJ. Cellular response to oxidative stress: signaling for suicide and survival. *J Cell Physiol*. 2002; 192:1-15.
37. Seeberg E. Oxidative DNA damage and repair-Implication for aging. In Aging at the molecular level. In Von Zglinicki T. Biology of aging and its modulation. I. 2003.
38. Solaroglu I, Okutan O, Kaptanoglu E, Beskonakli E, Kilinc K. Increased xanthine oxidase activity after traumatic brain injury in rats. J Clin Neurosci. 2005 Apr;12(3):273-5.
39. Chambers JC, McGregor A, Jean-Marie J, Obeid OA, Kooner JS. Demonstration of rapid onset vascular endothelial dysfunction after hyperhomocysteinemia: an effect reversible with vitamin C therapy. Circulation. 1999; 99:1156-60.
40. Williams MJ, Sutherland WH, McCormick MP, de Jong SA, Walker RJ, Wilkins GT. Impaired endothelial function following a meal rich in used cooking fat. J Am Coll Cardiol. 1999; 33:1050-5.
41. Muthén, L.K. and Muthén, B.O. (2002). How to use a Monte Carlo study to decide on sample size and determine power. Structural Equation Modeling, 4, 599-620.
42. Pinheiro JC, Bates DM. Mixed-Effects Models in S and S-Plus. Springer-Verlag, New York, 2002.
43. R Development Core Team, R: A language and environment for statistical computing, R Foundation for Statistical Computing, Vienna, Austria. ISBN 3-900051-07-0, URL [http://www.R-project.org](http://www.R-project.org/).
44. Ames BN, Cathcart R, Schwiers E, et al. UA provides an antioxidant defense in humans against oxidant- and radical-caused aging and cancer: a hypothesis. Proc Natl Acad Sci U S 1981; 78:6858-62.
45. Nieto FJ, Iribarren C, Gross MD, et al. UA and serum antioxidant capacity: a reaction to atherosclerosis? Atherosclerosis 2000; 148: 131–139.
46. Glantzounis GK, Tsimoyiannis EC, Kappas AM, et al. UA and oxidative stress. Curr Pharm Des 2005; 11: 4145-51.
47. Goldman SC, Holcenberg JS, Finklestein JZ. A randomized comparison between rasburicase and allopurinol in children with lymphoma or leukemia at high risk for tumor lysis. Blood. 2001;97:2998-3003. J. Clin. Oncol. 2001 Feb 1;19(3):697-704.
48. Pui CH, Mahmoud HH, Wiley JM, Woods GM, Leverger G, Camitta B, Hastings C, Blaney SM, Relling MV, Reaman GH. Recombinant urate oxidase for the prophylaxis or treatment of hyperuricemia in patients With leukemia or lymphoma.
49. National Cancer Institute Common Toxicity Criteria, Version 2.0 (<http://ctep.cancer.gov/reporteing/ctc.html>)
50. Coiffier B, Mounier N, Bologna S, FermÃ© C, Tilly H, Sonet A, Christian B, Casasnovas O, Jourdan E, Belhadj K, Herbrecht R; Groupe d'Etude des Lymphomes de l'Adulte Trial on Rasburicase Activity in Adult Lymphoma .Efficacy and safety of rasburicase (recombinant urate oxidase) for the prevention and treatment of hyperuricemia during induction chemotherapy of aggressive non-Hodgkin's lymphoma: results of the GRAAL1 (Groupe d'Etude des Lymphomes del'Adulte Trial on Rasburicase Activity in Adult Lymphoma) study. J. Clin. Oncol. 2003 Dec 1;21(23):4402-6.
51. Jeha S, Kantarjian H, Irwin D, Shen V, Shenoy S, Blaney S, Camitta B, Pui CH. Efficacy and safety of rasburicase, a recombinant urate oxidase (Elitek), in the management of malignancy-associated hyperuricemia in pediatric and adult patients: final results of a multicenter compassionate use trial.Leukemia. 2005 Jan;19(1):34-8.
52. Bosly A, Sonet A, Pinkerton CR, McCowage G, Bron D, Sanz MA, Van den Berg H. Rasburicase (recombinant urate oxidase) for the management of hyperuricemia in patients with cancer: report of an international compassionate use study. Cancer. 2003 Sep 1;98(5):1048-54.
53. Pui CH, Jeha S, Irwin D, et al. Recombinant urate oxidase (rasburicase, Elitek) for prevention and treatment of malignancy-associated hyperuricemia: updated results of a compassionate use trial [abstract]. Blood. 2002;100(11 Suppl 1):556a.

1. Risk of hemolysis will be continuously monitored by bilirubin, LDH, haptoglobin levels during the 48 hours after treatment [↑](#footnote-ref-2)
2. Methemoglobin should be suspected in patients with hypoxic symptoms who appear cyanotic but have a PaO2 sufficiently high that hemoglobin should be fully saturated with oxygen. The diagnostic test of choice is measurement of the methemoglobin content, which is usually available on an emergency basis. Intravenous injection of 1 mg/kg of [methylene blue](javascript:showDrugInfo(390);) is effective emergency therapy. Milder cases and follow-up of severe cases can be treated orally with methylene blue (60 mg three to four times each day) or [ascorbic acid](javascript:showDrugInfo(42);) (300 to 600 mg/d). [↑](#footnote-ref-3)
